# A cost-effectiveness analysis of lisdexamfetamine dimesylate in the treatment of adults with attention-deficit/hyperactivity disorder in the UK

**DOI:** 10.1007/s10198-016-0864-4

**Published:** 2017-01-16

**Authors:** Evelina A. Zimovetz, Alain Joseph, Rajeev Ayyagari, Josephine A. Mauskopf

**Affiliations:** 1 RTI Health Solutions, 2nd Floor, The Pavilion, Towers Business Park, Wilmslow Road, Didsbury, Manchester, M20 2LS UK; 2 Shire, Zählerweg 10, 6301 Zug, Switzerland; 3 Analysis Group, Inc., 111 Huntington Ave, 10th Floor, Boston, MA 02199 USA; 4 RTI Health Solutions, 200 Park Offices Drive, Research Triangle Park, NC 27709 USA

**Keywords:** ADHD, Lisdexamfetamine, Cost-effectiveness analysis, Economic evaluation, Attention-deficit/hyperactivity disorder, Adult, I110

## Abstract

**Background:**

Attention-deficit/hyperactivity disorder (ADHD) is a chronic neurobehavioral disorder in children that may persist into adulthood. Lisdexamfetamine dimesylate (LDX) is approved in many countries for ADHD treatment in children, adolescents, and adults.

**Objectives:**

Estimate the cost-effectiveness of LDX as a first- or second-line treatment for adults with ADHD from the United Kingdom (UK) National Health Service (NHS) perspective compared with methylphenidate extended release (MPH-ER) and atomoxetine (ATX).

**Methods:**

A 1-year decision-analytic model was developed. Health outcomes included response, non-response and inability to tolerate. Efficacy data were obtained from a mixed-treatment comparison (MTC). Response was a score of 1 or 2 on the Clinical Global Impression–Improvement scale. Tolerability was assessed by discontinuation rates due to adverse events. Utilities were identified via a systematic literature review. Health care resource use estimates were obtained via a survey of clinicians. Daily drug costs were estimated from mean doses reported in the trials used in the MTC. One-way and probabilistic sensitivity analyses (PSAs) were performed.

**Results:**

LDX dominated MPH-ER and ATX; reducing mean per-patient annual cost by £5 and £200, and increasing mean quality-adjusted life years (QALYs) by 0.005 and 0.009, respectively. In the PSA, the probability of cost-effectiveness for LDX vs. MPH-ER and ATX at a threshold of £20,000 per QALY was 61% and 80%, respectively.

**Conclusions:**

From the perspective of the UK NHS, LDX is likely to provide a cost-effective treatment for adults with ADHD. This conclusion may be drawn with more certainty in comparison with ATX than with MPH-ER.

**Electronic supplementary material:**

The online version of this article (doi:10.1007/s10198-016-0864-4) contains supplementary material, which is available to authorized users.

## Background

Attention-deficit/hyperactivity disorder (ADHD) is a neurodevelopmental disorder with symptoms of inattention, hyperactivity, and impulsivity that present in multiple settings [1]. ADHD persists into adolescence and adulthood in 50–60% of childhood cases of ADHD [2]. ADHD is estimated to affect 2–5% of the adult population worldwide, depending on country, and choice of ADHD diagnostic criteria [3–6]. Unlike childhood ADHD, gender ratios tend to be fairly equal in studies of adult ADHD [7–9]. Persistent inattentive ADHD symptoms in adulthood are significantly related to an increased risk of long-term work disability [10]. In the United States (US), the economic burden of ADHD in adults is estimated to be from $105 billion to $194 billion per year, of which the largest cost is productivity and income losses, ranging from $87 billion to $138 billion per year [11].

Management of ADHD usually includes psychotherapy, medications, or a combination of both psychotherapy and pharmacotherapy. In the United Kingdom (UK), methylphenidate (MPH), either extended release (ER) or immediate release (IR), is recommended by the National Institute for Health and Care Excellence (NICE) Guidance CG72 to be tried first; if MPH is ineffective or unacceptable, atomoxetine (ATX) or dexamfetamine (DEX) may be tried [12]. ATX or DEX should be considered in adults unresponsive or intolerant following an adequate trial of MPH (usually approximately 6 weeks) [12]. Caution should be exercised when prescribing DEX to those likely to be at risk of stimulant misuse or diversion [12].

Lisdexamfetamine dimesylate (LDX) is a prodrug; following absorption, LDX undergoes hydrolysis to DEX and lysine. LDX has received marketing authorizations for the treatment of ADHD in Australia, Brazil, Canada, Denmark, Germany, Ireland, Israel, Mexico, Norway, Spain, Sweden, Switzerland, the UK, and the US. LDX has been shown to be effective in reducing the symptoms of ADHD in a randomized controlled trial in adults [13], using the ADHD Rating Scale IV (ADHD-RS-IV) [14]. During the open-label phase of a modified analogue classroom study of adults with ADHD, LDX was associated with improvements from baseline in executive function behavior, using the validated, self-reported Brown Attention-Deficit Disorder Scale [15, 16]. In the modified analogue classroom study, LDX treatment demonstrated efficacy in adults with ADHD who had significant impairments in ADHD core symptoms and executive function, as well as efficacy in quality of life as assessed by Adult ADHD Impact Module (AIM-A) [17]. In a 10-week randomized, placebo-controlled trial of LDX in adults with ADHD and clinically significant executive function deficits, LDX improved AIM-A multi-item domain scores versus placebo [18]. A randomized, double-blind, placebo-controlled study using a validated driving simulator paradigm showed that LDX may reduce driving risks in young adults with ADHD [19]. Postmarketing survey data suggest that the rate of non-medical use of LDX is lower than that for short-acting stimulants and lower than or equivalent to long-acting stimulant formulations [20]. In a randomized, double-blind, placebo-controlled, two-way crossover study conducted in a simulated adult workplace environment, LDX significantly improved the Permanent Product Measure of Performance scores versus placebo and maintained improvement throughout the day from the first (2 h) to last (14 h) postdose time points versus placebo in adults with ADHD [21].

The objective of the present study was to estimate the cost-effectiveness of LDX compared with MPH-ER or ATX in the treatment of adults with ADHD. The results of the analysis are presented as the total costs and total quality-adjusted life years (QALYs) for each drug, as well as the incremental costs and QALYs for LDX when compared with MPH-ER or ATX. In addition, the incremental cost-effectiveness ratios (ICERs) for LDX relative to MPH-ER or ATX are presented and evaluated against an established cost-effectiveness threshold of £20,000 per QALY [22]. A comparison of the costs and health outcomes predicted by the model is intended to aid physicians and health care decision makers as they make decisions about efficient use of drugs indicated for adults with ADHD.

## Methods

A decision-tree model was developed in Microsoft Office Professional 2013 (Excel version 15) to evaluate the cost-effectiveness of LDX, compared with MPH-ER or ATX, from the perspective of the UK’s National Health Service (NHS). Drug treatment is recommended for adults with ADHD with either moderate or severe levels of impairment [12]. The selection of comparators was guided by the clinical guidelines published by NICE [12] and validated by a UK-based clinical expert. The ER formulation of MPH was chosen because it is used more commonly in clinical practice in the UK than the IR formulation (Shire data on file; IMS Database, March 2015) and MPH-ER has a broader clinical evidence base, reflected by a larger number of clinical trials in adults (see Online Resource 1). Non-pharmacological interventions were included in the analysis as part of the non-drug costs and were assumed to vary for responders and non-responders to drug therapy. There is no evidence to suggest that any of the drug treatments would result in a reduction in the amount or type of non-pharmacological or behavioral intervention required for those responding to therapy or for those not responding to therapy.

**Fig. 1:**
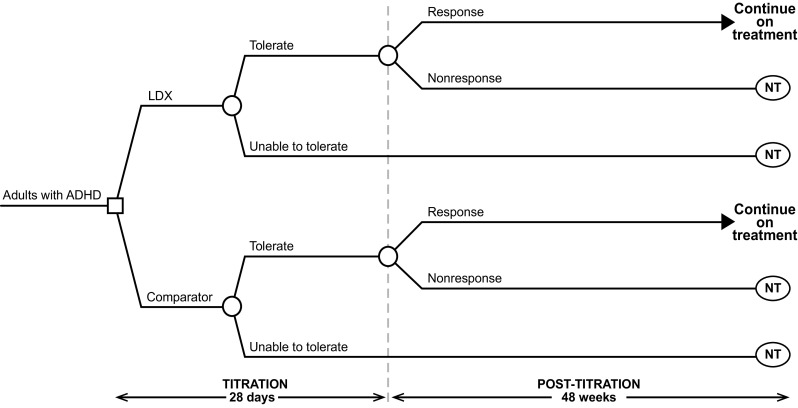
Model Structure. *ADHD* attention-deficit/hyperactivity disorder, *LDX* lisdexamfetamine, *NT* no pharmacological treatment. Reproduced from Zimovetz, E.A., Beard, S.M., Hodgkins, P. et al. CNS Drugs (2016) 30:985. doi:10.1007/s40263-016-0354-3, under the terms of the Creative Commons Attribution-NonCommercial 4.0 International License (http://creativecommons.org/licenses/by-nc/4.0/), with minor amends reflecting the change in study population from children/adolescents to adults.

The target population for the cost-effectiveness analysis was adults with ADHD, which reflects the anticipated therapeutic licensed indication for LDX in both adult continuers (i.e., adults whose ADHD was diagnosed during childhood and adolescence) and de novo adult patients (i.e., adults with ADHD not diagnosed during childhood or adolescence). The health outcomes included were “tolerate”, “unable to tolerate”, “response” and “non-response” (Fig. 1). The impact of using LDX as an alternative to MPH-ER or ATX in terms of costs and health outcomes was estimated in the model based on the number of patients who achieved response to treatment and those who did not, including those who discontinued due to adverse events. Costs and utilities corresponding to the patients’ health states were assigned to each of these patients.

The base-case analysis evaluated direct medical costs and health-related quality of life associated with 1 year of treatment, including the initial 28-day drug titration period. A time horizon of up to 5 years was examined as a sensitivity analysis with an annual discounting rate of 3.5% applied to both costs and benefits. The modeling framework and key assumptions, including the 1-year time horizon, were adapted from the health technology assessment model used in an earlier assessment of ADHD drugs by NICE [23].

The uncertainty in the ICER estimate was explored by one-way sensitivity analysis and probabilistic sensitivity analysis (PSA). Quality control of model programming and verification of all input data with original sources was performed according to a prespecified test plan by health economists who were not involved in the model development. Key model assumptions were assessed for face validity by a UK clinician with extensive experience in treating and research in adults with ADHD, and by an independent health economics expert.

## Model assumptions

The following assumptions were made in the base-case analysis.

Adult continuers or de novo adult patients enter the model when they initiate a course of treatment with LDX, MPH-ER or ATX.

Patients begin titration lasting 28 days, during which the optimal dose of treatment is reached (ATX may require a longer titration period, which is tested in the sensitivity analysis).

Patients who experience intolerable side effects discontinue treatment in the middle of the titration period (i.e., after 14 days on treatment). For patients who discontinue treatment during the titration period, utilities and costs during the titration period (28 days) are represented by a 50%/50% mix of the responder and non-responder utility values, and a 50%/50% mix of the responder and non-responder non-drug costs, respectively. This approach was based on the assumption that, on average across different treatments, patients who discontinue responded half way through the first month (consistent with the assumption by King et al. [23] in the UK Health Technology Appraisal of ADHD drugs in children and adolescents). Alternative assumptions were explored in which these patients were assumed to have the same utility during their titration period as responders and as non-responders.

Patients who discontinue treatment due to intolerable side effects do not initiate additional pharmacological treatment. Those who discontinue, the same as non-responders, are assumed to receive behavioral therapy. This assumption was made largely due to lack of relevant clinical data for follow-up therapies and the fact that in the model, these therapies would be the same in both the LDX and the comparator arms, hence not adding any differentiation to the model results. The patients who discontinue are, therefore, assumed to have the same utilities and costs as non-responders for the remainder of the 1-year model time horizon.

At the end of the titration period, non-responding patients discontinue treatment without initiating any further pharmacological treatment and are assigned the non-response costs and utilities for the titration period and throughout the model’s remaining time horizon.

Patients who respond to treatment at the end of the titration period remain on treatment throughout the remainder of the model’s time horizon, maintaining their level of response.

Patients who responded to and tolerated treatment are assumed to be adherent and persistent on treatment over the time horizon of the model, as was generally observed in the pivotal trials. This assumption is consistent with that made in the health technology assessment model presented by King et al. [23].

This dichotomous response framework was adapted from the model developed by King et al. [23] as part of the UK Health Technology Appraisal of ADHD drugs in children and adolescents.

Clinical input parameters applied in the model were estimated via a Bayesian network meta-analysis (NMA) of trials in adults with ADHD.

Costs and outcomes are not discounted in the base-case analysis, given the time horizon of 1 year.

## Model parameter inputs

### Efficacy and safety

The economic model applied clinical input parameter values estimated via a Bayesian NMA of trials in adults with ADHD. A systematic literature review was conducted to identify clinical evidence for treatments of ADHD. The review was conducted in accordance with a prespecified literature review protocol. The following six medical databases were searched: MEDLINE, Embase, Cochrane library, PsychINFO, CINAHL and Science Citation Index. Grey literature was also searched, including proceedings from relevant conferences. Studies were selected independently by two reviewers, with discrepancies resolved through consensus or consultation with a third reviewer if a consensus could not be reached. The inclusion and exclusion processes were thoroughly documented, including completion of the Preferred Reporting Items for Systematic Reviews and Meta-analyses (PRISMA) diagram (available in Online Resource 1).

The NMA was performed in accordance with recommendations from NICE’s Decision Support Unit and International Society for Pharmacoeconomics and Outcomes Research, and used a mixed-treatment comparison (MTC) framework [24, 25]. A detailed description of the methods used for the MTC is presented in Online Resource 1.

The Clinical Global Impression–Improvement (CGI–I) scale was chosen as the measure of response to treatment based on data reported in most of the clinical trials included in the MTC; clinical response was measured as a score of 1 (much improved) or 2 (improved) on the clinician-rated CGI–I scale.

None of the trials of ATX included in the MTC reported response data defined using the CGI–I scale. Therefore, an imputation analysis was conducted to estimate CGI–I response for ATX based on ADHD-RS-IV total score change from baseline. The imputation analysis was based on methods first presented by Goodman et al. [26], which involved the estimation of the proportion achieving CGI–I response based on the ADHD-RS-IV change from baseline. Using data from trials of LDX that reported both ADHD-RS-IV change from baseline and CGI–I response, quadratic regression was used to model the relationship between these quantities. Then the relationship identified via the quadratic regression was used to identify a cutoff for ADHD-RS-IV change, such that ADHD-RS-IV changes larger than the cutoff corresponded to CGI–I response. The distribution of the ADHD-RS-IV change in the ATX trial was identified using the mean and standard deviation under the assumption of normality. Then the cutoff was applied to this distribution to identify the proportion with CGI–I response. Alternative methods to determine the cutoff were evaluated also, and the imputation method was validated using other trials reporting both ADHD-RS-IV change and CGI–I response. Table 1 presents the Bayesian MTC results with ATX data derived using a quadratic regression imputation analysis (see Online Resource 1).

**Table 1:** Primary base-case analysis: relative risks for treatment response (drug vs. placebo)

Treatment	Relative risk (95% CrI)	Placebo risk (95% CrI)
LDX	2.14 (1.71–2.57)	0.3084 (0.264–0.353)
ATX	1.65 (1.00–2.32)	
MPH-ER	1.84 (1.44–2.23)	

*ADHD-RS-IV* ADHD Rating Scale IV, *ATX* atomoxetine, *CrI* credible interval, *CGI–I* Clinical Global Impression–Improvement, *LDX* lisdexamfetamine dimesylate, *MPH*-*ER* methylphenidate extended release

Response was defined by a rating of 1 or 2 in CGI–I score. Quadratic regression extrapolation method (random effects model, combined doses) was used for the ATX arm, using only the ADHD-RS-IV scores in extrapolating the CGI–I-based response for ATX

Withdrawal rates were based on discontinuations due to adverse events as reported within the trials and were estimated via an MTC. Table 2 presents the Bayesian MTC results for rates of withdrawals due to adverse events.

**Table 2:** Relative risks for discontinuation due to adverse events (drug vs. placebo)

Treatment	Relative risk (95% CrI)	Placebo risk (95% CrI)
LDX	3.21 (0.93–7.90)	0.0443 (0.035–0.053)
ATX	2.67 (1.68–4.13)	
MPH-ER	2.76 (1.83–4.07)	

*ATX* atomoxetine, *CrI* credible interval, *LDX* lisdexamfetamine dimesylate, *MPH*-*ER* methylphenidate extended release

The economic analysis did not incorporate incidences of individual adverse events, nor did it include the corresponding costs and disutilities associated with these events because of similar rates of mild or moderate side effects of the comparators and because more severe adverse events would lead to treatment discontinuation and would be accounted for in the model through discontinuation. A 12-month open-label, single-arm study demonstrated that LDX has a safety profile consistent with long-acting stimulant use [27].

### Health-state utilities

A systematic review of economic literature in ADHD was conducted to identify utility values. The systematic literature review was performed in accordance with a prespecified protocol; searches were conducted via electronic medical databases and specified websites. A detailed description of the systematic review of the economic literature is included in Online Resource 2. The mean utility values used in the economic analysis were 0.76 for responders and 0.68 for non-responders (95% confidence intervals, not reported for either) [28]. These utility values were obtained from a web-based survey using EuroQol 5-Dimensions 3 Levels (EQ-5D-3L) questionnaires completed by adults with ADHD [28]. The rationale for selecting the Mitsi et al. [28] study as the source of the utility values in the model was twofold. First, this study complied with the NICE reference case, which states that measurement of changes in health-related quality of life should be reported directly by patients, the value of changes in patients’ health-related quality of life should be based on public preferences using a choice-based method, and the EQ-5D is the preferred measure of health-related quality of life in adults [22]. Second, the study reported utility values within a dichotomous framework of response and no response, which was appropriate for the health states used in the economic analysis.

### Resource use and costs

The systematic review of economic literature in ADHD highlighted the data gap in published cost and resource use estimates appropriate for inclusion in the current economic analysis. Health care resource utilization estimates associated with response and non-response were obtained from a survey of clinicians treating patients with ADHD in the UK at the time of the study. The sample consisted of 60 specialists, all psychiatrists, with 83% based in England and 17% based in Scotland. The survey methods are presented in Online Resource 3.

Unit costs of health care resources from the National Reference Costs schedules were then applied to these resource utilization estimates to calculate the costs associated with responders and non-responders. The non-drug costs translated to a per-month (28 days) cost of £115.84 for each responder and to £337.82 for each non-responder (Table 3).

**Table 3:** Resource use and costs applied in the base-case analysis

Resource item	Unit cost	Units per year (SD)	Average cost per year
Responders			
Psychiatrist^a^	£266.27	3.34 (2.39)	£889.34
Psychologist^b^	£201.38	1 (NA)	£201.38
GP^c^	£37.00	3.30 (2.43)	£122.10
Nurse^d^	£44.00	3.72 (4.97)	£163.68
Blood pressure^e^	£12.14	2.92 (2.15)	£35.45
Weight measurement^e^	£12.14	2.94 (2.44)	£35.70
Blood test^f^	£3.00	1.40 (1.71)	£4.20
ECG^g^	£52.00	0.92 (1.22)	£47.84
EEG^h^	£72.00	0.08 (0.33)	£5.76
Allergy test^i^	£5.00	0.09 (0.49)	£0.45
**Total (per 28 days)**	–	–	**£1506 (£115.84)**
Non-responders			
Psychiatrist^a^	£266.27	6.83 (4.02)	£1818.62
Psychologist^b^	£201.38	9.67 (5.82)	£1947.34
GP^c^	£37.00	5.83 (3.89)	£215.71
Nurse^d^	£44.00	5.38 (6.13)	£236.72
Blood pressure^e^	£12.14	3.62 (2.44)	£43.95
Weight measurement^e^	£12.14	3.36 (2.54)	£40.80
Blood test^f^	£3.00	1.98 (2.13)	£5.94
ECG^g^	£52.00	1.36 (1.63)	£70.72
EEG^h^	£72.00	0.15 (0.50)	£10.80
Allergy test^i^	£5.00	0.21 (0.66)	£1.05
**Total (per 28 days)**	–	–	**£4392 (£337.82)**

*ECG* electrocardiogram, *EEG* electroencephalogram, *GP* general practitioner, *NA* not applicable, *NHS* National Health Service, *SD* standard deviation

^a^Source: Curtis (2013): unit costs of health and social care 2013 (15.7 Consultant: psychiatric—per face-to-face contact. Excludes cost of qualifications) [29]. Inflated to 2015 prices using the hospital and community health services (HCHS) index [30]

^b^Source: Department of Health (2015): national schedule of reference costs year: 2014–15—all NHS trusts and NHS foundation trusts—outpatient attendances data (656 clinical psychology) [31]

^c^Source: Curtis and Burns (2015): unit costs of health and social care 20,155 (10.8b general practitioner—unit costs. Per-patient contact lasting 11.7 min. Cost excludes cost of qualification) [30]

^d^Source: Curtis and Burns (2015): unit costs of health and social care 2015 [10.4 nurse specialist (community)—unit costs. Per hour. Cost excludes cost of qualification] [30]

^e^Source: Curtis and Burns (2015): unit costs of health and social care 2015 [10.6 nurse (GP practice)—unit costs. Based on £47 per hour and consultation lasting 15.5 min. Cost excludes cost of qualification] [30]

^f^Source: Department of Health (2015): national schedule of reference costs year: 20,145—NHS trusts and NHS foundation trusts: directly accessed: pathology services. DAPS05—Hematology [31]

^g^Source: Department of Health (2015): national schedule of reference costs year: 2014–15—NHS trusts and NHS foundation trusts: direct access: diagnostic services EY51Z—electrocardiogram monitoring and stress testing [31]

^h^Source: Department of Health (2015): national schedule of reference costs—year 2014–15—NHS trusts and NHS foundation trusts: direct access: diagnostic services. AA33C—conventional EEG, EMG or nerve conduction studies with length of stay 2 days or less, 19 years and over [31]

^i^Source: Department of Health (2015): national schedule of reference costs year: 2014–15—NHS trusts and NHS foundation trusts: directly accessed pathology services. DAPS06—immunology [31]

Drug unit costs were obtained from the British National Formulary. The analysis was based on the prices for LDX of £2.08 (for a 30-mg tablet), £2.45 (for a 50-mg tablet) and £2.97 (for a 70-mg tablet); the prices for MPH-ER of £1.04 (for an 18-mg tablet), £1.23 (for a 27-mg tablet) and £1.42 (for a 36-mg tablet); the prices for ATX of £1.90 (for 10-, 18-, 25-, 40-, and 60-mg tablets) and £2.53 (for an 80-mg tablet) [32]. Drug costs were calculated using the weighted average doses and per-milligram drug costs. The average doses were derived from trials used in the MTC to calculate response rates. Each per-milligram cost was based on the cost of a pack with the tablet size closest to the given mean dose.

### Sensitivity analyses

A number of sensitivity analyses were performed to explore the robustness of the economic model. These included a deterministic one-way sensitivity analysis and a PSA.

### One-way sensitivity analysis

In the deterministic one-way (univariate) sensitivity analysis, the stability of the model’s results was tested over a range of input data values, whereby parameters were changed from their base-case values one at a time, with all other parameters remaining constant (Table 4). The following summarizes the variables considered in the one-way sensitivity analysis.

**Table 4:** Univariate sensitivity analysis input parameter estimates

Input parameter	Base-case analysis		Sensitivity analysis	
	Value	Source	Value	Source
Efficacy (lower CrI)	RR for treatment response (drug vs. placebo)	Bayesian NMA	RR for treatment response (lower CrI)	Bayesian NMA
LDX	2.14		1.71	
ATX	1.65		1.00	
MPH-ER	1.84		1.44	
Placebo	0.3084		0.264	
Efficacy (upper CrI)	RR for treatment response (drug vs. placebo)	Bayesian NMA	RR for treatment response (upper CrI)	Bayesian NMA
LDX	2.14		2.57	
ATX	1.65		2.32	
MPH-ER	1.84		2.23	
Placebo	0.3084		0.353	
Safety (mean discontinuation rate −1SD)	Discontinuation rate due to adverse events	Bayesian NMA	Calculated discontinuation rate due to adverse events	Discontinuation rate due to adverse events calculated as mean discontinuation rate −1SD using posterior distributions from the Bayesian MTC
LDX	3.21		1.39	
ATX	2.67		2.04	
MPH-ER	2.76		2.18	
Placebo	0.044		0.04	
Safety (mean discontinuation rate +1SD)	Discontinuation rate due to adverse events	Bayesian NMA	Calculated discontinuation rate due to adverse events	Discontinuation rate due to adverse events calculated as mean discontinuation rate +1SD using posterior distributions from the Bayesian MTC
LDX	3.21		5.02	
ATX	2.67		3.29	
MPH-ER	2.76		3.33	
Placebo	0.044		0.05	
Health-state utility	Responder/non-responder:	Mitsi et al. [28]	Responder/non-responder:	Matza et al. [33]
LDX	0.76/0.68		0.82/0.68	
ATX	0.76/0.68		0.82/0.68	
MPH-ER	0.76/0.68		0.82/0.68	
Resource utilization costs	Responder/non-responder (per 28 days): £115.84/£337.82	Resource use estimates based on survey of UK clinicians; unit costs based on national sources	Responder/non-responder (per 28 days): £139.17/£337.82	Assumption (annual responder costs increased by one additional visit to psychiatrist and one to GP)
Time horizon: all treatments	1 year	Assumption	5 years	Assumption
Drug-costing method^a^	Method A: using mean doses from trials	Average doses estimated using doses reported in trials included in the MTC^b^	Method B: using real-world daily consumption from the IMS database^c^	Assumption
LDX	£70.90		£66.56	
MPH-ER	£56.24		£57.83	
ATX	£71.03		£75.36	
Length of titration period^d^				
LDX	28 days	Assumption	28 days	Assumption
MPH-ER	28 days		28 days	
ATX	28 days		84 days	

*ATX* atomoxetine, *CRI* credible interval, *LDX* lisdexamfetamine dimesylate, *MPH*-*ER* methylphenidate extended release, *MTC* mixed-treatment comparison, *NMA* network meta-analysis, *RR* response rate, *SD* standard deviation, *UK* United Kingdom

^a^Differences in drug costs between Method A and Method B are applicable only to the post titration costs. The same costs for the titration period were used in both methods

^b^Weighted average doses from trials (51.5 mg for LDX, 50.93 mg for MPH-ER and 80 mg for ATX) were multiplied by the costs per milligram of the corresponding drug. Each per-milligram cost was based on the cost of the package with a tablet size closest to a given average dose

^c^Real-world daily UK consumption estimates (1.62 tablets per day for MPH-ER and 1.39 tablets per day for ATX) were derived from the IMS databases [Shire Pharmaceuticals: IMS Midas and IMS Prescription Databases 2013. (2014)]. For LDX, real-life usage was based on an assumption (1 tablet per day)

^d^Variable length of titration period is applicable only to analyses containing ATX. The assumption of 84 days as the length of the titration period for ATX reflects that, in a proportion of ATX patients, response may be achieved gradually over approximately 3 months

#### Efficacy

In the base-case analysis, the model applied mean relative risks of treatment response for each drug versus placebo estimated via a Bayesian NMA of trials in adults with ADHD. The sensitivity analysis applied the lower and upper credible interval values.

#### Safety

In the base-case analysis, to incorporate tolerability, the model applied mean discontinuation rates due to adverse events. The credible intervals for discontinuation rates estimated by the NMA were unusually wide due to a low rate of adverse events observed in the clinical trials. The sensitivity analysis, therefore, used values calculated as the mean discontinuation rate plus (or minus) one standard deviation.

#### Utility values

In the base-case analysis, the model applied health-state utility estimates reported by Mitsi et al. [28]. This study’s methodology was considered most compliant with the NICE reference case; however, the reported utility value for responders might be slightly lower than expected for adults with ADHD who are otherwise healthy. The sensitivity analysis applied alternative published utility values reported by Matza et al. [33], in which the utility value for responders was higher (0.82 vs. 0.76) and the utility value for non-responders was the same as in the study by Mitsi et al. [28].

#### Resource use estimates

In the base-case analysis, the model applied estimated resource use for responders and non-responders derived via a survey of UK-based practicing clinicians. In the sensitivity analysis, the cost for responders was increased by one additional visit to a psychiatrist and one additional visit to a GP (resulting in an increase of ~20% in the monthly non-drug cost for responders).

#### Time horizon

The base-case analysis assumed a time horizon of 1 year, which was extended to 5 years, without changing the model assumptions, in a sensitivity analysis.

#### Drug-costing method

The base-case analysis used a method of drug costing that used the average daily doses from clinical trials in calculating the daily drug costs. The sensitivity analysis explored the effect of applying the drug costs calculated based on real-world drug utilization, rather than on the drug usage reported in the clinical trials.

#### Length of titration period for ATX

Response in the model was assessed at the end of the titration period, which was represented by 28 days. The base-case analysis assumed the same length of the titration period for LDX and ATX. The sensitivity analysis explored the differential time to response that was seen with patients on ATX (i.e., the antidepressant-like response typically seen after 8–12 weeks on treatment).

### Scenario analyses exploring a longer model time horizon and a different non-responder resource use

The stability of the model’s results was tested over a longer time horizon and alternative assumptions about non-responder resource use. Under this analysis, the model time horizon was extended to 5 years and non-responder annual resource use was adjusted to reflect the lower frequency of follow-up expected in the longer term based on possible decline over time of symptoms of ADHD (decreased by one visit to psychiatrist, one visit to psychologist and one visit to GP). This scenario was run twice—using the base-case utility inputs from Mitsi et al. [28] and then using the alternative utility inputs from Matza et al. [33].

### Additional scenario analyses

Alternative assumptions were explored in which patients who discontinue were assumed to have the same utility during their titration period as responders and then as non-responders, rather than a 50%/50% mix of the responder and non-responder utilities assumed in the base-case analysis.

Additional analysis was conducted to compare LDX with MPH-IR. As no relevant clinical trials of MPH-IR were identified for inclusion in the MTC, the analysis was performed assuming the same efficacy for MPH-IR as for MPH-ER. The basis for this assumption was the result of the trial in children, which showed little difference in efficacy between the ER and IR formulations [34]. The analysis was based on the price of MPH-IR of £0.36 per 20-mg tablet [13]. The average dose for the titration period was assumed to be the same as for MPH-ER (36.98 mg per day), and the average dose for the post-titration period (82 mg per day) was taken from a safety trial of MPH-IR in adults with ADHD identified by the systematic review [35].

### Probabilistic sensitivity analysis

Uncertainty in model input parameters was examined in the PSA, wherein all input parameters, apart from drug costs, which were known with certainty, were simultaneously varied using prespecified distributions reflecting the uncertainty about their true values. Five thousand repeated model simulations were performed. Non-drug costs for responders and non-responders were simulated using the Gamma distribution. Parameters for the Gamma distribution were derived using the mean and standard error of the unit of the resource use obtained from the survey. Utility values were simulated using the uniform distribution with a ±10% variation. Clinical parameter values were not varied using prespecified distributions. Instead, these were simulated from the posterior distributions using the mean and standard deviation from 50,000 posterior samples from the Bayesian MTC, assuming a normal distribution based on visual inspection of the data.

## Results

### Base-case analysis results

#### LDX vs. MPH-ER

The results suggested that use of LDX was a dominant strategy compared with MPH-ER [i.e., it was less expensive (−£4.78) and more effective (0.005)]. Total 1-year per-patient costs for LDX and MPH-ER were £3379 and £3384, respectively, and total 1-year QALYs, out of a maximum possible 1, were 0.724 for LDX and 0.718 for MPH-ER (Table 5).

**Table 5:** Base-case analysis results (per patient)

Scenario	Comparisons	Total costs (£)	Total QALYs	Incremental costs (£)	Incremental QALYs	ICER (£ per QALY)	INMB (£)^a^
A	MPH-ER vs	3384	0.718	−4.78	0.005	LDX dominant	109
	LDX	3379	0.724				
B	ATX vs	3579	0.715	−199.93	0.009	LDX dominant	381
	LDX	3379	0.724				

*ATX* atomoxetine, *ICER* incremental cost-effectiveness ratio, *INMB* incremental net monetary benefit, *LDX* lisdexamfetamine dimesylate, *MPH-ER* methylphenidate extended release, *QALY* quality-adjusted life year

^a^At £20,000 per QALY

#### LDX vs. ATX

The results suggested that use of LDX was a dominant strategy compared with ATX [i.e., it was less expensive (−£199.93) and more effective (0.009)]. Total 1-year costs per patient for LDX and ATX were £3379 and £3579, respectively, and total 1-year QALYs, out of a maximum possible of 1, were 0.724 for LDX and 0.715 for ATX (Table 5).

### Sensitivity analysis results

#### One-way sensitivity analysis

**Fig. 2:**
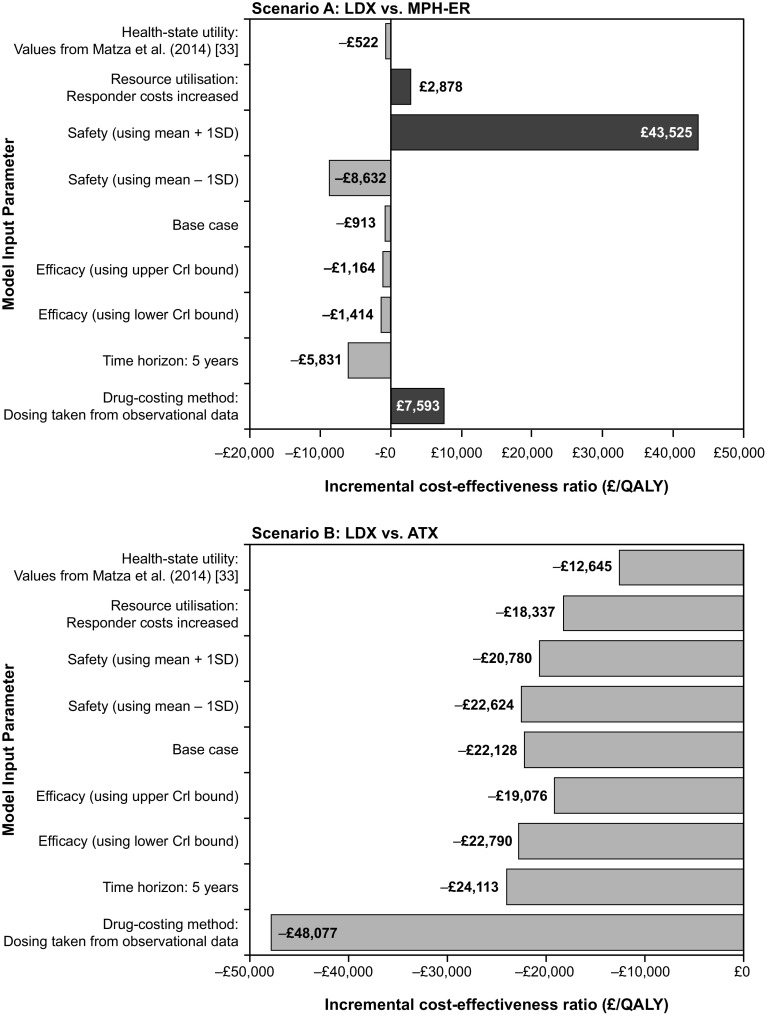
One-way sensitivity analysis results. *ATX* atomoxetine, *LDX* lisdexamfetamine dimesylate, *MPH*-*ER* methylphenidate extended release, *QALY* quality-adjusted life year

Table 6 and Fig. 2 summarize the results of the one-way sensitivity analysis. The results of the model were found to be robust to changes in most of the model input parameter values for both scenarios, apart from changes in discontinuation rates due to adverse events for the MPH-ER comparison. The model was most sensitive to the increase of one standard deviation in the discontinuation rate due to adverse events. For the MPH-ER comparison, such an increase changed the base-case result from LDX being dominant to LDX being not cost-effective (£43,525 per QALY at a threshold of £20,000 per QALY). For the ATX comparison, the base-case result did not change under this scenario, with LDX remaining a dominant strategy.

**Table 6:** Univariate sensitivity analysis results (per patient)

Parameter	Scenario A: LDX vs. MPH-ER	Scenario B: LDX vs. ATX
Base-case results^a^	QALYs: 0.005	QALYs: 0.009
	Costs: −£4.78	Costs: −£199.93
	ICER: Dominant	ICER: Dominant
	INMB^b^: £109	INMB: £381
Efficacy (lower CrI bound)	QALYs: 0.004	QALYs: 0.012
	Costs: −£5.83	Costs: −£270.80
	ICER: Dominant	ICER: Dominant
	INMB: £88	INMB: £508
Efficacy (upper CrI bound)	QALYs: 0.007	QALYs: 0.004
	Costs: −£7.82	Costs: −£82.34
	ICER: Dominant	ICER: Dominant
	INMB: £142	INMB: £169
Safety—rates of discontinuation due to adverse events (mean −1SD)	QALYs: 0.008	QALYs: 0.012
	Costs: −£69.85	Costs: −£271.24
	ICER: Dominant	ICER: Dominant
	INMB: £232	INMB: £511
Safety—rates of discontinuation due to adverse events (mean +1SD)	QALYs: 0.002	QALYs: 0.005
	Costs: £75.24	Costs: −£112.48
	ICER: £43,525 per QALY	ICER: Dominant
	INMB: −£41	INMB: £221
Health-state utility; from Matza et al. [33]	QALYs: 0.009	QALYs: 0.016
	Costs: −£4.78	Costs: −£199.93
	ICER: Dominant	ICER: Dominant
	INMB: £188	INMB: £516
Resource utilization; responder costs increased by one additional visit to psychiatrist and one to GP	QALYs: 0.005	QALYs: 0.009
	Costs: £15.06	Costs: −£165.68
	ICER: £2878 per QALY	ICER: Dominant
	INMB: £90	INMB: £346
Time horizon 5 years	QALYs: 0.025	QALYs: 0.044
	Costs: −£147.12	Costs: −£1050.09
	ICER: Dominant	ICER: Dominant
	INMB: £652	INMB: £1921
Drug-costing method; dosing taken from observational data	QALYs: 0.005	QALYs: 0.009
	Costs: £39.75	Costs: −£434.37
	ICER: £7593 per QALY	ICER: Dominant
	INMB: £65	INMB: £615
Length of titration period; ATX titration period 12 weeks	NA	QALYs: 0.015
		Costs: −£441.92
		ICER: Dominant
		INMB: £733

*ATX* atomoxetine, *CrI* credible interval, *GP* general practitioner, *ICER* incremental cost-effectiveness ratio, *INMB* incremental net monetary benefit, *LDX* lisdexamfetamine dimesylate, *MPH-ER* methylphenidate extended release, *NA* not applicable, *QALY* quality-adjusted life year, *SD* standard deviation

^a^In the base-case analysis, the following values were used for the parameters examined in the sensitivity analysis: utility = 0.76 (responder), 0.68 (non-responder); per-month non-drug costs = £115.84 (responder), £337.82 (non-responder); time horizon = 1 year; per-month drug costs = £70.90 (LDX), £56.24 (MPH-ER), £71.03 (ATX); length of ATX titration period = 4 weeks

^b^The INMB was calculated for the threshold of £20,000 per QALY using the following formula: INMB = incremental QALYs × threshold − incremental cost

For the MPH-ER comparison, the model was also sensitive to changes in drug costs, with base-case result changing from LDX being dominant to LDX being cost-effective at £7593 per QALY, and to changes in the resource use for responders, with base-case result changing from LDX being dominant to LDX being cost-effective at £2878 per QALY, under the willingness-to-pay threshold of £20,000 per QALY. For the ATX comparison, the model was not sensitive to these alternative values.

#### Scenario analyses exploring a longer model time horizon and a different non-responder resource use

For the ATX comparison, the results did not change their direction, and LDX remained a dominant strategy; for the MPH-ER comparison, the results for LDX changed slightly from being dominant to being cost-effective at £477 per QALY (using utility values from the study by Mitsi et al. [28]) and £273 (using utility values from the study by Matza et al. [33]), under the £20,000 per QALY willingness-to-pay (Table 7).

**Table 7:** Scenario analysis results (per patient)

Parameter	Scenario A: LDX vs. MPH-ER	Scenario B: LDX vs. ATX
Scenario analysis^a^, with health-state utility from Mitsi et al. [28]	QALYs: 0.025	QALYs: 0.044
	Costs: £12.04	Costs: −£775.38
	ICER: £477 per QALY	ICER: Dominant
	INMB^b^: £493	INMB: £1646
Scenario analysis^a^, with health-state utility from Matza et al. [33]	QALYs: 0.044	QALYs: 0.076
	Costs: £12.04	Costs: −£775.38
	ICER: £273 per QALY	ICER: Dominant
	INMB: £871	INMB: £2300

*ATX* atomoxetine, *ICER* incremental cost-effectiveness ratio, *INMB* incremental net monetary benefit, *LDX* lisdexamfetamine dimesylate, *MPH-ER* methylphenidate extended release, *QALY* quality-adjusted life year

^a^In the scenario analysis, the following alternative values to the base-case analysis were used: per-month non-drug costs = £115.84 (responder), £299.00 (non-responder); time horizon = 5 years

^b^The INMB was calculated for the threshold of £20,000 per QALY using the following formula: INMB = incremental QALYs × threshold − incremental cost

#### Additional scenario analyses

Alternative assumptions around the utility weight for patients who discontinue during the titration period had very little impact on the ICERs. The mean incremental QALY for the comparison versus MPH-ER changed from 0.0052 to 0.0054 per person, when the responder utility was used in the titration period for those who discontinue. When the nonresponder utility was used, the mean incremental QALY for the same comparison changed from 0.0054 to 0.0050 per person.

The results of the analysis vs. MPH-IR suggested that LDX was cost-effective under the threshold of £20,000 per QALY. The ICER was estimated at £19,362 per QALY, with mean incremental QALYs and costs per person of 0.005 and £101.35, respectively.

#### Probabilistic sensitivity analysis

**Fig. 3:**
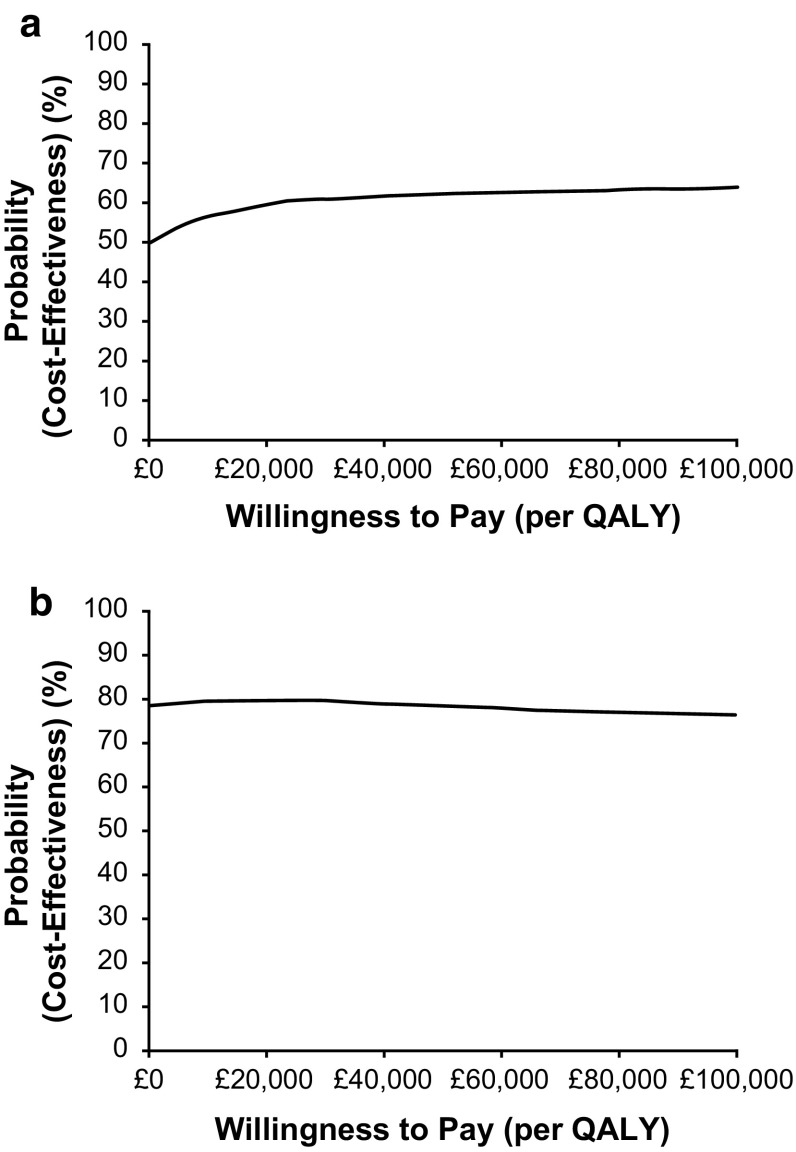
Cost-effectiveness acceptability curves. **a** LDX vs. MPH-ER. **b** LDX vs. ATX. *ATX* atomoxetine, *LDX* lisdexamfetamine dimesylate, *MPH*-*ER* methylphenidate extended release, *QALY* quality-adjusted life year

Table 8 and Fig. 3 present the results of the PSA. For the LDX versus MPH-ER comparison, the results suggested a 61% probability that LDX was cost-effective when compared with MPH-ER at a threshold of £20,000 per QALY. The results suggested that LDX had an 80% probability of being cost-effective against ATX at the willingness-to-pay threshold of £20,000 per QALY.

**Table 8:** Probabilistic sensitivity analysis results (per patient)

Comparisons	Mean total cost (£)	Mean total QALYs	Mean incremental cost (95% CrI) (£)	Mean incremental QALY (95% CrI)	ICER (95% CrI) (£ per QALY)	Probability of cost-effectiveness (%)^a^
LDX vs	3379.34	0.725	–8.14 (−403.88 to 363.51)	0.006 (–0.011 to 0.031)	Dominant (undefined, undefined)^b^	61
MPH-ER	3387.48	0.718				
LDX vs	3377.19	0.723	−195.58 (−675.95 to 269.06)	0.009 (−0.012 to 0.043)	Dominant (undefined, undefined)	80
ATX	3572.77	0.714				

*ATX* atomoxetine, *CrI* credible interval, *ICER* incremental cost-effectiveness ratio, *LDX* lisdexamfetamine dimesylate, *MPH*-*ER* methylphenidate extended release, *QALY* quality-adjusted life year

^a^At £20,000 per QALY

^b^The CrIs for probabilistic ICER estimates are not defined when these estimates are spread over multiple quadrants of the cost-effectiveness plane

## Discussion

Our economic analysis used the results of a Bayesian MTC of efficacy to assess the cost-effectiveness of LDX when compared with MPH-ER or ATX in the treatment of adults with ADHD. The base-case results showed that LDX was a dominant strategy when compared with MPH-ER; however, there was some uncertainty in this result, with the PSA estimating a 61% probability of LDX being cost-effective vs. MPH-ER under a £20,000 willingness-to-pay threshold. One-way sensitivity analysis results revealed that the model, when comparing LDX with MPH-ER, was most sensitive to increase in discontinuation rates due to adverse events. From the MTC outputs, rates of discontinuation due to adverse events had fairly broad credible intervals largely translating from small sample sizes for patients who discontinue in the trials used in the MTC (Table 2 and Online Resource 1). LDX was a dominant strategy when compared with ATX. Sensitivity analyses confirmed the robustness of this result, showing an 80% likelihood of LDX being cost-effective relative to ATX when applying probabilistic methods. One-way sensitivity analysis results revealed no changes in the model results, when comparing LDX with ATX, for any of the variables examined.

The present economic analysis, to the authors’ knowledge, is the first published study on the cost-effectiveness of pharmacotherapy in the adult ADHD population. A recent systematic review published in 2012 identified no published studies on the cost-effectiveness of pharmacotherapy in the adult ADHD population, comparing stimulants, non-stimulants or adjuvant therapy [36].

Our model found that LDX is cost-effective relative to MPH-ER or ATX and would provide a good additional stimulant option. The inclusion of a new cost-effective treatment option for adults with ADHD could allow for patients’ individual needs and preferences to be taken into consideration. Having multiple stimulant and non-stimulant treatment options available is likely to contribute to a reduction in the overall burden of ADHD.

The choice of a short time frame for this analysis was driven by the lack of long-term data needed for a longer model time horizon. The chosen time frame requires minimal extrapolation of the short-term data from the trials (duration of trials used in the MTC ranged from 4 to 34 weeks), thus minimizing the uncertainty. The shorter time horizon can also be justified by the current clinical guidelines, which recommend that treatment for adults with ADHD should be reviewed at least annually [12]. The one-way sensitivity analysis and the scenario analyses showed that extending the model time horizon to 5 years and changing non-response resource use did not have much impact on the cost-effectiveness results.

This modeled assessment had some limitations. First, the MTC was limited by the number of prior studies, which led to the need for an imputation analysis and limited the precision of the estimates. Although the imputation analysis was used to produce conservative point estimates of the relative risk of CGI–I response, the estimates of variability from that analysis do not account for the additional variability due to the imputation. Hence, although the point estimates are likely to be conservative, the corresponding credible intervals and confidence intervals should be interpreted with caution. Generally, indirect treatment comparisons enable us to compare treatments not otherwise compared in a head-to-head clinical trial, but they do not equate to the same level of evidence as direct (head-to-head) comparisons. The results of the current MTC were generally consistent with the results of the pivotal trials, and no statistical evidence of heterogeneity across trials was found. Second, due to lack of data, the study did not consider any treatment for non-responders and assumed these patients discontinued drug therapy. In real-world clinical practice, such patients may receive one of the other comparators or off-label medications (e.g. bupropion, clonidine, modafinil or imipramine) or combination treatments [12]. Neither did it consider dose reduction if side effects became troublesome. Third, the utility data for intolerable side effects were not based on disutility data of individual side effects leading to discontinuation, but were estimated in the model as a 50%/50% mix of the responder and non-responder utility values. However, alternative assumptions around the utility weight for patients who discontinue during the titration period had very little impact on the ICERs. Fourth, the model did not consider real-life medication compliance, again, due to the lack of data, this time on the relationship between adherence to therapy and symptom reduction. Finally, the study was conducted from the UK NHS perspective and did not include the broader societal perspective, which is an important cost driver in the overall cost burden of ADHD. Taking into account costs associated with the societal perspective likely would result in an even lower ICER for LDX, given a potential cost-offset.

There is limited evidence on the cost-effectiveness of medication to treat ADHD in adults, as well as long-term cost-effectiveness of pharmacotherapies in ADHD. To better inform payers about the economic value of existing medications, future studies should consider identifying subgroups that may have heterogeneous responses to different treatments and expanding the time horizon to incorporate long-term outcomes [36].

## Conclusions

This study suggests that LDX is likely to dominate both MPH-ER and ATX as a therapy for adult patients with ADHD (both previously treated and untreated patients); i.e., total costs are expected to be lower and outcomes (QALYs) are expected to be improved with LDX therapy compared with both MPH-ER and ATX therapy. These results, particularly for the comparison versus MPH-ER, must be seen in light of some uncertainty detected by the PSA. The presented model adds to the health economic information available for policymakers and to general considerations in economic modeling.

## Electronic supplementary material

Below is the link to the electronic supplementary material.

Supplementary material 1 (DOCX 282 kb)

Supplementary material 2 (DOCX 553 kb)

Supplementary material 3 (DOC 856 kb)
