# Supplementary material for: A cost-effectiveness analysis of lisdexamfetamine dimesylate in the treatment of adults with attention-deficit/hyperactivity disorder in the UK
Source: Eur J Health Econ. 2017 Jan 16;19(1):21–35. doi: 10.1007/s10198-016-0864-4 (PMC5773633; doi:10.1007/s10198-016-0864-4)
Supplement: Supplementary file 1 — Supplementary material 1 (DOCX 282 kb) [file 10198_2016_864_MOESM1_ESM.docx]

**Supplementary Material: Network meta-analysis of lisdexamphetamine and other medications for the treatment of adult ADHD**

**February 2015**

Article title: A cost-effectiveness analysis of lisdexamfetamine dimesylate in the treatment of adults with attention-deficit/hyperactivity disorder in the United Kingdom

Journal: The European Journal of Health Economics

Authors: Evelina A Zimovetz, Alain Joseph, Rajeev Ayyagari, Josephine A Mauskopf

Corresponding author: Evelina Zimovetz, RTI Health Solutions; ezimovetz@rti.org

# Background and objectives

Attention deficit hyperactivity disorder (ADHD) is estimated to affect 2% to 5% of the adult population worldwide, depending on country, and choice of ADHD diagnostic criteria (Fayyad et al., 2007; Simon et al., 2009; De Graaf et al., 2008). Although the majority of ADHD cases are diagnosed during childhood, ADHD persists into adulthood for approximately two-thirds of adolescent ADHD patients (Faraone et al., 2006; Klein et al., 2012). If left untreated, ADHD may pose a significant barrier to personal development and cause a substantial psychological and financial burden to patients’ families (Matza et al., 2005). Despite the negative consequences associated with ADHD, the disease was essentially unrecognized, especially in adults, until a few years ago. Although awareness of adult ADHD is on the rise in European countries, under-diagnosis and misdiagnosis are still quite common (Feifel et al., 2008). In addition, management of these patients is often poorly understood.

Stimulants, including amphetamines (AMPH) and methylphenidate (MPH), are effective first-line pharmacological treatment options for the majority of ADHD children and adolescents, with about 70% of patients responding to treatment in the short-term (Olfson et al., 2004). However, neither AMPH nor MPH is approved for adults with ADHD in European countries without restrictions (Retz et al., 2011). Concerta®, an osmotic release oral system MPH (OROS-MPH) compound, is approved for adults who started the drug and achieved good response before the age of 18 years (“adult continuers”). Medikinet Adult, another extended release MPH (MPH-ER) compound, is approved in Germany for adults who were diagnosed when they were children or adolescent and did not have sufficient response to their prior therapy (“adult non-responders”) (Medice, 2011). Beside stimulants, non-stimulant is another treatment option for ADHD patients. Atomoxetine (ATX), a non-stimulant, has recently been approved by the Medicines and Healthcare Products Regulatory Agency (MHRA) in the UK for adult ADHD patients with pre-existing symptoms during childhood (McKee, 2013).^11^ Currently, the UK National Institute for Health and Care Excellence (NICE) clinical guidelines and European Consensus Statement on Diagnosis and Treatment of ADHD recommend MPH as the first-line ADHD therapy among adults (Retz et al., 2011; Kooij et al., 2010; NICE, 2009). If MPH is ineffective or poorly tolerated, ATX can be used.

Lisdexamphetamine (LDX) is a stimulant approved for children, adolescents and adult ADHD patients in the US, Canada under the brand name Vyvanse, for children in Brazil under the name Venvanse, and for children and adolescents in Europe under the brand name Elvanse. A pharmacologically inactive prodrug, LDX is rapidly absorbed from the gastrointestinal tract after oral administration and hydrolysed primarily by red blood cells to dexamfetamine, which is responsible for the drug’s activity. Shire is planning to pursue the adult indication for LDX in the European market. Two phase III clinical trials have demonstrated significantly higher efficacy of LDX compared to placebo in treatment of adult patients with ADHD (Adler et al., 2008; Wigal et al., 2010). Upon entering the market, LDX will be competing with MPH and ATX; however, no systematic comparison has been conducted between LDX and these comparator drugs.

The goal of this study is to fill this evidence gap. Specifically, the study has two aims:

Systematic literature review

Study objective 1: To systematically compile and summarize all available clinical evidence for efficacy of LDX and other relevant comparator drugs with clinical trials in the adult ADHD population

Mixed treatment comparison

Study objective 2: To compare the efficacy and safety of LDX vs. other comparator treatments in adult ADHD using all available clinical evidence, both indirect and direct, using mixed treatment comparisons methodology

# Systematic literature review

A systematic literature review was conducted to identify clinical evidence for the efficacy and safety of treatments for adult ADHD. The databases and corresponding search strategies were selected according to NICE guidelines (NICE, 2012).

## Literature search strategy

Prior to initiating the systematic literature review, a comprehensive search strategy was developed. The interventions of interest for this study included LDX and other drugs that are approved for the treatment of adult ADHD in the UK. Systematic filters for population, age, and study design were constructed and applied to limit results to randomized controlled trials, non-randomized controlled trials, and systematic reviews concerning the pharmacologic treatment of adults with ADHD. Search filters and keywords were constructed according to NICE guidelines (NICE, 2012). The search terms comprised both keyword terminology and database-specific headings, such as Emtree terms, specific to EMBASE, or Medical Subject Headings (MeSH), specific to MEDLINE, CINAHL, and The Cochrane Libraries. The searches were conducted in the first week of September 2013 and covered articles published through the end of August 2013. Full listings of search dates and search terms for each database are available on request.

The following databases were searched, using the interface indicated in brackets:

- Published research
  - MEDLINE and MEDLINE In-Process [OvidSP]
  - EMBASE [OvidSP]
  - PsychINFO [OvidSP]
  - Cochrane Central Register of Controlled Trials (CENTRAL) (Cochrane Library) [OvidSP]
  - CINAHL [EBSCO]
  - Science Citation Index (SCI) [Web of Knowledge]
- Guidelines, Systematic Reviews, and HTAs
  - Cochrane Database of Systematic Reviews (CDSR) [OvidSP]
  - Database of Abstracts of Reviews of Effects (DARE, via Cochrane Library) [OvidSP]
  - Health Technology Assessment Database (HTA, via Cochrane Library) [OvidSP]
  - National Institute for Health and Care Excellence (NICE) [www.nice.org.uk/]
- Completed and ongoing trials
  - Clinicaltrials.gov [clinicaltrials.gov]
  - ISRCTN Register of Clinical Trials [http://wwww.controlled-trials.com/isrctn/]
  - UK Clinical Research Network Study Portfolio (UKCRN) [<http://public.ukcrn.org.uk/search/>]
  - National Research Register (NRR) Archive [<http://www.nihr.ac.uk/Pages/NRRArchive.aspx>]
  - International Clinical Trials Registry Platform (ICTRP) [<http://apps.who.int/trialsearch/>]
- Conference proceedings
  - American Psychiatric Association (APA) Conference Proceedings (2008-2013)[PDFs of APA Conference Abstracts, available at http://www.psychiatry.org]
  - Conference Proceedings Citation Index (CPSI) [Web of Knowledge]

## Selection Criteria

Drugs approved for the treatment of adult ADHD in the UK include LDX, ATX, and MPH. MPH included both MPH immediate release (MPH-IR) and MPH extended release (MPH-ER, composed of MPH intermediate release [MPH-intR] with effects lasting 8-10 hours and the generally preferred extended release [OROS/ER] with effects lasting 12 hours).

Screening of the search results was conducted according to pre-determined inclusion and exclusion criteria designed to identify randomized controlled trials for the safety and efficacy of the drugs of interest. Screening was conducted in three rounds: first, at the level of the title and/or abstract; second, at the level of the full text article; third, according to criteria specific to RCTs. Studies meeting all the inclusion criteria at each level of screening were eligible for the next level of screening. Studies were selected independently by two reviewers, with discrepancies resolved through consensus, or consultation with a third reviewer if a consensus could not be reached.

To be eligible for inclusion in efficacy analyses, an RCT had to report at least one of the efficacy outcomes listed in **Table 1**.

**Table 1. Outcomes of Interest**

| **Outcome** |
| --- |
| Adult ADHD Rating Scale-IV (ADHD-RS-IV) |
| Adult Attention-Deficit/Hyperactivity Disorder Investigator Symptom Rating Scale (AISRS) |
| Clinical Global Impression – Improvement (CGI-I) |
| Clinical Global Impression – Severity (CGI-S) |
| Conners' Adult ADHD Rating Scale, investigator-rated, screening version (CAARS-Inv:SV) |
| Conners' Adult ADHD Rating Scale, observer ratings, screening version (CAARS-O:SV) |
| Conners' Adult ADHD Rating Scale, self-report, screening version (CAARS-S:SV) |
| Barkley Adult ADHD Rating Scale (BAARS) |
| Brown Attention-Deficit Disorder Scale for Adults (BADDS) |
| Global Assessment of Functioning Scale (GAF) |

RCTs including all-cause discontinuation rates and/or discontinuation due to adverse events were considered for inclusion in the safety analysis, independent of the efficacy criteria above. In addition, RCTs need to be comparable to LDX either directly or indirectly (i.e., through placebo).

The full set of exclusion criteria, per round of screening, is listed in **Table 2.** The inclusion and exclusion processes were thoroughly documented, including completion of the Preferred Reporting Items for Systematic Reviews and Meta-analyses (PRISMA) diagram. The corresponding PRISMA diagram may be found in **Figure 1** in **Section 5.1.**

**Table 2. Exclusion criteria by screening round**

| **Reasons for exclusion – Screening Round 1, Title/Abstract** | | | | | | | | | |  |  |  |
| --- | --- | --- | --- | --- | --- | --- | --- | --- | --- | --- | --- | --- |
| 1 | Population not of interest | | | | | | | | | | | |
|  | - | Non-adults (patients aged < 18) are included in the study population | | | | | | | | | | |
|  | - | Not ADHD | |  | | | |  |  |  |  |  |
|  | - | Non-human studies | | | | |  |  |  |  |  |  |
| 2 | Does not include a study drug (MPH, AMPH, ATX, LDX) | | | | | | | | |  |  |  |
| 3 | Inappropriate study design: | | | | |  | |  |  |  |  |  |
|  | - | Preclinical studies | | | |  | |  |  |  |  |  |
|  | - | Phase I studies | | | |  | |  |  |  |  |  |
|  | - | Prognostic studies | | | |  | |  |  |  |  |  |
|  | - | Retrospective studies | | | | | |  |  |  |  |  |
|  | - | Case reports | |  | | | |  |  |  |  |  |
|  | - | Commentaries, letters, and audio files (publication type) | | | | | | | | |  |  |
|  | - | Consensus reports | | |  | | |  |  |  |  |  |
|  | - | Titles without corresponding abstracts | | | | | | |  |  |  |  |
|  | - | Nonsystematic reviews | | | | | |  |  |  |  |  |
| 4 | Outcomes not of interest (e.g., title/abstract specifies that it is a study in pharmacokinetics, biochemical, neuroimaging, genetic studies) | | | | | | | | | | | |
| **Reasons for exclusion – Screening Round 2, Full Text/Full Record** | | | | | | | | | | |  |  |
| 1-3 | Same as Screening Round 1, described above | | | | | | | |  |  |  |  |
| 4 | Inappropriate study design: non-randomized studies, open label extensions of randomized controlled trials, systematic reviews, post-hoc analyses, pooled analyses, or meta-analyses | | | | | | | | | | | |
| 5 | Outcomes not of interest (excluded studies that did not report all-cause discontinuation, adverse events discontinuation, or at least one of the following efficacy measures commonly used in adult ADHD: CAARS, ADHD-RS-IV, AISRS, BAARS, BADDS, CGI scales, or GAF) | | | | | | | | | | | |
| 6 | Full text for the study unobtainable, and with the abstract and/or abbreviated text reporting incomplete information | | | | | | | | | | | |
| 7 | Full text is not in English | | |  | | | |  |  |  |  |  |
|  |  |  |  |  | | | |  |  |  |  |  |
| **Reasons for exclusion – Screening of RCTs^1^** | | | | | | | | |  |  |  |  |
| 1 | Duplicate publication or non-primary publication for trial | | | | | | | | |  |  |  |
| 2 | Trial population has ADHD + a comorbid condition, e.g., substance abuse | | | | | | | | | |  |  |
| 3 | Trial study length is ≤ 3 weeks or ≥ 6 months | | | | | | | |  |  |  |  |
| 4 | Study drug is amphetamine (AMPH), dexamphetamine (d-AMPH), or MPH Modified Release Long Acting (MPH-LA), all arms were given the same study drug (e.g., comparison of dosing schedules), or treatments in the RCT were not connected to LDX in a network either directly or indirectly through common comparators | | | | | | | | | | | |
| 5 | Study is a crossover trial without interim results | | | | | | | |  |  |  |  |
| 6 | Low sample size (N ≤15 for any arm) | | | | | | |  |  |  |  |  |
|  |  |  |  |  | | | |  |  |  |  |  |
|  |  |  |  |  | | | |  |  |  |  |  |
| **Note:** | |  |  |  | | | |  |  |  |  |  |
| [1] | Screening criteria for core analyses are shown. | | | | | | | |  |  |  |  |

## Data Extraction

After the articles were selected, two reviewers independently extracted and tabulated the information using templates designed *a priori*. Any discrepancies in data extracted between reviewers were discussed or consulted with a third reviewer to reach consensus. The following information was extracted from each selected article:

- Bibliographic information: author name, year published, sponsor, title, objective and primary study citation
- Characteristics of selected trial: study design, interventions and comparators, study drug dosage, timing of endpoints, primary and secondary outcomes
- Inclusion and exclusion criteria
- Patient baseline demographics and characteristics by randomized dosing arms and/or for all patients: sample size, age, gender, race, baseline ADHD-RS-IV total scores, and comorbidities
- Reported outcomes of interest, as listed in **Table 1**

The extracted information was primarily based on the published article or, in the absence of a published article, a peer-reviewed abstract, poster, or other record containing complete and adequate information.

## Identification of interventions and outcomes to be included in the MTC

### Selection of final set of interventions for meta-analysis

The interventions determined to be feasible and most pertinent for the meta-analysis were LDX, ATX, MPH-IR, and MPH-ER/OROS. The following treatments were excluded from the final analysis for the reasons listed below:

- Amphetamine: not approved in the UK for the treatment of ADHD in adults
- Dexamphetamine: not approved in the UK for the treatment of ADHD in adults
- MPH-LA: classified as neither MPH-ER nor MPH-IR

In many cases, studies did not specify the form of MPH (IR or ER) administered. As a proxy for the definitions of intermediate/extended release and immediate release MPH, MPH treatment was classified as MPH-IR during the screening if the drug was administered greater than two times a day; otherwise, the treatment was classified as MPH-ER. All osmotic release oral system MPH (MPH-OROS) studies were classified as MPH-ER.

### Selection of end-points for meta-analysis

The outcomes chosen were those for which treatments could be compared against LDX in a MTC. To be included, an outcome had to be reported in an LDX trial, and had to be available in trials of other drugs that formed a connected network with LDX via any common comparators (i.e., active or placebo). In addition, the outcome had to be reported in a form that was usable in the analysis and comparable across trials. In the case of continuous outcomes, availability of standard errors (SEs), either directly or through calculations using 95% confidence intervals, p-values, or standard deviations reported in the publication, was required in each arm. For dichotomous outcomes, the number of patients achieving the outcome, as well as the total sample size, was required in each arm.

**Included outcomes**:

- Attention Deficit Hyperactivity Disorder Rating Scale, Version IV (ADHD-RS-IV): Continuous efficacy assessed as the raw change in ADHD-RS-IV score from baseline to endpoint
- Adult Attention-Deficit/Hyperactivity Disorder Investigator Symptom Rating Scale (AISRS): Continuous efficacy assessed as the raw change in AISRS score from baseline to endpoint
- Clinical Global Impressions scales for improvement (CGI-I): Dichotomous efficacy measure defined by a CGI-I score of 1 or 2
- Safety outcomes:
  - All-cause discontinuation
  - Discontinuation due to adverse events (AEs)

### ADHD-RS-IV

Attention Deficit Hyperactivity Disorder Rating Scale, Version IV (ADHD-RS-IV; Brent et al., 2003) is a questionnaire used for diagnosing ADHD and assessing treatment response. The questionnaire consists of 18 questions, each having four possible responses on severity scale, from none (0) to severe (3). The ADHD-RS-IV score is the sum of the responses and ranges from 0 to 54, where a higher score indicates more severe ADHD. The outcome included in this study was the change in ADHD-RS-IV total score from baseline to trial endpoint.

### AISRS

Adult Attention-Deficit/Hyperactivity Disorder Investigator Symptom Rating Scale (AISRS) measures ADHD symptoms in adults (Goodman, 2009; Spencer et al., 2009). Similar to ADHD-RS (with adult prompts), the AISRS questionnaire consists of 18 questions based on DSM-IV diagnostic criteria. Each symptom is rated using four possible severity scale responses, from none (0) to severe (3). “The AISRS also improves on certain aspects of the ADHD-RS, such as providing a context basis to questions about symptoms and replacing questions that assess two symptom domains with questions that assess only one domain” (Goodman, 2009). AISRS was not used as a separate outcome in this study, but was used in place of ADHD-RS-IV for studies in which the ADHD-RS-IV total score was not reported in one of the analyses.

Note that no studies with an MPH-IR arm reported ADHD-RS-IV or AISRS change so MPH-IR was not included in the ADHD-RS-IV and ADHD-RS-IV/AISRS networks.

### CGI-I

The Clinical Global Impressions scales for improvement (CGI-I) is a 7-point scale used to assess how much the patient’s condition has improved or worsened relative to the patient’s baseline state. The possible ratings are very much improved (1), much improved (2), minimally improved (3), no change (4), minimally worse (5), much worse (6), and very much worse (7). The dichotomous outcome, CGI response, was defined as achievement of a CGI-I score of 1 (very much improved) or 2 (much improved).

### All-cause discontinuation

All-cause discontinuation encompassed patients withdrawing from the study for any reason. Common reasons include adverse events and lack of efficacy.

### Discontinuation due to adverse events

The number of patients discontinuing due to adverse events as determined by the study investigators was also included, and reflects the tolerability of the treatment. Adverse events causing discontinuation in studies used in the MTC included headache, insomnia, anxiety, irritability, and fatigue.

## Identification of interventions and outcomes to be included in the MTC

Studies included in the network meta-analysis are listed in Appendix A. Appendix A also includes evidence table presenting rates of discontinuation due to adverse events reported in the identified trials.

# Mixed Treatment Comparison Methodology

For each outcome identified, a network diagram was created, showing the sources of evidence available for the analysis. An MTC model was fitted for each feasible efficacy outcome measure. Trials reporting the specific outcomes with either direct or indirect links to LDX were included in the respective analyses.

## Bayesian Approach

In accordance with the most recent guidance from the NICE Decision Support Unit (DSU) and International Society for Pharmacoeconomics and Outcomes Research (ISPOR) good practices for indirect comparison, the Bayesian approach to MTC was used (NICE, 2014; Jansen et al., 2011). Both fixed and random effects models were fitted in order to take into account different assumptions regarding heterogeneity of treatment effects across studies (fixed effects models assume a common treatment effect for a given treatment across studies; random effects models assume that the treatment effects are not necessarily the same but are exchangeable in the sense that, while the actual treatment effect may vary across trials, there are no structural differences between the trials). All of the analyses were implemented using the statistical software R and OpenBUGS and JAGS (software packages for implementing Markov Chain Monte Carlo (MCMC) techniques). Non-informative prior distributions were used as parameters in the MTC.

An estimate of treatment effect comparing each treatment to a common comparator, in this case placebo, was generated using the MTC. For continuous outcomes, a normal likelihood model with linear link was employed, and the difference from placebo in mean baseline-to-endpoint change in score was reported. For categorical outcomes, a binomial model likelihood model with logit link was employed, and the odds ratio and relative risk were reported. The placebo risk was computed by pooling the counts in all the placebo arms in the data. The placebo risk uncertainty was measured using the 95% confidence interval around this pooled placebo risk. The odds ratio was converted to relative risk scale using the pooled placebo rate. Fifty thousand iterations were run in the Bayesian MTC to generate the posterior distributions for the treatment effects for each outcome. The uncertainty of the estimates of treatment effect was summarized using 95% Bayesian credible intervals (CrI) based on these posterior distributions. The endpoints of the 95% CrI correspond with the 2.5 and 97.5 percentiles of the posterior distribution of the treatment effect, and correspond to a region which can be claimed to include the treatment effect with a high confidence. In addition, the probability that each drug had the highest efficacy among all treatments was estimated based on the posterior distributions. LDX was also compared individually to each of the other drugs, and the probability that LDX had higher efficacy or lower discontinuation rate in comparison to each of the other drugs was reported.

OpenBUGS and JAGS code from the NICE DSU was used to perform the Bayesian analysis. The code used can be provided on request.

## Frequentist Approach

While “vague priors” or non-informative priors were used in the Bayesian analysis, these selection of the parameters of such priors is not data driven. As a sensitivity analysis, frequentist MTCs were conducted to compare the same set of efficacy and safety outcomes as in the Bayesian MTC. For continuous outcomes, a normal likelihood model with linear link was employed, and the difference from placebo in mean baseline-to-endpoint change in score with confidence interval (CI) was reported. For categorical outcomes, a binomial model likelihood model with log link was employed, and the relative risk with confidence interval was reported. Both fixed and random effects models were fitted.

The frequentist analyses were performed in SAS. The code used to implement the frequentist analysis can be provided on request.

## Heterogeneity and Inconsistency Assessments

Heterogeneity in the evidence network for the efficacy analyses was assessed using methods suggested in the NICE guidelines on evidence synthesis. The heterogeneity of outcomes in studies of LDX vs. placebo that were included in the core efficacy analyses was assessed using the I2 and Cochran’s Q statistics. The heterogeneity across the entire network was also assessed by comparing the Deviance Information Criterion (DIC) for the fixed and random effects models.

The Q statistic tests for the presence of heterogeneity, while I^2^ measures the degree of heterogeneity and represents the proportion of total variability among the effect sizes from different trials that is not explained by sampling error. For example, I^2^ = 0 implies that all variability in effect size estimates is entirely due to sampling error within studies. I^2^ = 50 means that half of the total variability among effect sizes is caused not by sampling error, but by true heterogeneity between studies. In addition, a better fit (lower DIC) for the fixed effects model would indicate that between-study heterogeneity in the effect size is low across the entire network.

Inconsistency, characterized as differences between direct and indirect estimates of treatment contrasts in a network meta-analysis, can be detected by a conflict between “direct” evidence on a comparison between treatments (i.e., evidence from a single randomized study), and “indirect” evidence (i.e., evidence which synthesizes the results of more than one trial). Inconsistency was assessed by comparing such direct and indirect estimates.

## Core Analyses and Sensitivity Analyses

The core Bayesian analysis for all four outcomes had the following characteristics for ADHD-RS-IV score change:

- Random effects model
- One treatment arm per drug (combined doses)

Five sensitivity analyses were done for ADHD-RS-IV score change. Sensitivity analyses were performed to assess different methods of accounting for heterogeneity. The fixed effects model assumes no heterogeneity in outcomes (while the random effects model used in the core analyses assumes the presence of heterogeneity), and thus was chosen to be a part of the sensitivity analyses. Sensitivity analyses were also performed using different ways of identifying treatment (separating by dose), and including or excluding studies that appeared to introduce heterogeneity in the study population. The following highlights the difference between each sensitivity analysis compared to the core analysis:

- Sensitivity 1: Fixed effects model, combined doses
- Sensitivity 2: Random effects model, combined doses, adding in the data from the publication from Spencer (1998) for ATX. Spencer (1998) was excluded from the primary analysis due to being a cross-over study in which it was not clear whether the reported results were from before cross-over.
- Sensitivity 3: Random effects model, treatment arms separated by dose
- Sensitivity 4: Random effects model, combined doses, excluding NCT00937040 for MPH-ER NCT00937040 was inconsistent with other studies in that ADHD-RS-IV was rated by significant others.
- Sensitivity 5: Random effects model, combined doses, excluding the data from the publication from Biederman (2012) for LDX. Biederman (2012) was the only study in the ADHD-RS-IV change network consisting of only young adults (all participants ≤ 30 years old).

The following sensitivity analysis was conducted for ADHD-RS-IV/AISRS change:

- Sensitivity 1: Fixed effects model, combined doses*.*

One sensitivity analysis was done for CGI-I response. Compared to the core analysis, a fixed effects model was used instead of random effects.

- Sensitivity 1: Fixed effects model, combined doses

Two sensitivity analyses each were done for all-cause discontinuation and discontinuation due to adverse events. The following highlights the difference between each sensitivity analysis compared to the core analysis:

- Sensitivity 1: Fixed effects model, combined doses
- Sensitivity 2: Random effects model, combined doses, excluding the data from the publication from Biederman (2012) for LDX and Durell (2013) for ATX. These were the only two studies in the discontinuation networks consisting of only young adults (all participants ≤ 30 years old).

# Imputation Analysis Methodology

## Motivation for Imputation Analysis

In regulatory submissions, it is necessary to submit a cost-effectiveness analysis which is based in part on relative effectiveness measures. This led to a need for a relative effectiveness measure based on a dichotomous outcome. In the current setting, no dichotomous outcomes were directly comparable between LDX and ATX. Of the dichotomous outcomes that were reported in some of the trials identified in the systematic literature review, CGI-I was the best populated, although no article was found that met the selection criteria and reported CGI-I response for ATX. Therefore, in order to be able to perform a comparison of CGI-I between ATX and LDX, an analysis was conducted to link CGI-I response with ADHD-RS-IV change from baseline. This link was then used to impute the percent CGI-I response from the reported ADHD-RS-IV change in each arm of Weisler (2012), the only study with ADHD-RS-IV change for ATX which could be used in MTC.

## Extrapolation using Goodman and direct methods

The imputation analysis was done using two methods: the Goodman method (Goodman, 2010) using linear or quadratic regression, and a direct estimation method. Both methods involved the identification of an ADHD-RS-IV change cutoff corresponding to CGI-I response, using the observation that a decrease in ADHD-RS-IV score generally corresponds to an improvement in CGI-I, and are described in detail below. All arms of the individual patient data from the SPD489-303 trial (corresponding to the Adler (2008) paper), which reported both ADHD-RS-IV change from baseline and CGI-I, were used to derive the cutoff. The cutoff for ADHD-RS-IV change was chosen such that ADHD-RS-IV changes larger than the cutoff corresponded to CGI-I response. All arms were pooled under the assumptions that the ADHD-RS-IV cutoff corresponding to CGI-I response should be uniform regardless of treatment, and that the pooled ADHD-RS-IV score changes are normally distributed. The distribution of the ADHD-RS-IV change in Weisler (2012) was identified using the mean and standard deviation under the assumption of normality. The cutoff was then applied to this distribution to identify the proportion with CGI-I response.

The normality of the ADHD-RS-IV score changes in the pooled arms from the SPD489-303 trial was verified visually using a histogram and a Q-Q plot. In addition, normality was numerically assessed by comparing the cumulative distribution function (CDF) of the pooled data was compared to the CDF of a normal distribution with the same mean and standard deviation as the pooled data.

In the Goodman method, linear and quadratic regression models were fit with CGI-I as the outcome and ADHD-RS-IV change from baseline as the explanatory variable (similar in principle to analyses reported in Goodman, 2010), and a cutoff of CGI-I ≤ 2.5 was used to find the corresponding cut-off of ADHD-RS-IV change on the regression curve. The choice between linear and quadratic models was driven by model fit (visually as well as using the R^2^ statistic) and the intention to prefer the model that overestimated the proportion with CGI-I response, as this provides a conservative estimate of this proportion in the ATX trial of Weisler (2012).

In the direct estimation method, the cutoff of ADHD-RS-IV change was assigned to be the value that gave the percentage of CGI-I response corresponding to what was actually observed in the SPD489-303 trial. In both methods, a normal distribution was used to approximate the true distributions of ADHD-RS-IV change. To impute CGI-I response, the proportion of the approximating normal curve below the ADHD-RS-IV change cutoff was then used as the imputed CGI-I response.

Each cut-off was used to extrapolate the proportion of patients achieving CGI-I of 1 or 2 from the mean and standard deviation (SD) of the ADHD-RS-IV change from baseline. The distribution of ADHD-RS-IV change from baseline was assumed to be normally distributed with the corresponding mean and SD. The extrapolated proportion equaled the probability of having an ADHD-RS-IV decrease from baseline greater than the cutoff under this distribution.

For each cut-off identified, the sensitivity and specificity of the prediction in the pooled data was calculated. Sensitivity was defined as the proportion of patients who in fact achieved CGI-I response who were correctly classified by the imputation analysis prediction. Specificity was defined as the proportion of patients who did not in fact achieve CGI-I response who were correctly classified by the imputation analysis prediction.

## Validation of Extrapolation

The extrapolation methods were validated using available trials (IPD or publication) reporting both ADHD-RS-IV change and CGI-I response by comparing the reported CGI-I response with the imputed CGI-I response.

In the first validation exercise, the cutoffs described above were calculated using a random training sample equal to 2/3 of the SPD489-303 trial data. Validation was then performed on the 1/3 of the SPD489-303 trial data reserved to be the validation set. In the second validation exercise, extrapolated CGI-I response rates were compared to the actual rates in trials reporting both CGI-I and ADHD-RS-IV.

## Incorporation into Bayesian and frequentist networks

After obtaining the imputed CGI-I response for ATX, the Bayesian and frequentist MTCs of CGI-I response were updated to incorporate ATX. Specifically, the percent CGI-I response was imputed for the ATX and placebo arms of Weisler (2012) and added to the network. Note that the MPH arm of Weisler (2012) was not used.

## Extrapolation from AISRS using the Goodman and direct methods

Using the same cutoffs evaluated in using the SPD489-303 trial, CGI-I was extrapolated using the Goodman method with quadratic regression and direct method in three ATX studies reporting AISRS: Adler (2009a), Sutherland (2012), and Young (2011). The cutoffs were validated in Biederman 2006, the one included study reporting both AISRS and CGI-I. These extrapolations were then used in addition to the prior extrapolation based on ADHD-RS-IV (Weisler (2012)) in the MTC.

# Results

## Selection of studies

The PRISMA flowchart in [**Figure 1**](#fig01) illustrates the trial selection process for the core efficacy and safety analyses.

A total of 5,358 records were retrieved from searching bibliographic databases and clinical trial registries, and an additional 725 records were retrieved from APA conference proceedings, and the NICE registry. After removal of duplicates, a total of 4,672 records were identified in the literature search. Of these, 4,099 records were excluded based on the title and/or abstract, most commonly due to the study pertaining to an irrelevant population, such as children with ADHD, or populations with a disease other than ADHD. Other studies excluded at this stage included records not pertaining to relevant pharmacological interventions, preclinical, phase I, prognostic, or retrospective studies, nonsystematic reviews, and other irrelevant publication types, such as commentaries and letters. Full-text articles of the remaining 573 records were assessed. In addition, 1 article was identified through hand-search and included during the full-text screening stage. After initial full-text screening, 157 articles were selected for further consideration. A total of 21 articles reporting clinical evidence for LDX, ATX, or MPH-OROS/ER were selected for the efficacy and/or safety (core and sensitivity) analyses. One publication reported on two identical trials, so these two trials were pooled and treated as one study for the analysis. In the core efficacy analysis, 6 records were included in the network for ADHD-RS-IV, and 5 for CGI-I. The safety analysis was conducted using 18 records for all-cause discontinuation, and 18 records for discontinuation due to adverse events. One additional study reporting ADHD-RS-IV change was included for a sensitivity analysis only. In the analysis of change from baseline in ADHD-RS-IV or AISRS total score, 6 records with AISRS were included.

**Figure 1. PRISMA Diagram**

**Notes:**

1. Includes non-human studies, not an ADHD population, and studies focusing on children and adolescents only (i.e., no adults included).
2. Includes only treatments other than methylphenidate, atomoxetine, amphetamine/dexamphetamine, or lisdexamfetamine
3. Includes preclinical studies, phase I studies, prognostic studies, retrospective studies, case reports, audio files, commentaries and letters (publication type), consensus reports, nonsystematic reviews, or titles without corresponding abstracts (Title/Abstract Screening only).
4. For title/abstract screening, studies reporting only pharmacokinetics, biochemical, neuroimaging, or genetic outcomes were excluded. For full text screening, studies that did not report all-cause discontinuation, adverse events discontinuation, or at least one of the following efficacy measures commonly used in adult ADHD: CAARS, ADHD-RS-IV, BAARS, BADDS, CGI scales, or GAF.
5. Includes studies for which the full text is not available for open access download or for purchase.
6. Includes non-randomized studies, open label extensions of randomized controlled trials, systematic reviews, post-hoc analyses, pooled analyses, or meta-analyses.
7. Other population restrictions include women only, mothers only, with spouse and child only, and prison inmates.
8. Includes trials with neither safety nor efficacy outcomes of interest.
9. Includes randomized withdrawal trials (N=2) and one single-blind trial (N=1).
10. Includes trials that were withdrawn, are ongoing, or currently recruiting participants on clinicaltrials.gov.
11. One trial, Spencer (1998), was included in sensitivity analyses only because it was a cross-over trial in which it was unclear whether reported results were from before the cross-over.
12. One study, Michelson (2003), consisted of two identical trials conducted in parallel. Data for these two trials were pooled for use in the MTC.

### Trial characteristics

Among the 21 eligible trials included in core and/or sensitivity analyses, the publication dates were all after 1998 (year 1998-2013). 19 of these trials were randomized, double-blind controlled trials, 1 trial was partly blinded, and 1 trial had a cross-over design. The per-arm randomized sample size ranged from 22 to 268. The characteristics of the RCTs selected for the efficacy and/or safety analyses of LDX, MPH-OROS/ER, and ATX for the treatment of adult ADHD can be provided on request.

All studies required patients to be at least 18 years old. Common exclusion criteria for patient selection within the studies included: current, uncontrolled, comorbid psychiatric diagnosis; preexisting cardiovascular disorders; individuals with low intelligence; and alcohol or drug abuse.

At baseline, the mean age of the study populations ranged between 21 and 43 years. Mean baseline ADHD-RS-IV scores ranged approximately from 29 to 41. The percent of females in the study ranged from 31.5% to 68%.

The quality of the methodology in each of the 21 trials was assessed, and all were determined to be of suitable quality. In one study, the authors – Young et al (2011), – reported a programming error that occurred in the stratification block that resulted in more patients having a 6 to 12 year old child without ADHD being randomized to the no-label titration ATX titration group. However, this imbalance in the arms did not seem to impact the results, as was stated in the paper. The full quality assessment can be provided on request.

### Average Dose

The average mean final dose in included arms weighted by sample size was calculated for each arm of studies used in the following analyses:

- ADHD-RS-IV change core analysis
- ADHD-RS-IV/AISRS change core analysis
- CGI-I response core analysis
- Adverse events discontinuation core analysis
- ADHD-RS-IV change or CGI-I response core analyses
- ADHD-RS-IV/AISRS change or CGI-I response core analyses

The average mean final dose calculated in LDX arms ranged from 51.38 mg/day to 51.50 mg/day. The average dose for ATX ranged from 80.00 mg/day to 87.67 mg/day. The average dose for MPH-ER ranged from 36.88 mg/day to 58.95 mg/day. The average dose of MPH-IR was available only for the adverse events discontinuation analysis and was 82.00 mg/day. These average doses were consistent with the recommended doses of these drugs for adults. Average doses for each analysis can be provided on request.

## Summary of Bayesian MTC analysis

Network diagrams depicting studies included in core or sensitivity analyses for each outcome by treatment arm are available on request.

### Bayesian core analysis for ADHD-RS-IV change

As shown in **Table 3A**, LDX had a numerically larger change from baseline than both ATX and MPH-ER in the core analysis. Although the 95% credible intervals of the estimates of treatment for the drugs overlapped, LDX had a high probability (> 90%) of being the most efficacious treatment compared to ATX and MPH-ER. The histograms of the posterior distributions show that while there was overlap in the distributions of the posterior samples for the three treatments, the distribution for LDX was concentrated at a greater negative ADHD-RS-IV change than the other two treatments.

| **Table 3A. Effect of treatment on change from baseline in ADHD-RS-IV score (drug - placebo)^[[1]](#footnote-1)^** | | | | | | | |
| --- | --- | --- | --- | --- | --- | --- | --- |
| **Core Bayesian analysis: random effects model, combined doses** | | | | | | | |
|  |  |  |  |  |  |  |  |
|  |  |  | **Mean** | **95% Credible Interval** | **Probability of the treatment being most effective among all** | **Probability of LDX being more effective compared to each treatment** |  |
|  | **LDX** |  | -10.48 | (-13.28, -7.75) | 90.67% | -- |  |
|  | **ATX** |  | -6.42 | (-11.47, -1.49) | 7.39% | 92.26% |  |
|  | **MPH-ER** |  | -6.73 | (-9.34, -4.04) | 1.94% | 97.39% |  |

### Bayesian core analysis for ADHD-RS-IV/AISRS change

In the core analysis of changes in ADHD-RS-IV or AISRS total score presented in **Table 3B**, LDX had the largest decrease in the score (-10.45) compared to both ATX (-5.41) and MPH-ER (-5.83). There was no overlap in the 95% credible intervals, indicating that the decrease in the score for LDX was statistically different from score changes for both ATX and MPH-ER. LDX also had a very high probability of being the most efficacious treatment compared to ATX (99.92%) and MPH-ER (99.90%). The histograms of the posterior distributions show that the distribution for LDX was concentrated at a greater negative ADHD-RS-IV/AISRS change than the other two treatments, and had very little overlap with the posterior distributions for ATX and MPH-ER.

| **Table 3B. Effect of treatment on change from baseline in ADHD-RS-IV/AISRS score (drug - placebo)^[[2]](#footnote-2)^** | | | | | | | |
| --- | --- | --- | --- | --- | --- | --- | --- |
| **Core Bayesian analysis: random effects model, combined doses** | | | | | | | |
|  |  |  |  |  |  |  |  |
|  |  |  | **Mean** | **95% Credible Interval** | **Probability of the treatment being most effective among all** | **Probability of LDX being more effective compared to each treatment** |  |
|  | **LDX** |  | -10.45 | (-12.94, -8.10) | 99.83% | -- |  |
|  | **ATX** |  | -5.41 | (-7.24, -3.59) | 0.08% | 99.92% |  |
|  | **MPH-ER** |  | -5.83 | (-7.36, -4.29) | 0.09% | 99.90% |  |

### Bayesian core analysis for CGI-I response

As shown in **Table 4**, LDX had higher point estimates of relative risk and odds ratio of response than MPH-ER in the core analysis: estimated relative risk of 2.18 vs. 1.85 and estimated odds ratio of 4.50 vs. 2.94, respectively. LDX also had a high probability (87.69%) of being more efficacious than MPH-ER. The histograms of the posterior distributions show that while there was overlap in the posterior samples of LDX and MPH-ER, LDX had a high probability of having a higher relative risk for CGI-I response than MPH-ER.

In a further analysis, CGI-I response data for ATX was extrapolated and added to the network. See **Section 5.5.3** for Bayesian MTC results incorporating ATX.

| **Table 4. Odds ratios and relative risks for treatment response, as defined by a rating of 1 or 2 in CGI-I score (drug vs. placebo)^[[3]](#footnote-3)^** | | | | | | |
| --- | --- | --- | --- | --- | --- | --- |
| **Core Bayesian analysis: random effects model, combined doses** | | | | | | |
|  |  |  |  |  |  |  |
|  |  |  | **Odds Ratio** | **95% Credible Interval** | **Probability of the treatment being most effective among all** | **Probability of LDX being more effective compared to each treatment** |
|  | **LDX** |  | 4.50 | (2.56, 7.85) | 87.69% | -- |
|  | **ATX** |  | -- | -- | -- | -- |
|  | **MPH-ER** |  | 2.94 | (1.82, 4.74) | 12.31% | 87.69% |
|  |  |  |  |  |  |  |
|  |  |  | **Relative risk of drug vs. placebo** | **95% Credible interval for relative risk** | **Response rate** | **Placebo Risk**  **(95% CI)^[[4]](#footnote-4)^** |
|  | **LDX** |  | 2.18 | (1.73, 2.64) | 0.66 | 0.3012 |
|  | **ATX** |  | -- | -- | -- | (0.253, 0.350) |
|  | **MPH-ER** |  | 1.85 | (1.46, 2.27) | 0.56 |  |

### Bayesian core analysis for all-cause discontinuation

As shown in **Table 5**, LDX had the lowest odds ratio for all-cause discontinuation (0.80) in the core analysis, followed by MPH-IR (1.10), ATX (1.15), and MPH-ER (1.17). On the relative risk scale, the average relative risk of ATX, MPH-ER, and MPH-IR are very similar (1.11, 1.12, and 1.12, respectively), while that for LDX is lower (0.87). Overlap in the credible intervals is observed, but LDX has the highest probability of having the lowest all-cause discontinuation of all treatments and has high probabilities of having a lower all-cause discontinuation rate than ATX and MPH-ER.

| **Table 5. Odds ratios and relative risks for all-cause discontinuation (drug vs. placebo)** | | | | | | | | |
| --- | --- | --- | --- | --- | --- | --- | --- | --- |
| **Core Bayesian analysis: random effects model, combined doses** | | | | | | | | |
|  |  |  |  |  |  |  |  |  |
|  |  |  | **Odds Ratio** | **95% Credible Interval** | **Probability of the treatment being most tolerable among all** | **Probability of LDX being more tolerable compared to each treatment** |  |  |
|  | **LDX** |  | 0.80 | (0.38, 1.70) | 55.45% | -- |  |  |
|  | **ATX** |  | 1.15 | (0.73, 1.77) | 8.79% | 80.19% |  |  |
|  | **MPH-ER** |  | 1.17 | (0.81, 1.69) | 6.46% | 82.00% |  |  |
|  | **MPH-IR** |  | 1.10 | (0.32, 3.98) | 29.30% | 66.60% |  |  |
|  |  |  |  |  |  |  |  |  |
|  |  |  | **Relative risk of drug vs. placebo** | **95% Credible interval for relative risk** | **Response rate** | **Placebo Risk**  **(95% CI)^[[5]](#footnote-5)^** |  |  |
|  | **LDX** |  | 0.87 | (0.46, 1.42) | 0.24 | 0.2759 |  |  |
|  | **ATX** |  | 1.11 | (0.79, 1.46) | 0.31 | (0.256, 0.296) |  |  |
|  | **MPH-ER** |  | 1.12 | (0.86, 1.42) | 0.31 |  |  |  |
|  | **MPH-IR** |  | 1.12 | (0.39, 2.19) | 0.31 |  |  |  |

### Bayesian core analysis for discontinuation due to adverse events

As shown in **Table 6**, ATX had the lowest relative risk for discontinuation due to adverse events (2.67) in the core analysis, followed by MPH-ER (2.76), LDX (3.21), and MPH-IR (4.38). LDX had the highest probability of being the most tolerable (31.01%). LDX had a higher probability of being the most tolerable treatment than ATX despite a higher relative risk because the distribution of LDX was more spread compared to the distribution of ATX. The histograms of the posterior distributions from this MTC are available on request. The probabilities of each treatment being the most tolerable were more similar than in the efficacy analyses.

| **Table 6. Odds ratios and relative risks for discontinuation due to adverse events (drug vs. placebo)** | | | | | | | | |
| --- | --- | --- | --- | --- | --- | --- | --- | --- |
| **Core Bayesian analysis: random effects model, combined doses** | | | | | | | | |
|  |  |  |  |  |  |  |  |  |
|  |  |  | **Odds Ratio** | **95% Credible Interval** | **Probability of the treatment being most tolerable among all** | **Probability of LDX being more tolerable compared to each treatment** |  |  |
|  | **LDX** |  | 3.11 | (0.93, 11.69) | 31.01% | -- |  |  |
|  | **ATX** |  | 2.82 | (1.73, 4.83) | 24.70% | 44.02% |  |  |
|  | **MPH-ER** |  | 2.94 | (1.90, 4.75) | 18.87% | 46.87% |  |  |
|  | **MPH-IR** |  | 4.17 | (0.82, 28.42) | 25.42% | 59.31% |  |  |
|  |  |  |  |  |  |  |  |  |
|  |  |  | **Relative risk of drug vs. placebo** | **95% Credible interval for relative risk** | **Response rate** | **Placebo Risk**  **(95% CI)^[[6]](#footnote-6)^** |  |  |
|  | **LDX** |  | 3.21 | (0.93, 7.90) | 0.14 | 0.0443 |  |  |
|  | **ATX** |  | 2.67 | (1.68, 4.13) | 0.12 | (0.035, 0.053) |  |  |
|  | **MPH-ER** |  | 2.76 | (1.83, 4.07) | 0.12 |  |  |  |
|  | **MPH-IR** |  | 4.38 | (0.83, 12.80) | 0.19 |  |  |  |

### Bayesian sensitivity analyses

**ADHD-RS-IV change**

In the combined dose sensitivity analyses, LDX continued to have the highest probability of being the most efficacious treatment. When using a fixed effects model, or adding and removing studies to the random effects model, the probability of LDX being the most effective was still highest, followed by ATX and MPH-ER. When separating the arms out by dose, LDX 70 mg/day had the highest probability of being the most efficacious treatment, followed by MPH-ER 40 mg/day, LDX 50 mg/day, and LDX 30 mg/day. In all sensitivity analyses, there continued to be overlap in the credible intervals for all three treatments.

**ADHD-RS-IV or AISRS change**

In the fixed effects model with combined doses, LDX continued to have higher efficacy than both ATX and MPH-ER. The 95% credible intervals did not overlap indicating that the decrease in the ADHD-RS-IV or AISRS scores for LDX was statistically different from the score decrease for both ATX and MPH-ER.

**CGI-I response**

In the fixed effects model with combined doses, LDX continued to have higher efficacy than MPH-ER. However, there was overlap in the credible intervals for LDX and MPH-ER.

**All-cause discontinuation**

In the sensitivity analyses, LDX continued to have the lowest relative risk of all-cause discontinuation. In the fixed effects model with combined doses, the posterior probability of being the most tolerable treatment was highest for LDX, followed by MPH-IR, MPH-ER, and ATX. When removing the young adult studies from the core analysis (Biederman 2012 for LDX and Durell 2013 for ATX), this probability was highest for LDX, followed by MPH-IR, ATX, and MPH-ER. In all sensitivity analyses, there continued to be overlap in the credible intervals for all four treatments.

**Discontinuation due to adverse events**

In the fixed effects model with combined doses, the probability of being the most tolerable treatment was highest for LDX, followed by ATX, MPH-ER, and MPH-IR. When removing the young adult studies from the core analysis (Biederman 2012 for LDX and Durell 2013 for ATX), the posterior probability of being the most tolerable treatment was highest for ATX, followed by LDX, MPH-IR, and MPH-ER. In all sensitivity analyses, there continued to be overlap in the credible intervals for all four treatments.

## Frequentist analysis results

**ADHD-RS-IV change**

In the frequentist analysis, the results of the random effects and fixed effects models with combined doses were identical to one another in both mean and confidence interval and were similar to the Bayesian fixed effects analysis. For example, in the frequentist analysis, LDX had a mean change of -10.41 and 95% confidence interval of (-12.60, -8.22), while in the Bayesian fixed effects analysis, LDX had a mean change of -10.41 and 95% credible interval of (-12.60, -8.21). For both random effects and fixed effects, LDX had the greatest ADHD-RS-IV change (-10.41), followed by MPH-ER (-6.73) and ATX (-6.43). When adding in studies (Spencer 1998 for ATX in sensitivity analysis 2) or removing studies (NCT00937040 in sensitivity analysis 4 and Biederman 2012 for LDX in sensitivity analysis 5), LDX continued to have the greatest ADHD-RS-IV change, followed by either ATX or MPH-ER. When separating out the doses, LDX 70 mg/day had the greatest ADHD-RS-IV change (-11.10), followed by LDX 50 mg/day (-9.66), MPH-ER 40 mg/day (-9.00), and LDX 30 mg/day (-8.46). No statistically significant separation in the confidence intervals was found.

**ADHD-RS-IV or AISRS change**

In the frequentist analysis, the results of the random effects and fixed effects models with combined doses were identical to one another in both mean and confidence interval and were similar to the results of the Bayesian fixed effects analysis. For both random effects and fixed effects, LDX had the greatest ADHD-RS-IV change (-10.41), followed by MPH-ER (-5.82) and ATX (-5.47). These results were statistically significant.

**CGI-I response**

The results of the frequentist random effects and fixed effects combined dose analyses were identical to each other (estimated relative risk of 2.23 for LDX and 1.80 for MPH-ER) and similar to the results of Bayesian analysis (estimated relative risk of 2.18 for LDX and 1.85 for MPH-ER). For both random effects and fixed effects, LDX had greater efficacy than MPH-ER. However, no statistically significant separation in the confidence intervals for LDX and MPH-ER was found.

In a further analysis, CGI-I response data for ATX was extrapolated and added to the network. See **Section 5.5.4** for frequentist MTC results incorporating ATX.

**All-cause discontinuation**

The results of the frequentist random effects and fixed effects combined dose analyses were similar. They were also similar to the Bayesian analysis. For the random effects core analysis, LDX had the lowest relative risk (0.83) for all-cause discontinuation, followed by MPH-IR (1.05), ATX (1.09), and MPH-ER (1.11). This ranking of treatments was the same for sensitivity analysis excluding young adult studies. In the fixed effects model, LDX has the lowest relative risk (0.80), followed by MPH-IR (1.05) and MPH-ER (1.05), and then by ATX (1.18). No statistically significant separation in the confidence intervals was found.

**Discontinuation due to adverse events**

The results of the frequentist random effects and fixed effects combined dose analyses were identical. The relative risks for the ATX and MPH-ER arms were similar to the relative risks in the fixed effects Bayesian analysis, while the relative risks for LDX and MPH-IR were lower than in the fixed effects Bayesian analysis. For both random effects and fixed effects, ATX had the lowest relative risk for adverse events discontinuation (2.44), followed by MPH-ER (2.59), LDX (2.73), and MPH-IR (3.23). In the sensitivity analysis excluding young adult studies, ATX had the lowest relative risk (2.29), followed by MPH-ER (2.58), MHP-IR (3.23), and LDX (3.27). No statistically significant separation in the credible intervals was found.

## Heterogeneity and inconsistency assessments

In accordance with NICE guidelines on the assessment of heterogeneity, I^2^ and Cochran’s Q statistics were calculated for trials comparing LDX and placebo in the core analyses for the efficacy outcomes ADHD-RS-IV and CGI-I response (**Table 7**). The Q statistic, which tests for the presence of heterogeneity, is not statistically significant as indicated by the p-value for both ADHD-RS-IV and CGI-I. I^2^, which measures the degree of heterogeneity, is zero for both ADHD-RS-IV and CGI-I in the core analyses. This suggests that the source of variability in effect size estimates is the sampling error within studies, rather than between-study differences (Huedo-Medina et al, 2006).

The DIC was also compared between fixed effects and random effects models for the ADHD-RS-IV, CGI-I, and ADHD-RS-IV/AISRS networks. Again, the results were qualitatively similar for both all three efficacy outcomes (**Table 8**). Specifically, the fixed effects model had a lower DIC, and thus a better fit, than the random effects model in each case, indicating that heterogeneity is low in the network overall. In addition, the two types of models produced very similar estimates. The random effects models represent a more conservative choice.

The evidence network for ADHD-RS-IV included only one loop that had both direct and indirect evidence comparing ATX and MPH (**Figure 2**). Following NICE guidelines, the Bucher method was applied to this section of network to evaluate inconsistency (Dias et al, 2011). Specifically, a two-stage assessment was conducted: the first stage involved measuring and synthesizing the indirect treatment effect from the ATX vs. placebo and MPH vs. placebo comparisons, and computing its standard error accounting for correlation due to a common placebo arm.

**Figure 2. Efficacy outcome: ADHD-RS-IV score**

**Abbreviations**: LDX: lisdexamfetamine; ATX: atomoxetine; MPH-OROS/ER: methylphenidate extended release; MPH-IR: methylphenidate immediate release.

**Notes:**

1. All studies not marked with an asterisk are included in the core analysis. Studies marked with an asterisk are included only in sensitivity analyses.
2. Biederman (2012) and Durell (2013) are included in the core analysis but are the only two studies containing only young adults (all participants are 30 years old or younger). The tables include sensitivity analyses in which Biederman (2012) is excluded for ADHD-RS-IV change, and Biederman (2012) and Durell (2013) are excluded for all-cause discontinuation and discontinuation due to adverse events.
3. Spencer (1998), a cross-over study, is not included in the core analysis because it is unclear whether the results reported are pre–cross-over. The tables include a sensitivity analysis in which Spencer (1998) is included for ADHD-RS-IV change.
4. Among MPH-OROS/ER studies, only Spencer (2007) is MPH-ER. The remaining studies use the MPH-OROS formulation.
5. NCT00937040 is included in the core analysis, but there is a sensitivity for the ADHD-RS-IV outcome in which it is excluded because it reports ADHD-RS-IV rated by significant others.

The second stage included analysis of whether the direct ATX vs. MPH treatment effect estimate was in conflict with the indirect estimate, using a standard error estimate accounting for correlation due to common ATX and MPH arms. Statistical comparison revealed that there was no significant inconsistency in the section of ADHD-RS-IV network comparing ATX and MPH **(Table 9)**.

| **Table 7. Heterogeneity Assessment: LDX vs. Placebo** | | | | | |
| --- | --- | --- | --- | --- | --- |
| **I^2^ and Q test** | | | | | |
| **Outcome** | **I^2^** | **I^2^ Confidence Interval** | **Q-statistic** | **Q Degrees  of Freedom** | **Q  p-value** |
| **ADHD-RS-IV** | 0.00 | (0.00, 0.86) | 1.52 | 2 | 0.47 |
| **CGI-I** | 0.00 | --^[1]^ | 0.24 | 1 | 0.63 |

| **Table 8. Heterogeneity Assessment** | | |
| --- | --- | --- |
| **Deviance Information Criterion (DIC)** | | |
| **Outcome** | **Random Effects** | **Fixed Effects** |
| **ADHD-RS-IV** | 70.19 | 69.27 |
| **ADHD-RS-IV or AISRS** | 111.05 | 108.51 |
| **CGI-I** | 86.55 | 84.91 |

| **Table 9. Inconsistency Assessment: Bucher Method**  **Indirect vs. Direct Comparison of MPH vs. ATX in the Core ADHD-RS-IV Analysis** | | | |
| --- | --- | --- | --- |
|  |  |  |  |
| **Treatment Effect Estimates** | | **Mean of  ADHD-RS-IV Improvement** | **Standard Error of ADHD-RS-IV Improvement** |
| Direct (MPH vs. placebo)^[2]^ | | -6.00 | 0.94 |
| Direct (ATX vs. placebo) | | -6.50 | 2.18 |
| **Indirect** (MPH vs. ATX)^[3]^ | | 0.50 | 2.20 |
| **Direct** (MPH vs. ATX) | | -0.40 | 2.35 |
| **Indirect (MPH vs. ATX) vs. Direct  (MPH vs. ATX)^[4]^** | |  |  |
|  |  | 0.90 | 1.84 |
| p-value |  | 0.62 | |

**Notes:**

1. The CGI-I evidence network contained only 2 studies with LDX and placebo arms in the core analysis.
2. The ADHD-RS-IV mean change and standard error were first computed by pooling all MPH arms, weighted by sample size, in Spencer (2007). The mean and standard error of the direct MPH vs. placebo treatment effect were then computed using the pooled values.
3. Calculations of the standard error of the indirect MPH vs. ATX treatment effect incorporate covariance due to a common placebo arm.
4. Calculations of the standard error of the difference of indirect and direct MPH vs. ATX treatment effects incorporate covariance due to common ATX and MPH arms.
5. In one study with multiple LDX arms (Adler, 2008), the ADHD-RS-IV mean change and standard deviation were first computed by pooling all LDX arms, weighted by sample size. Similarly, the CGI-I response rate and sample size were computed by pooling all LDX arms. The I^2^ and Q statistics were then computed using the pooled values. The Q statistic tests for the presence of heterogeneity. I^2^ measures the degree of heterogeneity. For example, I^2^ = 0 implies that all variability in effect size estimates is due to sampling error within studies. I^2^= 50 means that half of the total variability among effect sizes is caused not by sampling error, but by true heterogeneity between studies.
6. The Deviance Information Criterion (DIC) is a measure of the fit of the fixed effects and random effects models. Lower DIC indicates a better fit. A better fit (lower DIC) for the fixed effects model would indicate that between-study heterogeneity in the effect size is low.
7. The Bucher method assesses inconsistency in the ATX-MPH-placebo loop in the ADHD-RS-IV evidence network, whereby the direct ATX vs. MPH treatment effect estimate is compared against the difference of the ATX vs. placebo and MPH vs. placebo treatment effect estimates (i.e., the indirect estimate).

## Imputation Analysis Results

### Calculation of cut-off values

As shown by the histogram, Q-Q plot, and estimated CDF plots in **Step 1** of **Appendix B**, the ADHD-RS-IV score change data in the SPD489-303 trial was approximately normally distributed. In particular, the data showed excellent agreement with the normal distribution except in the tails, which was less of a concern since the cutoffs were not located in the tails.

Two models, linear and quadratic, were evaluated in the Goodman method relating CGI-I and ADHD-RS-IV. The quadratic model was chosen for two reasons: (1) the relationship between CGI-I and ADHD-RS-IV in the SPD489-303 IPD did not appear linear and the quadratic model provided better fit (visually as well as using the R^2^ statistic), and (2) during validation in other trials, the quadratic model consistently overestimated the percent response in the ATX arm, which would provide a conservative estimate for LDX performance once this information was incorporated into the network. The Goodman method with a linear model consistently underestimated the percent response in the ATX arm, and was therefore not used.

The direct method alternated between overestimating and underestimating and was therefore used as a more realistic estimate compared to the conservative estimate using the Goodman method with quadratic regression.

**Step 2** summarizes the results of the Goodman method with quadratic regression, which produced an ADHD-RS-IV change distribution cutoff of -13.636 points, while the direct estimation method produced a cutoff of -14.405. The sensitivity and specificity were used to judge how well the cutoff was performing. Sensitivity and specificity were relatively high for both methods, with all values near 90 percent. For the Goodman method with quadratic regression, the sensitivity was slightly higher than the specificity. For the direct estimation method, the specificity was slightly higher than the sensitivity.

### In-trial and out of trial validation results

**Step 3** shows the validation performance of these cutoffs in predicting the percent response in CGI-I. The bias in the validation sample of the SPD489-303 trial IPD was 3.2 percentage points with the (quadratic) Goodman method and 0.6 percentage points with the direct method. The extrapolated rates of response were similar to actual rates when extrapolating in trials reporting both CGI-I and ADHD-RS-IV or both CGI-I and AISRS. The bias under the Goodman method ranged from 1.0 to 12.0 percentage points. The bias under direct estimation ranged in absolute value from 0.1 percentage points to 8.8 percentage points. The results using the Goodman method with quadratic regression were conservative in the sense that they consistently overestimated the rate of response in ATX patients, while the direct estimation method fluctuated between under and over-estimation.

### Bayesian MTC results incorporating data extrapolated from ADHD-RS-IV

Adding CGI-I response of ATX imputed from ADHD-RS-IV in the imputation analyses to the Bayesian MTC, LDX continued to be the treatment with the highest probability of being the most effective. The two methods of cutoff estimation ultimately yielded similar MTC results. Using the Goodman method for the imputation analysis, LDX had the highest probability of being the most effective treatment (80.62%), followed by MPH-ER and ATX. Using the direct method for the imputation analysis, LDX had the highest probability of being the most effective treatment (81.83%), followed by MPH-ER and ATX. Complete Bayesian MTC results are presented in **Table 10** and **Table 11**. The posterior distribution of LDX was concentrated at a higher relative risk for CGI-I response than MPH-ER and ATX. This was observed when using both the Goodman and direct estimation methods.

| **Table 10. Odds ratios and relative risks for treatment response, as defined by a rating of 1 or 2 in CGI-I score (drug vs. placebo)** | | | | | | |
| --- | --- | --- | --- | --- | --- | --- |
| **Quadratic regression (Goodman) extrapolation method for ATX arm (random effects model, combined doses)** | | | | | | |
|  |  |  |  |  |  |  |
|  |  |  | **Odds Ratio** | **95% Credible Interval** | **Probability of the treatment being most effective among all** | **Probability of LDX being more effective compared to each treatment** |
|  | **LDX** |  | 4.37 | (2.55, 7.67) | 80.62% | -- |
|  | **ATX** |  | 2.32 | (0.99, 5.43) | 8.19% | 90.35% |
|  | **MPH-ER** |  | 2.94 | (1.79, 4.74) | 11.19% | 86.88% |
|  |  |  |  |  |  |  |
|  |  |  | **Relative risk of drug vs. placebo** | **95% Credible interval for relative risk** | **Response rate** | **Placebo Risk**  **(95% CI)^[[7]](#footnote-7)^** |
|  | **LDX** |  | 2.14 | (1.71, 2.57) | 0.66 | 0.3084 |
|  | **ATX** |  | 1.65 | (1.00, 2.32) | 0.51 | (0.264, 0.353) |
|  | **MPH-ER** |  | 1.84 | (1.44, 2.23) | 0.57 |  |

| **Table 11. Odds ratios and relative risks for treatment response, as defined by a rating of 1 or 2 in CGI-I score (drug vs. placebo)** | | | | | | |
| --- | --- | --- | --- | --- | --- | --- |
| **Direct extrapolation method for ATX arm (random effects model, combined doses)** | | | | | | |
|  |  |  |  |  |  |  |
|  |  |  | **Odds Ratio** | **95% Credible Interval** | **Probability of the treatment being most effective among all** | **Probability of LDX being more effective compared to each treatment** |
|  | **LDX** |  | 4.45 | (2.59, 7.74) | 81.83% | -- |
|  | **ATX** |  | 2.37 | (1.01, 5.57) | 8.59% | 89.88% |
|  | **MPH-ER** |  | 2.90 | (1.83, 4.69) | 9.58% | 88.54% |
|  |  |  |  |  |  |  |
|  |  |  | **Relative risk of drug vs. placebo** | **95% Credible interval for relative risk** | **Response rate** | **Placebo Risk**  **(95% CI)^[[8]](#footnote-8)1^** |
|  | **LDX** |  | 2.17 | (1.74, 2.61) | 0.66 | 0.3036 |
|  | **ATX** |  | 1.67 | (1.01, 2.35) | 0.51 | (0.259, 0.348) |
|  | **MPH-ER** |  | 1.84 | (1.45, 2.24) | 0.56 |  |

### Frequentist MTC results incorporating data extrapolated from ADHD-RS-IV

Adding CGI-I response of ATX imputed from ADHD-RS-IV in the imputation analyses to the frequentist MTC, LDX continued to be the treatment with the highest probability of being the most effective. The two methods of cutoff estimation yielded ultimately similar MTC results. Using the Goodman method for the imputation analysis, LDX had the highest relative risk (2.23), followed by MPH-ER (1.80) and ATX (1.60). Using the direct method for the imputation analysis, LDX again had the highest relative risk (2.23), followed by MPH-ER (1.80) and ATX (1.65). Complete frequentist MTC results are presented in **Table 12** and **Table 13**.

| **Table 12. Relative risks for treatment response, as defined by a rating of 1 or 2 in CGI-I score (drug vs. placebo)** | | | | |
| --- | --- | --- | --- | --- |
| **Quadratic regression (Goodman) extrapolation method for ATX arm (random effects model, combined doses)** | | | | |
|  |  |  |  |  |
|  |  |  | **Relative Risk^[[9]](#footnote-9)^** | **95% Confidence Interval** |
|  | **LDX** |  | 2.23 | (1.69, 2.95) |
|  | **ATX** |  | 1.60 | (1.09, 2.34) |
|  | **MPH-ER** |  | 1.80 | (1.43, 2.27) |

| **Table 13. Relative risks for treatment response, as defined by a rating of 1 or 2 in CGI-I score (drug vs. placebo)** | | | | |
| --- | --- | --- | --- | --- |
| **Direct extrapolation method for ATX arm (random effects model, combined doses)** | | | | |
|  |  |  |  |  |
|  |  |  | **Relative Risk^[[10]](#footnote-10)^** | **95% Confidence Interval** |
|  | **LDX** |  | 2.23 | (1.69, 2.95) |
|  | **ATX** |  | 1.65 | (1.10, 2.47) |
|  | **MPH-ER** |  | 1.80 | (1.43, 2.27) |

### Bayesian MTC results incorporating data extrapolated from ADHD-RS-IV and AISRS

Adding CGI-I response of ATX imputed from ADHD-RS-IV and AISRS in the imputation analyses to the Bayesian MTC, LDX continued to be the treatment with the highest probability of being the most effective. The two methods of cutoff estimation ultimately yielded similar MTC results. Using the Goodman method for the imputation analysis and a random effects model, LDX had the highest probability of being the most effective treatment (88.06%), followed by MPH-ER and ATX. Under the fixed effects models, the probability of LDX being the most efficacious treatment was 91.49%. Using the direct method for the imputation analysis and a random effects model, LDX had the highest probability of being the most effective treatment (89.30%), followed by MPH-ER and ATX. Under the fixed effects models, the probability of LDX being the most efficacious treatment was 91.34%. Complete Bayesian MTC results for random and fixed effects are presented in **Tables 14-17**. The posterior distribution of LDX was concentrated at a higher relative risk for CGI-I response than MPH-ER and ATX. This was observed when using both the Goodman and direct estimation methods.

| **Table 14. Odds ratios and relative risks for treatment response, as defined by a rating of 1 or 2 in CGI-I score (drug vs. placebo)** | | | | | | |
| --- | --- | --- | --- | --- | --- | --- |
| **Quadratic regression (Goodman) extrapolation method for ATX arm (random effects model, combined doses)** | | | | | | |
|  |  |  |  |  |  |  |
|  |  |  | **Odds Ratio** | **95% Credible Interval** | **Probability of the treatment being most effective among all** | **Probability of LDX being more effective compared to each treatment** |
|  | **LDX** |  | 4.37 | (2.67, 7.47) | 88.06% | -- |
|  | **ATX** |  | 1.92 | (1.42, 2.63) | 0.18% | 99.51% |
|  | **MPH-ER** |  | 2.93 | (1.85, 4.72) | 11.76% | 88.20% |
|  |  |  |  |  |  |  |
|  |  |  | **Relative risk of drug vs. placebo** | **95% Credible interval for relative risk** | **Response rate** | **Placebo Risk**  **(95% CI)^[[11]](#footnote-11)^** |
|  | **LDX** |  | 1.99 | (1.67, 2.30) | 0.70 | 0.3537 |
|  | **ATX** |  | 1.45 | (1.23, 1.67) | 0.51 | (0.323, 0.384) |
|  | **MPH-ER** |  | 1.74 | (1.42, 2.05) | 0.61 |  |

| **Table 15. Odds ratios and relative risks for treatment response, as defined by a rating of 1 or 2 in CGI-I score (drug vs. placebo)** | | | | | | |
| --- | --- | --- | --- | --- | --- | --- |
| **Quadratic regression (Goodman) extrapolation method for ATX arm (fixed effects model, combined doses)** | | | | | | |
|  |  |  |  |  |  |  |
|  |  |  | **Odds Ratio** | **95% Credible Interval** | **Probability of the treatment being most effective among all** | **Probability of LDX being more effective compared to each treatment** |
|  | **LDX** |  | 4.45 | (2.85, 7.02) | 91.49% | -- |
|  | **ATX** |  | 1.91 | (1.52, 2.39) | 0.01% | 99.96% |
|  | **MPH-ER** |  | 2.92 | (1.97, 4.34) | 8.51% | 91.49% |
|  |  |  |  |  |  |  |
|  |  |  | **Relative risk of drug vs. placebo** | **95% Credible interval for relative risk** | **Response rate** | **Placebo Risk**  **(95% CI)^[[12]](#footnote-12)^** |
|  | **LDX** |  | 2.00 | (1.71, 2.28) | 0.71 | 0.3537 |
|  | **ATX** |  | 1.44 | (1.28, 1.61) | 0.51 | (0.323, 0.384) |
|  | **MPH-ER** |  | 1.74 | (1.46, 2.01) | 0.61 |  |

| **Table 16. Odds ratios and relative risks for treatment response, as defined by a rating of 1 or 2 in CGI-I score (drug vs. placebo)** | | | | | | |
| --- | --- | --- | --- | --- | --- | --- |
| **Direct extrapolation method for ATX arm (random effects model, combined doses)** | | | | | | |
|  |  |  |  |  |  |  |
|  |  |  | **Odds Ratio** | **95% Credible Interval** | **Probability of the treatment being most effective among all** | **Probability of LDX being more effective compared to each treatment** |
|  | **LDX** |  | 4.40 | (2.63, 7.48) | 89.30% | -- |
|  | **ATX** |  | 1.92 | (1.41, 2.63) | 0.20% | 99.52% |
|  | **MPH-ER** |  | 2.91 | (1.85, 4.54) | 10.50% | 89.42% |
|  |  |  |  |  |  |  |
|  |  |  | **Relative risk of drug vs. placebo** | **95% Credible interval for relative risk** | **Response rate** | **Placebo Risk**  **(95% CI)^[[13]](#footnote-13)^** |
|  | **LDX** |  | 2.03 | (1.69, 2.36) | 0.69 | 0.3411 |
|  | **ATX** |  | 1.46 | (1.24, 1.69) | 0.50 | (0.311, 0.371) |
|  | **MPH-ER** |  | 1.76 | (1.43, 2.07) | 0.60 |  |

| **Table 17. Odds ratios and relative risks for treatment response, as defined by a rating of 1 or 2 in CGI-I score (drug vs. placebo)** | | | | | | |
| --- | --- | --- | --- | --- | --- | --- |
| **Direct extrapolation method for ATX arm (fixed effects model, combined doses)** | | | | | | |
|  |  |  |  |  |  |  |
|  |  |  | **Odds Ratio** | **95% Credible Interval** | **Probability of the treatment being most effective among all** | **Probability of LDX being more effective compared to each treatment** |
|  | **LDX** |  | 4.44 | (2.83, 7.10) | 91.34% | -- |
|  | **ATX** |  | 1.90 | (1.52, 2.38) | 0.01% | 99.97% |
|  | **MPH-ER** |  | 2.93 | (1.98, 4.37) | 8.65% | 91.35% |
|  |  |  |  |  |  |  |
|  |  |  | **Relative risk of drug vs. placebo** | **95% Credible interval for relative risk** | **Response rate** | **Placebo Risk**  **(95% CI)^[[14]](#footnote-14)^** |
|  | **LDX** |  | 2.04 | (1.74, 2.33) | 0.69 | 0.3411 |
|  | **ATX** |  | 1.45 | (1.29, 1.62) | 0.50 | (0.311, 0.371) |
|  | **MPH-ER** |  | 1.77 | (1.48, 2.05) | 0.60 |  |

### Frequentist MTC results incorporating data extrapolated from ADHD-RS-IV and AISRS

Adding CGI-I response of ATX imputed from the imputation analyses to the frequentist MTC, LDX continued to be the treatment with the highest probability of being the most effective. The two methods of cutoff estimation yielded ultimately similar MTC results. Using the Goodman method for the imputation analysis and a random effects model, LDX had the highest relative risk (2.23), followed by MPH-ER (1.80) and ATX (1.39). Using the direct method for the imputation analysis and a random effects model, LDX again had the highest relative risk (2.23), followed by MPH-ER (1.80) and ATX (1.41). Complete frequentist MTC results are presented in **Tables 18-21**.

| **Table 18. Relative risks for treatment response, as defined by a rating of 1 or 2 in CGI-I score (drug vs. placebo)** | | | | |
| --- | --- | --- | --- | --- |
| **Quadratic regression (Goodman) extrapolation method for ATX arm (random effects model, combined doses)** | | | | |
|  |  |  |  |  |
|  |  |  | **Relative Risk^[[15]](#footnote-15)^** | **95% Confidence Interval** |
|  | **LDX** |  | 2.23 | (1.69, 2.95) |
|  | **ATX** |  | 1.39 | (1.23, 1.58) |
|  | **MPH-ER** |  | 1.80 | (1.43, 2.27) |

| **Table 19. Relative risks for treatment response, as defined by a rating of 1 or 2 in CGI-I score (drug vs. placebo)** | | | | |
| --- | --- | --- | --- | --- |
| **Quadratic regression (Goodman) extrapolation method for ATX arm (fixed effects model, combined doses)** | | | | |
|  |  |  |  |  |
|  |  |  | **Relative Risk^[[16]](#footnote-16)^** | **95% Confidence Interval** |
|  | **LDX** |  | 2.23 | (1.69, 2.95) |
|  | **ATX** |  | 1.39 | (1.23, 1.58) |
|  | **MPH-ER** |  | 1.80 | (1.43, 2.27) |

| **Table 20. Relative risks for treatment response, as defined by a rating of 1 or 2 in CGI-I score (drug vs. placebo)** | | | | |
| --- | --- | --- | --- | --- |
| **Direct extrapolation method for ATX arm (random effects model, combined doses)** | | | | |
|  |  |  |  |  |
|  |  |  | **Relative Risk^[[17]](#footnote-17)^** | **95% Confidence Interval** |
|  | **LDX** |  | 2.23 | (1.69, 2.95) |
|  | **ATX** |  | 1.41 | (1.24, 1.60) |
|  | **MPH-ER** |  | 1.80 | (1.43, 2.27) |

| **Table 21. Relative risks for treatment response, as defined by a rating of 1 or 2 in CGI-I score (drug vs. placebo)** | | | | |
| --- | --- | --- | --- | --- |
| **Direct extrapolation method for ATX arm (fixed effects model, combined doses)** | | | | |
|  |  |  |  |  |
|  |  |  | **Relative Risk^[[18]](#footnote-18)^** | **95% Confidence Interval** |
|  | **LDX** |  | 2.23 | (1.69, 2.95) |
|  | **ATX** |  | 1.41 | (1.24, 1.60) |
|  | **MPH-ER** |  | 1.80 | (1.43, 2.27) |

# Discussion

## Summary of Results

Overall, the Bayesian analysis found that LDX had numerically better efficacy outcomes compared to other available pharmacologic treatments ATX and MPH. In one set of analyses – ADHD-RS-IV/AISRS change – the results were statistically significant. However, in all other efficacy analyses, there was overlap in the 95% posterior credible intervals of LDX and ATX or MPH. The Bayesian nature of our analysis allowed us to calculate an additional measure – the probability of being the most efficacious treatment – to provide additional information when no separation in credible intervals was observed. LDX was shown to have greater than 85% probability of being the most efficacious treatment for all combined dose analyses of both ADHD-RS-IV change and CGI-I response. To provide further confidence in these results, a frequentist analysis was also conducted, and the results were consistent with the Bayesian analysis. The clinical advantage of LDX in efficacy analyses was consistent across Bayesian and frequentist sensitivity analyses, in terms of both the treatment effect and the posterior probability of treatment superiority estimated from Bayesian analysis.

LDX had a lower relative risk for all-cause discontinuation compared to ATX and MPH. No separation in credible intervals was observed between LDX and ATX or MPH in the Bayesian analyses, and the difference was not statistically significant in frequentist analyses. ATX and MPH-ER had a lower relative risk for discontinuation due to adverse events than LDX, but there was considerable overlap in the posterior credible intervals. Due to the shapes of the posterior distributions, LDX had the highest probability of being the most tolerable treatment, followed closely by the other treatments. Furthermore, the relative risk of adverse events discontinuation for LDX was still lower than that for MPH-IR, which is currently recommended as a first-line treatment for ADHD in adults.

In conclusion, among the ADHD treatments approved for adults, LDX was observed to be consistently advantageous due to its numerically greater efficacy results and safety profile comparable to other treatments.

## Study Limitations

There are several limitations in mixed treatment comparisons via network meta-analysis. First, the review is necessarily limited by the number of prior studies available and the total sample sizes available from the prior studies. This led to the need for an imputation analysis and limited the precision of the estimates (as measured by the 95% credible intervals). Second, the systematic literature review is based on pre-specified criteria, but excluded trials could contain important information. Third, while the best effort was made to include comparable trials, the trials may have had different ways of applying inclusion/exclusion criteria; hence the trial populations may differ. Fourth, observable differences that were not incorporated into the analysis included differences in trial length and prior treatment. Longer duration trials may have larger treatment effects, but duration was not adjusted for in this analysis under the assumption that trial duration is chosen according to the drug’s duration of action by trial investigators, as different medications take different amounts of time to take effect. Prior treatment may indicate refractory patients in whom a reduced treatment effect is likely. The limited number of trials available in adults did not permit an adjustment for prior treatments. In addition, differences between the trials in patient characteristics including age, gender, baseline disease severity, unobserved baseline characteristics, and other trial level differences will not be captured in this indirect comparison due to a lack of information available to adjust for such factors. For example, cross-trial differences in length of treatment duration might limit the precision of comparison. Further research is needed to identify subgroups of patients who might benefit more from specific treatments. However, an assessment using I^2^, Cochran’s Q, and DIC indicated that heterogeneity was not present in the evidence networks for the efficacy outcomes. In addition, a comparison of the direct ATX vs. MPH treatment effect estimate with an indirect estimate formed from the ATX vs. placebo and MPH vs. placebo treatment effect estimates did not reveal the presence of inconsistency in the MPH-ATX-placebo loop in the core ADHD-RS-IV analysis. Fifth, the Bayesian method was used according to the NICE Decision Support Unit Technical Support Documents; however, the method is dependent on the choice of prior. Non-informative priors (priors that place approximately equal weight on a wide range of parameter values) were chosen so that this limitation would not influence the results. Sixth, while the imputation analysis was used to produce conservative point estimates of the relative risk of CGI-I response, the estimates of variability from that analysis do not account for the additional variability due to the imputation. Hence, while the point estimates are likely to be conservative, the corresponding credible intervals and confidence intervals should be interpreted with caution. Finally, the combined use of ADHD-RS-IV and AISRS scores should be treated with caution as the two rating scales, while comparable, are not identical (Goodman 2009).

## Conclusions

In conclusion, this systematic literature review and mixed treatment comparison provided a comparison of LDX with ATX and MPH as treatments for ADHD in adults. The findings provide evidence that LDX is an advantageous treatment for adult ADHD compared to the other treatments considered due to its numerically greater efficacy results and comparable safety results relative to other treatments. Further studies are required to strengthen the confidence in the results and compare treatments among specific subgroups of patients.

# References

1. Adler LA, Goodman DW, Kollins SH, et al. Double-blind, placebo-controlled study of the efficacy and safety of lisdexamfetamine dimesylate in adults with attention-deficit/hyperactivity disorder. J Clin Psychiatry. 2008;69(9):1364–1373.

Brent R Collett, Jeneval L Ohan, and Kathleen M Myers. Ten years review of rating scales. V: Scales assessing attention-deficit/hyperactivity disorder. J am Acad child adolescent 2003,42(9):1015-1037.

1. De Graaf R, Kessler RC, Fayyad J, et al. The prevalence and effects of adult attention-deficit/hyperactivity disorder (ADHD) on the performance of workers: results from the WHO World Mental Health Survey Initiative. Occup Environ Med. 2008;65(12):835–842. doi:10.1136/oem.2007.038448.
2. Dias, S., Welton, N. J., Sutton, A. J., et al. "NICE DSU Technical Support Document 4: Inconsistency in networks of evidence based on randomised controlled trials." NICE DSU Technical Support Document in Evidence Synthesis; No. TSD4). National Institute for Health and Clinical Excellence. 2011. http://research-information.bristol.ac.uk/files/7215354/TSD4_Inconsistency.final.08.05.12.pdf
3. Faraone SV, Biederman J, Mick E. The age-dependent decline of attention deficit hyperactivity disorder: a meta-analysis of follow-up studies. Psychol Med. 2006;36(2):159–165. doi:10.1017/S003329170500471X.
4. Fayyad J, De Graaf R, Kessler R, et al. Cross-national prevalence and correlates of adult attention-deficit hyperactivity disorder. Br J Psychiatry J Ment Sci. 2007;190:402–409. doi:10.1192/bjp.bp.106.034389.
5. Feifel D, MacDonald K. Attention-deficit/hyperactivity disorder in adults: recognition and diagnosis of this often-overlooked condition. Postgrad Med. 2008;120(3):39–47. doi:10.3810/pgm.2008.09.1906.
6. David Goodman, MD, Stephen V. Faraone, PhD, Lenard A. Adler, MD, Bryan Dirks, MD, Mohamed Hamdani, MS, and Richard Weisler, MD. Interpreting ADHD Rating Scale Scores: Linking ADHD Rating Scale Scores and CGI Levels in Two Randomized Controlled Trials of Lisdexamfetamine Dimesylate in ADHD. Primary Psychiatry. 2010;17(3):44-52.
7. Goodman DW. ADHD in Adults: Update for Clinicians on Diagnosis and Assessment. Primary Psychiatry. 2009; 16 (11): 21-30.
8. Huedo-Medina, Tania, Julio Sanchez-Meca, Fulgencio Marin-Martinez, and Juan Botella. “Assessing heterogeneity in meta-analysis:Q statistic or I2 index?” (2006). CHIP Documents. Paper 19.

<http://digitalcommons.uconn.edu/chip_docs/19>

1. Jansen JP, Fleurence R, Devine B, et al. Interpreting Indirect Treatment Comparisons and Network Meta-Analysis for Health-Care Decision Making: Report of the ISPOR Task Force on Indirect Treatment Comparisons Good Research Practices: Part 1. Value Health 2011;14:417-28.
2. Klein RG, Mannuzza S, Olazagasti MAR, et al. Clinical and functional outcome of childhood attention-deficit/hyperactivity disorder 33 years later. Arch Gen Psychiatry. 2012;69(12):1295–1303. doi:10.1001/archgenpsychiatry.2012.271.
3. Kooij SJJ, Bejerot S, Blackwell A, et al. European consensus statement on diagnosis and treatment of adult ADHD: The European Network Adult ADHD. BMC Psychiatry. 2010 Sep 3;10:67. doi:10.1186/1471-244X-10-67.
4. Matza LS, Paramore C, Prasad M. A review of the economic burden of ADHD. Cost Eff Resour Alloc CE. 2005;3:5. doi:10.1186/1478-7547-3-5.
5. McKee S. UK licenses first therapy for adults diagnosed with ADHD. PharmaTimes. http://www.pharmatimes.com/article/13-06-03/UK_licenses_first_therapy_for_adults_diagnosed_with_ADHD.aspx. Published June 3, 2013.
6. Medice. Medice receives first German authorisation for treatment of adult ADHD. 2011. Available at: http://www.medice.de/service-en/news/medice-receives-first-german-authorisation-for-treatment-of-adult-adhd.
7. National Institute for Health and Clinical Excellence. The NICE guideline on diagnosis and management of ADHD in children, young people and adults. 2009. Available at: <http://www.nice.org.uk/nicemedia/pdf/ADHDFullGuideline.pdf>.
8. National Institute for Clinical Excellence. Identifying the evidence: literature searching and evidence submission. November 30, 2012. Available at: http://publications.nice.org.uk/PMG6. Accessed on: August 8, 2013.
9. National Institute for Clinical Excellence. A general linear modelling framework for pair-wise and network meta-analysis of randomised controlled trials. NICE Decision Support Unit Technical Support Document, April 2014: http://www.nicedsu.org.uk/TSD2%20General%20meta%20analysis%20corrected%2015April2014.pdf
10. Olfson M. New options in the pharmacological management of attention-deficit/hyperactivity disorder. Am J Manag Care. 2004;10(4 Suppl):S117–124.
11. Retz W, Retz-Junginger P, Thome J, Rösler M. Pharmacological treatment of adult ADHD in Europe. World J Biol Psychiatry Off J World Fed Soc Biol Psychiatry. 2011;12 Suppl 1:89–94. doi:10.3109/15622975.2011.603229.
12. Simon V, Czobor P, Bálint S, Mészáros A, Bitter I. Prevalence and correlates of adult attention-deficit hyperactivity disorder: meta-analysis. Br J Psychiatry J Ment Sci. 2009;194(3):204–211. doi:10.1192/bjp.bp.107.048827.
13. Spencer TJ, Adler LA, Qiao M, et al. Validation of the Adult ADHD Investigator Symptom Rating Scale (AISRS). Journal of Attention Disorders. 2010; 14 (1): 57-68.
14. Weisler RH, Pandina GJ, Daly EJ, Cooper K, Gassmann-Mayer C. Randomized Clinical Study of a Histamine H3 Receptor Antagonist for the Treatment of Adults with Attention-Deficit Hyperactivity Disorder: CNS Drugs. 2012;26(5):421–434. doi:10.2165/11631990-000000000-00000.
15. Wigal T, Brams M, Gasior M, et al. Randomized, double-blind, placebo-controlled, crossover study of the efficacy and safety of lisdexamfetamine dimesylate in adults with attention-deficit/hyperactivity disorder: novel findings using a simulated adult workplace environment design. Behav Brain Funct. 2010;6(1):34. doi:10.1186/1744-9081-6-34.

### Table A-1: List of studies included in network meta-analysis

| **Author (Year)** | **Population** | **Primary Study Reference** |
| --- | --- | --- |
| Adler (2008) | 420 adults aged 18-55 years with moderate to severe ADHD | Adler LA, Goodman DW, Kollins SH, et al. Double-blind, placebo-controlled study of the efficacy and safety of lisdexamfetamine dimesylate in adults with attention-deficit/hyperactivity disorder. The Journal of clinical psychiatry. Sep 2008;69(9):1364-1373. |
| Adler (2009a) | 501 adults aged 18-54 years with ADHD | Adler LA, Spencer T, Brown TE, et al. Once-Daily Atomoxetine for Adult Attention-Deficit/Hyperactivity Disorder: A 6-Month, Double-Blind Trial. Journal of clinical psychopharmacology. February 2009; 29(1): 44-50. |
| Adler (2009b) | 226 adults aged 18-65 years with ADHD | Adler LA, Zimmerman B, Starr HL, et al. Efficacy and safety of OROS methylphenidate in adults with attention-deficit/hyperactivity disorder: a randomized, placebo-controlled, double-blind, parallel group, dose-escalation study. Journal of clinical psychopharmacology. Jun 2009;29(3):239-247. |
| Adler (2013) | 161 adults aged 18-55 years with ADHD and executive function deficits | Adler LA, Dirks B, Deas PF, et al. Lisdexamfetamine dimesylate in adults with attention-deficit/ hyperactivity disorder who report clinically significant impairment in executive function: results from a randomized, double-blind, placebo-controlled study. The Journal of clinical psychiatry. Jul 2013;74(7):694-702. |
| Biederman (2006) | 141 adults aged 19-60 years with ADHD | Biederman J, Mick E, Surman C, et al. A randomized, placebo-controlled trial of OROS methylphenidate in adults with attention-deficit/hyperactivity disorder. Biological psychiatry. May 1 2006;59(9):829-835. |
| Biederman (2010) | 223 adults in phase 1, 96 adults in phase 2, and 23 adults in phase 3 aged 19-60 years with ADHD | Biederman J, Mick E, Surman C, et al. A randomized, 3-phase, 34-week, double-blind, long-term efficacy study of osmotic-release oral system-methylphenidate in adults with attention-deficit/hyperactivity disorder. Journal of clinical psychopharmacology. Oct 2010;30(5):549-553. |
| Biederman (2012) | 61 outpatients aged 18-26 years with ADHD | Biederman J, Fried R, Hammerness P, et al. The effects of lisdexamfetamine dimesylate on the driving performance of young adults with ADHD: a randomized, double-blind, placebo-controlled study using a validated driving simulator paradigm. Journal of psychiatric research. Apr 2012;46(4):484-491. |
| Casas (2013) | 279 adults aged 18-65 years with ADHD | Casas M, Rosler M, Sandra Kooij JJ, et al. Efficacy and safety of prolonged-release OROS methylphenidate in adults with attention deficit/hyperactivity disorder: a 13-week, randomized, double-blind, placebo-controlled, fixed-dose study. The world journal of biological psychiatry : the official journal of the World Federation of Societies of Biological Psychiatry. May 2013;14(4):268-281. |
| Durell (2013) | 445 adults aged 18-30 ears with ADHD | Durell TM, Adler LA, Williams DW, et al. Atomoxetine treatment of attention-deficit/hyperactivity disorder in young adults with assessment of functional outcomes: a randomized, double-blind, placebo-controlled clinical trial. Journal of clinical psychopharmacology. Feb 2013;33(1):45-54. |
| Medori (2008) | 401 adults aged 18-63 years with ADHD | Medori R, Ramos-Quiroga JA, Casas M, et al. A randomized, placebo-controlled trial of three fixed dosages of prolonged-release OROS methylphenidate in adults with attention-deficit/hyperactivity disorder. Biological psychiatry. May 15 2008;63(10):981-989. |
| Michelson (2003) | 139 ADHD patients with mean age 40.3 years in study I placebo arm, 141 ADHD patients with mean age 40.2 in study I atomoxetine arm, 127 ADHD patients with mean age 41.2 in study II placebo arm, 129 ADHD patients with mean age 43.0 in study II atomoxetine arm. | Michelson D, Adler L, Spencer T, et al. Atomoxetine in adults with ADHD: two randomized, placebo-controlled studies. Biological psychiatry. Jan 15 2003;53(2):112-120. |
| NCT00937040 | 357 patients with mean age 35.7 years with ADHD | Adult Study / OROS Methylphenidate Hydrochloride (HCL) (OROS MPH) in Adults With Attention Deficit Hyperactivity Disorder (ADHD). NCT00937040. 2013. |
| Retz (2012) | 162 adults aged 18 years and above with ADHD | Retz W, Rosler M, Ose C, et al. Multiscale assessment of treatment efficacy in adults with ADHD: a randomized placebo-controlled, multi-centre study with extended-release methylphenidate. The world journal of biological psychiatry : the official journal of the World Federation of Societies of Biological Psychiatry. Jan 2012;13(1):48-59. |
| Rosler (2009) | 359 adults aged 18 years and above with ADHD | Rosler M, Fischer R, Ammer R, Ose C, Retz W. A randomised, placebo-controlled, 24-week, study of low-dose extended-release methylphenidate in adults with attention-deficit/hyperactivity disorder. European archives of psychiatry and clinical neuroscience. Mar 2009;259(2):120-129. |
| Sobanski (2012) | 64 patients with mean age 35.8 years with ADHD | Sobanski E, Sabljic D, Alm B, et al. A randomized, waiting list-controlled 12-week trial of atomoxetine in adults with ADHD. Pharmacopsychiatry. May 2012;45(3):100-107. |
| Spencer (1998) | 22 outpatients aged 19-60 years with ADHD | Spencer T, Biederman J, Wilens T, et al. Effectiveness and tolerability of tomoxetine in adults with attention deficit hyperactivity disorder. The American journal of psychiatry. May 1998;155(5):693-695. |
| Spencer (2005) | 146 adults aged 19-60 years with ADHD | Spencer T, Biederman J, Wilens T, et al. A large, double-blind, randomized clinical trial of methylphenidate in the treatment of adults with attention-deficit/hyperactivity disorder. Biological psychiatry. Mar 1 2005;57(5):456-463. |
| Spencer (2007) | 221 adults aged 18-60 years with ADHD and functional impairment | Spencer TJ, Adler LA, McGough JJ, et al. Efficacy and safety of dexmethylphenidate extended-release capsules in adults with attention-deficit/hyperactivity disorder. Biological psychiatry. Jun 15 2007;61(12):1380-1387. |
| Sutherland (2012) | 241 adults aged 18-60 years with ADHD | Sutherland SM, Adler LA, Chen C, Smith MD, Feltner DE. An 8-week, randomized controlled trial of atomoxetine, atomoxetine plus buspirone, or placebo in adults with ADHD. The Journal of clinical psychiatry. Apr 2012;73(4):445-450. |
| Weisler (2012) | 430 adults aged 18-55 years with ADHD | Weisler RH, Pandina GJ, Daly EJ, Cooper K, Gassmann-Mayer C, Investigators ATTS. Randomized clinical study of a histamine H3 receptor antagonist for the treatment of adults with attention-deficit hyperactivity disorder. CNS drugs. May 1 2012;26(5):421-434. |
| Young (2011) | 502 adults aged 18 years and above with ADHD | Young JL, Sarkis E, Qiao M, Wietecha L. Once-daily treatment with atomoxetine in adults with attention-deficit/hyperactivity disorder: a 24-week, randomized, double-blind, placebo-controlled trial. Clinical neuropharmacology. Mar-Apr 2011;34(2):51-60. |

### Table A-2: Study Outcomes

### Table A-3: Discontinuation Rates due to Adverse Events Reported in the Identified Trials

| **Author Year** | **No. of arms** | **Arm 1 treatment** | **Arm 1 discontinuers** | **Arm 1 sample size** | **Arm 2 treatment** | **Arm 2 discontinuers** | | **Arm 2 sample size** | **Arm 3 treatment** | **Arm 3 discontinuers** | **Arm 3 sample size** | **Arm 4 treatment** | **Arm 4 discontinuers** | **Arm 4 sample size** |
| --- | --- | --- | --- | --- | --- | --- | --- | --- | --- | --- | --- | --- | --- | --- |
| Adler 2008 | 4 | PL | 1 | 62 | LDX | | 4 | 119 | LDX | 8 | 117 | LDX | 9 | 122 |
| Adler 2013 | 2 | PL | 2 | 80 | LDX | | 6 | 79 | NA | NA | NA | NA | NA | NA |
| Rosler 2009 | 2 | PL | 10 | 118 | ERMPH | | 31 | 241 | NA | NA | NA | NA | NA | NA |
| Medori 2008 | 4 | PL | 1 | 96 | ERMPH | | 1 | 101 | ERMPH | 4 | 102 | ERMPH | 8 | 102 |
| Sobanski 2012 | 2 | PL | 0 | 37 | ATX | | 5 | 27 | NA | NA | NA | NA | NA | NA |
| Biederman 2006 | 2 | PL | 3 | 74 | ERMPH | | 9 | 67 | NA | NA | NA | NA | NA | NA |
| NCT00937040 | 2 | PL | 5 | 179 | ERMPH | | 8 | 178 | NA | NA | NA | NA | NA | NA |
| Biederman 2010 | 2 | PL | 3 | 114 | ERMPH | | 12 | 109 | NA | NA | NA | NA | NA | NA |
| Adler 2009 | 2 | PL | 6 | 116 | ERMPH | | 16 | 110 | NA | NA | NA | NA | NA | NA |
| Casas 2013 | 3 | PL | 2 | 97 | ERMPH | | 15 | 89 | ERMPH | 19 | 92 | NA | NA | NA |
| Spencer 2007 | 4 | PL | 4 | 53 | ERMPH | | 6 | 57 | ERMPH | 7 | 54 | ERMPH | 5 | 54 |
| Young 2011 | 2 | PL | 22 | 234 | ATX | | 57 | 266 | NA | NA | NA | NA | NA | NA |
| Weisler 2012 | 3 | PL | 2 | 73 | ERMPH | | 6 | 68 | ATX | 8 | 74 | NA | NA | NA |
| Sutherland 2012 | 2 | PL | 7 | 47 | ATX | | 11 | 97 | NA | NA | NA | NA | NA | NA |
| Michelson 2003 | 2 | PL | 9 | 263 | ATX | | 23 | 269 | NA | NA | NA | NA | NA | NA |
| Spencer 2005 | 2 | PL | 2 | 42 | IRMPH | | 16 | 104 | NA | NA | NA | NA | NA | NA |
| Biederman 2012 | 2 | PL | 1 | 31 | LDX | | 1 | 32 | NA | NA | NA | NA | NA | NA |
| Durell 2013 | 2 | PL | 6 | 225 | ATX | | 21 | 220 | NA | NA | NA | NA | NA | NA |

**Abbreviations:** ADHD: attention-deficit/hyperactivity disorder; ATX: atomoxetine; ER, extended release; LDX, lisdexamphetamine; MPH, methylphenidate; IR, immediate release

## Appendix B: Imputation analysis

**Step 1.**Test of normality for ADHD-RS-IV score change in SPD489-303 trial


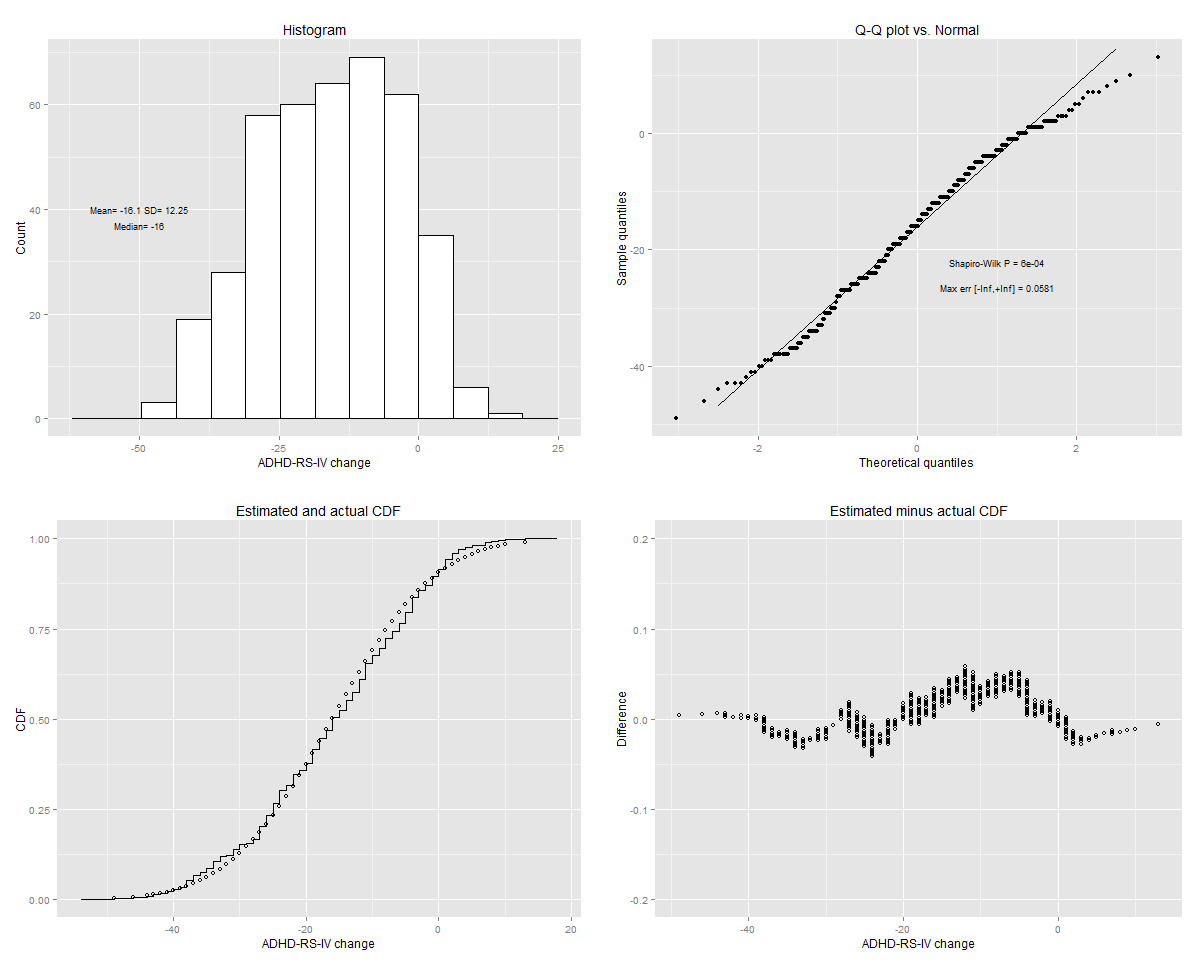


**Abbreviations**: CDF: cumulative distribution function; ADHD-RS-IV: ADHD Rating Scale-IV.

**Notes**: Normality was assessed using all ADHD-RS-IV change observations in the SPD489-303 trial (both active and placebo arms).

**Step 2.** Cut-off values based on ADHD-RS-IV for CGI-I response, using SPD489-303 trial

| **Table M-2a. Cutoffs obtained by different extrapolation methods** | | | | | |
| --- | --- | --- | --- | --- | --- |
| **Method** |  |  | **Cutoff for CGI-I response based on change in ADHD-RS-IV from baseline** | **Sensitivity^[3]^** | **Specificity^[4]^** |
| Quadratic regression (Goodman)^[1]^ | | | -13.636 | 0.905 | 0.877 |
| Direct estimation^[2]^ | |  | -14.405 | 0.885 | 0.910 |

**Notes**:

1. The quadratic regression fits a quadratic model between CGI-I and ADHD-RS-IV change from baseline, uses a cutoff of predicted CGI-I ≤ 2.5 to find the corresponding cut-off in terms of ADHD-RS-IV change. Two thirds of the observations from SPD489-303 trial (both active and placebo arms) were used.
2. The direct estimation method finds a cut-off using ADHD-RS-IV change from baseline that gives the percentage of CGI-I response corresponding to what was observed. Two thirds of the observations from SPD489-303 trial (both active and placebo arms) were used.
3. Sensitivity measures the percentage of patients achieving CGI-I response who are correctly identified as having such response.
4. Specificity measures the percentage of patients not achieving CGI-I response who are correctly identified as not having response.

**Step M-2 (continued).** Cut-off values based on ADHD-RS-IV for CGI-I response, using SPD489-303 trial

**Step 3.** Validation against the validation set of the SPD489-303 trial and other studies

| **Table M-3a. Extrapolation of SPD489-303 trial validation dataset^[3]^** | | | | | | |
| --- | --- | --- | --- | --- | --- | --- |
| **Method** |  | **Actual ADHD-RS-IV change mean (SD)** | **Actual % CGI-I response** | **Extrapolated % CGI-I response** | **Bias (Extrapolated - Actual)** |  |
| Quadratic regression (Goodman)^[1]^ | | -16.4 (11.6) | 0.563 | 0.595 | 0.032 |  |
| Direct estimation^[2]^ | | -16.4 (11.6) | 0.563 | 0.569 | 0.006 |  |

**Notes**:

1. The quadratic regression fits a quadratic model between CGI-I and ADHD-RS-IV change from baseline, uses a cut-off of predicted CGI-I ≤ 2.5 to find the corresponding cut-off in terms of ADHD-RS-IV change. Two thirds of the observations from SPD489-303 trial (both active and placebo arms) were used.
2. The direct estimation method finds a cutoff using ADHD-RS-IV change from baseline that gives the percentage of CGI-I response corresponding to what was observed. Two thirds of the observations from SPD489-303 trial (both active and placebo arms) were used.
3. The SPD489-303 trial validation dataset consisted of the remaining one third of the observations (from both active and placebo arms), which were not used to train the model.

**Step 3 (continued).** Validation against the validation set of the SPD489-303 trial and other studies

| **Table 9c. Extrapolation in trials reporting both CGI-I and ADHD-RS-IV outcomes** | | | | | | | |
| --- | --- | --- | --- | --- | --- | --- | --- |
| **Author year^[3]^** | **Arm** | **Actual ADHD-RS-IV change, mean (SD)** | **Actual % CGI-I response** | **Quadratic regression (Goodman)^[1]^** | | **Direct estimation^[2]^** | |
|  |  |  |  | **Extrapolated % CGI-I response** | **Bias (Extrapolated - Actual)** | **Extrapolated % CGI-I response** | **Bias (Extrapolated - Actual)** |
| Adler 2008 | LDX30 | -16.2 (11.6) | 0.570 | 0.588 | 0.018 | 0.562 | -0.008 |
| Adler 2008 | LDX50 | -17.4 (11.4) | 0.620 | 0.630 | 0.010 | 0.604 | -0.016 |
| Adler 2008 | LDX70 | -18.6 (11.4) | 0.610 | 0.669 | 0.059 | 0.644 | 0.034 |
| Adler 2008 | Placebo | -8.2 (11.3) | 0.290 | 0.315 | 0.025 | 0.291 | 0.001 |
| Adler 2013 | LDX | -21.4 (12.0) | 0.696 | 0.741 | 0.045 | 0.720 | 0.024 |
| Adler 2013 | Placebo | -10.3 (12.3) | 0.293 | 0.393 | 0.100 | 0.370 | 0.076 |
| Spencer 2007 | d-MPH-ER20 | -13.7 (9.0) | 0.474 | 0.503 | 0.029 | 0.469 | -0.005 |
| Spencer 2007 | d-MPH-ER30 | -13.4 (9.4) | 0.370 | 0.490 | 0.120 | 0.458 | 0.088 |
| Spencer 2007 | d-MPH-ER40 | -16.9 (9.4) | 0.556 | 0.636 | 0.080 | 0.604 | 0.048 |
| Spencer 2007 | Placebo | -7.9 (10.2) | 0.264 | 0.287 | 0.023 | 0.262 | -0.002 |
| SPD489-317 CSR | ATX | -19.4 (12.9) | 0.636 | 0.673 | 0.037 | 0.651 | 0.015 |
| SPD489-317 CSR | LDX | -26.2 (11.9) | 0.817 | 0.854 | 0.037 | 0.839 | 0.021 |

| **Extrapolation in trials reporting both CGI-I and AISRS outcomes** | | | | | | | |
| --- | --- | --- | --- | --- | --- | --- | --- |
| **Author year^[3]^** | **Arm** | **Actual AISRS change, mean (SD)** | **Actual % CGI-I response** | **Quadratic regression (Goodman)^[1]^** | | **Direct estimation^[2]^** | |
|  |  |  |  | **Extrapolated % CGI-I response** | **Bias (Extrapolated - Actual)** | **Extrapolated % CGI-I response** | **Bias (Extrapolated - Actual)** |
| Biederman 2006 | MPH-OROS | -18.8 (9.4) | 0.687 | 0.708 | 0.022 | 0.680 | -0.007 |
| Biederman 2006 | Placebo | -12.9 (9.6) | 0.405 | 0.470 | 0.065 | 0.439 | 0.033 |

**Notes**:

1. The linear regression fits a quadratic model between CGI-I and ADHD-RS-IV change from baseline, uses a cut-off of predicted CGI-I ≤ 2.5 to find the corresponding cut-off in terms of ADHD-RS-IV change. Two thirds of the observations from SPD489-303 trial (both active and placebo arms) were used.
2. The direct estimation method finds a cutoff using ADHD-RS-IV change from baseline that gives the percentage of CGI-I response corresponding to what was observed. Two thirds of the observations from SPD489-303 trial (both active and placebo arms) were used.
3. The Adler 2008 study corresponds to the SPD489-303 trial, and the Adler 2013 study corresponds to the SPD489-403 trial. SPD489-317 is an adolescent trial.

**Step 4.** Prediction of CGI-I response

| **Table M-4. Impute CGI-I response for ATX based on ADHD-RS-IV change** | | | | | | | | |
| --- | --- | --- | --- | --- | --- | --- | --- | --- |
| **AGID** | **Author year** | **Arm** | **Actual Change in ADHD-RS-IV (mean)** | **Actual Change in ADHD-RS-IV (SD)** | **Extrapolated % CGI-I response, quadratic regression (Goodman)** | **r / n** | **Extrapolated % CGI-I response, direct estimations** | **r / n** |
| F25 | Weisler 2012 | ATX | -15.300 | 14.520 | 54.6% | 40/73 | 52.5% | 38/73 |
| F25 | Weisler 2012 | Placebo | -8.800 | 11.740 | 34.0% | 25/73 | 31.7% | 23/73 |
|  | Relative risk (ATX vs. placebo): | | |  | 1.60 |  | 1.66 |  |
|  | Odds ratio (ATX vs. placebo): | | |  | 2.33 |  | 2.38 |  |

**Table 4, continued**

| **Impute CGI-I response for ATX based on AISRS change** | | | | | | | | |
| --- | --- | --- | --- | --- | --- | --- | --- | --- |
| **AGID** | **Author year** | **Arm** | **Actual Change in AISRS (mean)** | **Actual Change in AISRS (SD)** | **Extrapolated % CGI-I response, quadratic regression (Goodman)** | **r / n** | **Extrapolated % CGI-I response, direct estimations** | **r / n** |
| B525 | Adler 2009a | ATX | -16.47 | 18.46 | 56.1% | 140/250 | 54.5% | 136/250 |
| B525 | Adler 2009a | Placebo | -11.61 | 17.49 | 45.4% | 114/251 | 43.7% | 110/251 |
|  | Relative risk (ATX vs. placebo): | | |  | 1.24 |  | 1.25 |  |
|  | Odds ratio (ATX vs. placebo): | | |  | 1.54 |  | 1.54 |  |
| B198 | Sutherland 2012 | ATX | -17.00 | 13.20 | 60.1% | 58/97 | 57.8% | 56/97 |
| B198 | Sutherland 2012 | Placebo | -12.39 | 16.36 | 47.0% | 22/47 | 45.1% | 21/47 |
|  | Relative risk (ATX vs. placebo): | | |  | 1.28 |  | 1.28 |  |
|  | Odds ratio (ATX vs. placebo): | | |  | 1.70 |  | 1.67 |  |
| B251 | Young 2011 | ATX | -13.7 | 12.5 | 50.2% | 135/268 | 47.8% | 128/268 |
| B251 | Young 2011 | Placebo | -8.0 | 11.0 | 30.4% | 71/234 | 28.0% | 66/234 |
|  | Relative risk (ATX vs. placebo): | | |  | 1.65 |  | 1.70 |  |
|  | Odds ratio (ATX vs. placebo): | | |  | 2.31 |  | 2.35 |  |

1. MPH-IR was not included in the network because no studies with an MPH-IR arm reported ADHD-RS-IV change. [↑](#footnote-ref-1)
2. MPH-IR was not included in the network because no studies with an MPH-IR arm reported ADHD-RS-IV or AISRS change. [↑](#footnote-ref-2)
3. MPH-IR and ATX were not included in the network because no studies with MPH-IR or ATX arms reported CGI-I response. [↑](#footnote-ref-3)
4. The placebo risk is the pooled risk of response (CGI-I = 1 or 2) of the placebo arms in the data. The placebo risk uncertainty is measured as the 95% confidence interval around this pooled placebo risk. [↑](#footnote-ref-4)
5. The placebo risk is the pooled risk of discontinuation of the placebo arms in the data. The placebo risk uncertainty is measured as the 95% confidence interval around this pooled placebo risk. [↑](#footnote-ref-5)
6. The placebo risk is the pooled risk of discontinuation of the placebo arms in the data. The placebo risk uncertainty is measured as the 95% confidence interval around this pooled placebo risk. [↑](#footnote-ref-6)
7. The placebo risk is the pooled risk of response (CGI-I = 1 or 2) of the placebo arms in the data. The placebo risk uncertainty is measured as the 95% confidence interval around this pooled placebo risk. [↑](#footnote-ref-7)
8. The placebo risk is the pooled risk of response (CGI-I = 1 or 2) of the placebo arms in the data. The placebo risk uncertainty is measured as the 95% confidence interval around this pooled placebo risk. [↑](#footnote-ref-8)
9. The placebo risk is the same as that for the corresponding Bayesian analysis, shown in Table 10. [↑](#footnote-ref-9)
10. The placebo risk is the same as that for the corresponding Bayesian analysis, shown in Table 11. [↑](#footnote-ref-10)
11. The placebo risk is the pooled risk of response (CGI-I = 1 or 2) of the placebo arms in the data. The placebo risk uncertainty is measured as the 95% confidence interval around this pooled placebo risk. [↑](#footnote-ref-11)
12. The placebo risk is the pooled risk of response (CGI-I = 1 or 2) of the placebo arms in the data. The placebo risk uncertainty is measured as the 95% confidence interval around this pooled placebo risk. [↑](#footnote-ref-12)
13. The placebo risk is the pooled risk of response (CGI-I = 1 or 2) of the placebo arms in the data. The placebo risk uncertainty is measured as the 95% confidence interval around this pooled placebo risk. [↑](#footnote-ref-13)
14. The placebo risk is the pooled risk of response (CGI-I = 1 or 2) of the placebo arms in the data. The placebo risk uncertainty is measured as the 95% confidence interval around this pooled placebo risk. [↑](#footnote-ref-14)
15. The placebo risk is the same as that for the corresponding Bayesian analysis, shown in Table 14. [↑](#footnote-ref-15)
16. The placebo risk is the same as that for the corresponding Bayesian analysis, shown in Table 15. [↑](#footnote-ref-16)
17. The placebo risk is the same as that for the corresponding Bayesian analysis, shown in Table 16. [↑](#footnote-ref-17)
18. The placebo risk is the same as that for the corresponding Bayesian analysis, shown in Table 17. [↑](#footnote-ref-18)
