# Supplementary material for: A cost-effectiveness analysis of lisdexamfetamine dimesylate in the treatment of adults with attention-deficit/hyperactivity disorder in the UK
Source: Eur J Health Econ. 2017 Jan 16;19(1):21–35. doi: 10.1007/s10198-016-0864-4 (PMC5773633; doi:10.1007/s10198-016-0864-4)
Supplement: Supplementary file 2 — Supplementary material 2 (DOCX 553 kb) [file 10198_2016_864_MOESM2_ESM.docx]

**Supplementary Material: Systematic Review of Health Economics Literature in Adult ADHD**

**26 June 2014**

Article title: A cost-effectiveness analysis of lisdexamfetamine dimesylate in the treatment of adults with attention-deficit/hyperactivity disorder in the United Kingdom

Journal: The European Journal of Health Economics

Authors: Evelina A Zimovetz, Alain Joseph, Rajeev Ayyagari, Josephine A Mauskopf

Corresponding author: Evelina Zimovetz, RTI Health Solutions; ezimovetz@rti.org

# objectives

The objective of this work was to perform a systematic review of economic evidence relevant to the development of an economic model of pharmacological treatments for adults with attention deficit hyperactivity disorder (ADHD) in the United Kingdom (UK). The specific requirements of the review were set out by the National Institute for Health and Care Excellence (NICE) in its *Methods Guide* (NICE, 2013) as follows:

- To identify published economic analyses of lisdexamfetamine (LDX) for the treatment of adults with ADHD and to prepare a summary and critical appraisal of these evaluations (if any)^[[1]](#footnote-1)^
- To identify utility, resource use, and direct and indirect cost estimates in adult ADHD specific to the UK
- To prepare summaries of the data reported in the identified studies in a format suitable for inclusion in the economic model

# METHODS OF REVIEW

## Search Strategy for Identification of Studies

The systematic literature review was performed in accordance with a prespecified protocol. Searches were conducted via electronic medical databases and specified websites. The details of the protocol are provided in the following subsections.

### Electronic Databases

The following electronic databases were searched on 17 January 2014:

- MEDLINE (using PubMed platform)
- MEDLINE In-Process (using PubMed platform)
- EconLit (using Dialog Platform)
- Embase (using Dialog Platform)
- BIOSIS (using Dialog Platform)
- The Cochrane Library (using the Wiley platform), including the following:
- The National Health Service’s Economic Evaluation Database
- Health Technology Assessment database

### Additional Sources

In addition to the electronic databases, the following additional sources were searched for relevant articles:

- The searches of the following conference websites were conducted:
- International Society for Pharmacoeconomics and Outcomes Research: <http://www.ispor.org/research_study_digest/research_index.asp>
- The American Psychiatric Association (proceedings of the meeting): <http://www.psych.org/MainMenu/EducationCareerDevelopment/Library/AbstractsAnnualMeetingandInstitute.aspx>
- The ADHD World Federation (proceedings of the latest World Congress): <http://www.adhd-federation.org/fileadmin/user_upload/Congress_2013/Final_2013/V3.Final_Programme_ADHD2013.pdf>
- The Cost-Effectiveness Analysis Registry (to identify utility estimates)
  <https://research.tufts-nemc.org/cear4/default.aspx>
- NICE: <http://www.nice.org.uk/>
- The Clinicaltrial.gov: <http://clinicaltrials.gov/>

### Date and Language Search Limits

Electronic database searches encompassed articles that have been published since 2000. Searches of conference proceedings were limited to abstracts published in 2012 and 2013.^[[2]](#footnote-2)^ Only English-language publications were included in the review.

### Search Terms

Search terms included combinations of free text and Medical Subject Headings (MeSH). The search strategy was designed to identify economic evaluations of LDX and any studies in adults with ADHD that reported resource use, cost, or utility estimates by clinical response.^[[3]](#footnote-3)^ The following types of search terms were used:

- **Population of interest:** Terms for adult ADHD (e.g., “Attention deficit hyperactivity disorder”[MeSH], “hyperkinetic disorder”)^[[4]](#footnote-4)^
- **Study types of interest:**
- Economic evaluations, including cost-effectiveness, cost-utility, cost-minimisation, and cost-benefit analyses using economic models or analyses alongside clinical trials^[[5]](#footnote-5)^
- Cost and resource use studies (e.g., “Cost of Illness”[MeSH], “Costs and Cost Analysis”[MeSH], “Economics, medical”[MeSH], “Health Care Costs”[MeSH], “resource use”)
- Utility studies (e.g., “quality of life”, “QOL”, “health utility”, “standard gamble”, “time trade off”, “EuroQol”, “quality of well-being”, “HUI”, “SF-6D”, “QALY”)
- **Exclusionary terms:** Unwanted publication types, comments, editorials, letters, case reports, and studies in animals

Appendix A presents the specific search strategies used. ‎Table A-1 presents the MEDLINE search strategy. This search strategy was adapted to search other electronic databases, and these specific search strategies are presented in ‎Table A-2 to ‎Table A-5, also in Appendix A.

Terms for searches performed on the conference websites were drawn from the listings in ‎Table A-1 as appropriate for the search features of individual sites. A log of these searches was maintained and is presented in Appendix B (‎Table B-1).

## Inclusion and Exclusion Criteria

The inclusion and exclusion criteria were based on a strategy to identify study types of interest within the population of interest. The criteria listed in ‎Table 1 were applied during a study selection process.

1. Criteria for the Inclusion and Exclusion of Studies

| Criteria | Included | Excluded |
| --- | --- | --- |
| Economic evaluations of LDX | - Population: Adults with ADHD - Intervention: LDX - Study type: Economic evaluations, including studies based on models, cost analyses performed alongside clinical trials, and budget-impact analyses - Outcomes: Cost-effectiveness results for LDX in ADHD, e.g., cost per QALY, per-patient costs, QALYs gained | - Population: Children and adolescents with ADHD - Interventions: Economic evaluations of other interventions in ADHD that do not investigate LDX - Study type: Retrospective observational studies, reviews, letters, comment articles, or any sources that discuss costs but where no formal economic analysis has been undertaken; general cost-of-illness or economic-burden studies that do not estimate incremental cost-effectiveness or cost-utility ratios for LDX |
| Utility studies | - Population: Adults with ADHD - Study type: Studies reporting utility estimates or preference weights, e.g., utility studies, economic evaluations - Outcomes: Utility or preference weights by clinical response or disease severity | - Population: Children and adolescents with ADHD - Study type: Any non-primary sources of utility weights; methodological studies - Outcomes: Value of health effects measured by disease-specific instruments; utility or preference weights not provided by clinical response or disease severity |
| Cost studies | - Population: Adults with ADHD - Study type: Studies reporting costs and resource utilisation, e.g., clinical trials or other prospective or cross-sectional studies, economic evaluations - Outcomes: Direct medical costs, resource utilisation, indirect costs and work productivity losses associated with ADHD, reported by clinical response or disease severity | - Population: Children and adolescents with ADHD - Study type: Any non-primary source of cost or resource use data - Outcomes: Aggregated national estimates of ADHD costs; costs not provided by clinical response or disease severity |

ADHD = attention-deficit hyperactivity disorder; LDX = lisdexamfetamine; QALY = quality-adjusted life-year.

## Study Selection Process

For a study to be considered in the review, the study had to meet the inclusion criteria listed in ‎Table 1. The study selection process occurred in the following two phases:

- Level 1 screening: Titles and abstracts of identified studies were reviewed by one researcher to determine eligibility according to the inclusion and exclusion criteria presented in ‎Table 1.
- Level 2 screening: Full texts of studies selected at level 1 were obtained and reviewed by one researcher to determine eligibility using the inclusion and exclusion criteria presented in ‎Table 1.

The inclusion and exclusion processes were thoroughly documented, including completion of a modified Preferred Reporting of Systematic Reviews and Meta-Analysis flow chart (Moher et al., 2009). For each study excluded at level 1, the reason for exclusion was recorded. Similarly, for each study excluded at level 2, the study citation was tabulated and the reason for exclusion recorded. See ‎Table C-1 in Appendix C for a list of publications removed at level 2 screening. Relevant studies were advanced to the formal data-extraction and quality-assessment process.

## Data Extraction and Quality Assessment

### Economic Evaluations of LDX

No data extraction or quality assessment was undertaken as the review did not identify any economic evaluation of LDX in the treatment of adults with ADHD.^[[6]](#footnote-6)^

### Utility Studies

The data fields used for the extraction were in accordance with the requirements of the NICE’s single technology appraisal template, Section 7.4.6 (NICE, 2012). For studies in which health-related quality of life is measured, the following details were required to be provided:

- Population in which health effects were measured
- Information on recruitment
- Interventions and comparators
- Sample size
- Response rates
- Description of health states
- Adverse events
- Appropriateness of health states, given condition, and treatment pathway
- Method of elicitation
- Method of valuation
- Mapping
- Uncertainty around values
- Consistency with reference case
- Appropriateness for cost-effectiveness analysis
- Results with confidence intervals

To appraise the quality of the included studies, their methodological compliance with the NICE reference case was assessed.

### Resource Use and Cost Studies

No data extraction or quality assessment were undertaken for studies reporting resource use or cost estimates because no appropriate studies were identified in adults with ADHD.^[[7]](#footnote-7)^ The data extraction was performed on one study reporting data relating to productivity. The following data fields were used:

- Country of study
- Description of available data
- Details of methods
- Suitability to economic analysis
- Productivity-related estimate(s)

## Quality Control

Quality-control procedures for inclusion and exclusion of articles included the following:

- A random selection (10%) of all studies screened at level 1 was checked by a second researcher. There were a few errors identified in this sample; as a result, the second reviewer screened all titles independently. The identified discrepancies were discussed by the two researchers and agreed upon by consensus.
- A second researcher reviewed all studies selected after the level 2 screen, to confirm the studies’ eligibility. The second reviewer also screened all of the studies included in the level 2 screen. There were no discrepancies identified.

Resources obtained via the World Wide Web were printed to maintain a record of information in case the electronic source changed or was removed. A second researcher verified all extracted data with their original sources.

# LITERATURE SEARCH RESULTS

## Electronic Databases

The database searches using the predefined search strategy yielded a total of 1535 titles (PubMed = 638, Embase = 636, EconLit = 2, Cochrane = 118, BIOSIS = 141), of which 400 records were duplicates (‎Table 2). Hence, 1135 titles or abstracts were eligible for screening. The searches were performed on 17 January 2014 and were limited to studies published from 2000 or later. The titles and abstracts identified were exported from Reference Manager into a Microsoft Excel file for screening purposes.

1. Search Results, by Database

| Database | Records | Unique Records | Range in Reference Manager |
| --- | --- | --- | --- |
| PubMed/MEDLINE | 638 | 638 | 1-638 |
| Embase | 636 | 407 | 638-1045 |
| EconLit | 2 | 0 | N/A |
| Cochrane | 118 | 53 | 1046-1098 |
| BIOSIS | 141 | 37 | 1099-1135 |
| Totals | **1535** | **1135** |  |

N/A = not applicable.

## Hand Searches

The searches identified three systematic literature reviews, two on quality-of-life assessments in adults with ADHD (Marfatia et al., 2011; Péntek et al., 2012) and one on economic aspects of medical treatment of ADHD in adults (Benkert et al., 2011). However, the reference lists of these reviews could not be searches for relevant studies because the publications were available as abstracts only. The review by Péntek and colleagues (2012) did present a reference to a utility study (referenced in the abstract as “Patient 2011;4(4):247-57”). This appears to be a publication by Lloyd and colleagues (2011), which has already been identified by the current review. The search of the NICE website for relevant guidelines retrieved one guideline (‎Table A-1, Appendix A), which was hand-searched for any relevant publications. One study by Laing and Aristides (2005) reporting utility values in adults with ADHD was identified as potentially relevant and included in the screening.

## Screening Process and Results

A total of 1245 records (titles and abstracts) were selected for manual screening (databases = 1135, Internet searches = 109, hand searches = 1) to identify all relevant studies that met the predefined inclusion or exclusion criteria presented in the protocol. Titles and abstracts of studies identified from the electronic database searches and Internet searches were reviewed by two researchers. Any differences were resolved by consensus.

After the initial screening of titles and abstracts (level 1 screening), 33 publications (databases searches = 30, Internet searches = 2, hand searches = 1) were progressed for screening of the full text (level 2 screening). Studies were reviewed for eligibility by two researchers. At the level 2 screening, 4 articles were included for data extraction (database searches = 2, Internet searches = 1, hand searches = 1). The inclusion and exclusion processes were documented, including completion of a Preferred Reporting Items for Systematic Reviews and Meta-analyses flow chart (‎Figure 1). Abstracts of all included studies are presented in Section ‎4. ‎Table 3 presents references of studies excluded after level 2 screening, with reasons for exclusion.

1. PRISMA Diagram for Study Inclusion and Exclusion


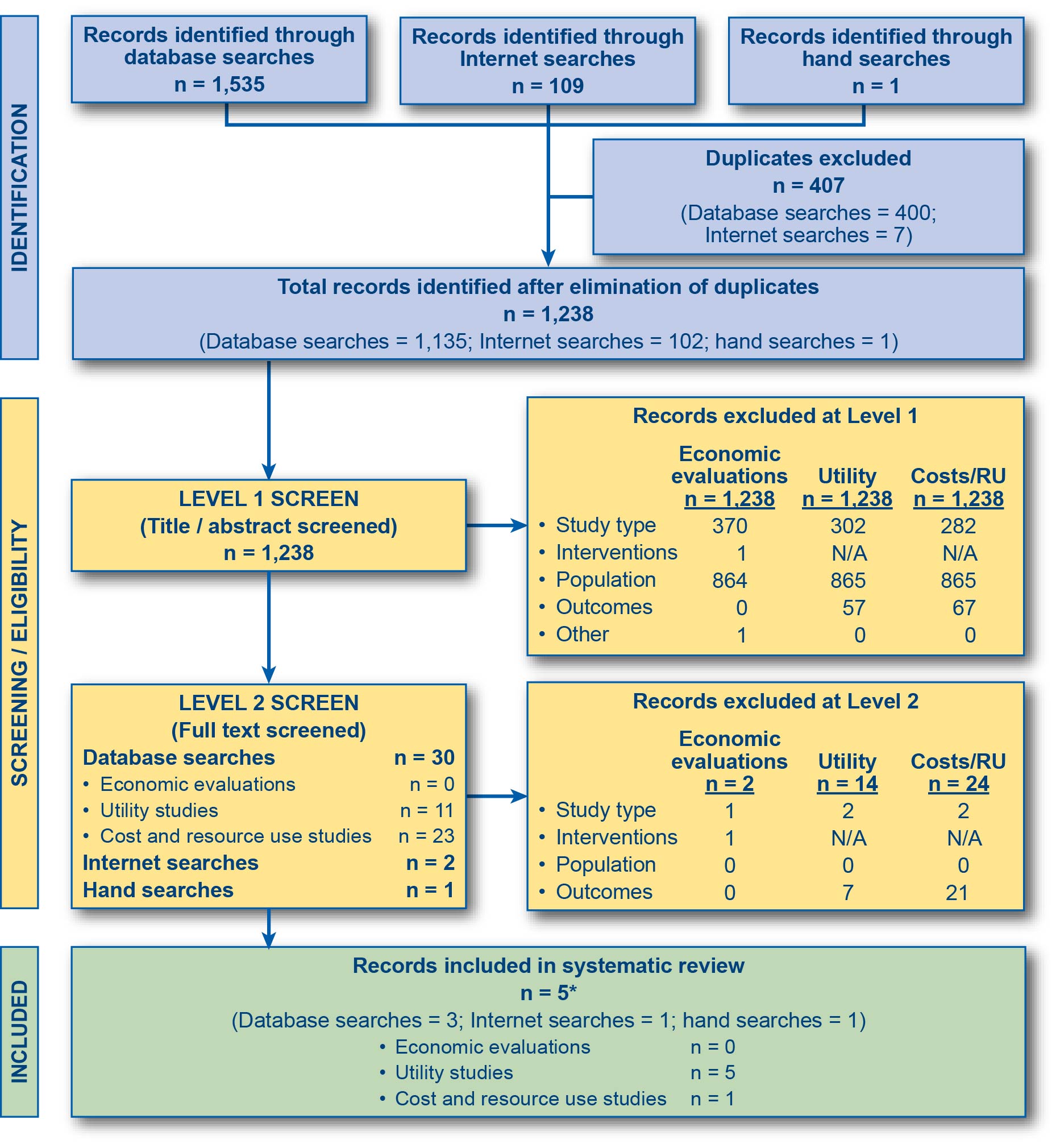


N/A = not applicable; PRISMA = Preferred Reporting of Systematic Reviews and Meta-Analysis; RU = resource use.

* One study (Mitsi et al., 2010) was included in two categories because it reported both the utility values and data related to productivity losses.

# INCLUDED STUDIES

A total of five studies were included in the review. One study was included in both the utility and cost reviews (Mitsi et al., 2010).

## Studies Reporting Utility Estimates

‎Table 3 presents a summary of the included studies and their potential appropriateness for the economic analysis. The appropriateness of health states adopted by the utility studies was evaluated both in terms of the therapeutic area, with its established treatment pathway, and in terms of the suitability for the economic analysis. Our economic analysis defines the modelled health states as response and non-response. ‎Table 4 evaluates the reported utility estimates in relation to the NICE reference case.^[[8]](#footnote-8)^ The final selection of the studies for inclusion in the economic model was based on the compatibility of the definitions of the health states used with the definitions used in our economic model, as well as the study’s compliance with the NICE reference case.

## Studies Reporting Resource Use, Cost, and Productivity Estimates

The majority of studies screened at the level 2 screen reported health care resource costs or productivity losses for adults with ADHD and not by clinical response or disease severity. Ten studies reported health care resource costs and/or productivity losses for adults with ADHD versus adults without ADHD. For this reason, these studies did not meet the inclusion criteria and therefore were excluded; however, their references are listed in ‎Table D-1, Appendix D for information. The estimates reported in these studies could potentially be used in validating the estimates selected for inclusion in the model (‎Table E-1, Appendix E). ‎Table 5 presents methods and data from the included study reporting the employment rates and work impairment for controlled versus uncontrolled ADHD patients.

1. Summary of Identified Studies Reporting Utility Weights

| First Author (Year) | Study Population | Methods of Elicitation and Valuation | Health-State Description | Utility Estimates (SD) | Appropriateness of Health States |
| --- | --- | --- | --- | --- | --- |
| Haynes (2013) abstract | N = 158  Mean age = 47 years  49.4% female | Health-state vignettes were drafted based on literature review, interviews with 4 clinicians, and clinical trial data. The health states were revised based on a pilot study with 26 participants. Final health states were rated in TTO interviews with general population. | Responders  Non-responders  Untreated patients | 0.82 (0.17)  0.68 (0.28)  0.67 (0.28) | **To condition and treatment pathways:**  Health states appear appropriate; however, no clinical definition of response was provided  **To economic analysis:**  Health states are appropriate for use in the economic analysis |
| Péntek (2012) abstract | N = 44  Mean age = 30 years  79.5% male  Disease duration of 7.6 years | A cross-sectional survey in an outpatient psychiatry centre was performed and the CGI-S scale was used to categorise patients by severity. | Mild (CGI-S = 3)  Moderate (CGI-S = 4) | 0.799  0.706 | **To condition and treatment pathways:**  Health states appear appropriate, with clinical definitions provided  **To economic analysis:**  Health states are not appropriate for use in the economic analysis because these are not represented by the dichotomous response and non-response states |
| Lloyd (2011)  full text | N = 100  Mean age = 38.5 years  53% female | Detailed qualitative interview data were collected from 20 young ADHD patients to characterise their HRQOL. In addition, item-by-item clinical and HRQOL data from a clinical trial were used to define and describe 4 health states (largely based on the CGI-S states). ADHD experts assessed the content validity of the descriptions. The states were rated by 100 members of the UK general public, using the TTO interview and visual analog scale. | Normal  Borderline to mildly ill  Moderately to markedly ill  Severely ill | 0.839 (0.203)  0.787 (0.217)  0.578 (0.275)  0.444 (0.230) | **To condition and treatment pathways:**  Health states appear appropriate, with clinical definitions provided  **To economic analysis:**  Health states are potentially appropriate for use in the economic analysis, although these are not represented by the dichotomous response and non-response states. |
| Mitsi (2010)  abstract | N = 174  Mean age = 35 years  56% male  43% diagnosed with ADHD in adulthood | An IRB-approved, web-based survey was administered to evaluate the impact of ADHD in pre-identified Canadian subjects with ADHD. EQ-5D was administered as part of a 28-item questionnaire. | ADHD group with self-reported controlled symptoms  ADHD group with self-reported uncontrolled symptoms | 0.83 (US)  0.78 (UK)  0.76 (US)  0.68 (UK) | **To condition and treatment pathways:**  Health states appear appropriate; however, no clinical definition of “controlled” was provided  **To economic analysis:**  Health states are appropriate for use in the economic analysis |
| Laing (2005) abstract | N = 218  Mean age = NR  % male = NR | To derive utilities, the study used data from a 13-week randomised trial of atomoxetine. SF-36 scores were converted into a QALY value using SF-6D for key health states; responder and non-responders without or with adverse events (grouping defined as per clinical trial). The method for conversion was based on previously published one (Brazier et al., 1998). | Non-responders  Responders without adverse events  Responders with adverse events | 0.630  0.682  0.671 | **To condition and treatment pathways:**  Health states appear appropriate; however, no clinical definition of response was provided  **To economic analysis:**  Health states are appropriate for use in the economic analysis |

ADHD = attention-deficit hyperactivity disorder; CGI-S = Clinical Global Impression-Severity; EQ-5D = EuroQol 5 Dimensions; HRQOL = health-related quality of life; IRB = institutional review board; NR = not reported; QALY = quality-adjusted life-year; SD = standard deviation; SF-36 = SF-36 Health Survey; SF-6D = SF-6D Health Survey; TTO = time trade-off; UK = United Kingdom; US = United States.

1. Compliance of Utility Estimates With NICE Reference Case

| First Author (Year) | Reported Directly From Patients? | Values = Public Preferences Using Choice-Based Method? | EQ-5D? | Utility Scale?^a^ |
| --- | --- | --- | --- | --- |
| Haynes (2013) abstract | No | Yes, public preferences using TTO | No, health state vignettes | Yes |
| Péntek (2012) abstract | Yes | Yes (tariff not reported) | Yes | Yes |
| Lloyd (2011) full text | No | Yes, public preferences using TTO | No, health state vignettes | Yes |
| Mitsi (2010) abstract | Yes | Yes (presumably UK tariff) | Yes | Yes |
| Laing (2005) abstract | Yes | Yes, public preferences using SG converted from SF-36 | No, SF-6D | Yes |

EQ-5D = EuroQol 5 Dimensions; NICE = National Institute for Health and Care Excellence; SF-36 = SF-36 Health Survey; SF-6D = SF-6D Health Survey; SG = standard gamble; TTO = time trade-off; UK = United Kingdom.

Note: The header row represents the requirements of the NICE reference case, which states that measurement of changes in health-related quality of life should be reported directly by patients, and the value of changes in patients’ health-related quality of life should be based on public preferences using a choice-based method. The EQ-5D is the preferred measure of health-related quality of life in adults (NICE, 2013).

^a^ 1 = full or perfect health; 0 = dead.

1. Summary of Identified Studies Reporting Resource Use, Cost, and Productivity

| Author, Country | Description of Available Data | Details of Methods | Suitability to Economic Analysis | Reported Estimates |
| --- | --- | --- | --- | --- |
| Mitsi, 2010  Canada | Employment rates and work impairment | An IRB-approved, wed-based survey was administered to evaluate the impact of ADHD in pre-identified Canadian subjects with ADHD | The study estimated employment rates and work impairment separately for controlled and uncontrolled patients, which allows for these estimates to be incorporated in the model using controlled and uncontrolled as proxies for response and non-response, respectively | Employment rate: 59% (uncontrolled) 74% (controlled)  Number of hours worked in past 7 days:   - Controlled ADHD patients: 29.5 (19.3) - Uncontrolled ADHD patients: 31.5 (14.0) (*P* = 0.0559)   Number of hours missed from work due to ADHD-related reasons:   - Controlled: 2.6 (7.8) - Uncontrolled: 2.7 (7.7) (*P* = 0.9883)   Number of hours missed from work due to other reasons:   - Controlled: 2.6 (6.3) - Uncontrolled: 4.7 (10.8) (*P* = 0.2421)   Extent to which ADHD impacted productivity while working (0-10):   - Controlled: 2.1 (2.2) - Uncontrolled: 4.2 (2.2) (*P* < 0.0001) |

ADHD = attention-deficit hyperactivity disorder; IRB = institutional review board.

# CONCLUSIONS

A total of five studies were included in the systematic review. All of the studies reported utility values in adults with ADHD by clinical response or disease severity, and one of the studies also reported employment rates and work impairment for controlled versus uncontrolled ADHD patients. The review identified no economic evaluations of LDX for the treatment of adults with ADHD, confirming the need for a de novo analysis, which is currently under development. There were no studies identified that report cost and resource use estimates by clinical response or disease severity in adults with ADHD.

The review identified a number of potentially relevant sources of utility data for use in the economic analysis of LDX. All but one of these publications were presented in a form of an abstract, which limited the amount of information available on the methodologies employed by the studies. Only two of the included studies (Péntek et al., 2012; Mitsi et al., 2010) complied with the NICE reference case, which states that measurement of changes in health-related quality of life should be reported directly by patients, that the value of changes in patients’ health-related quality of life should be based on public preferences using a choice-based method, and that the EQ-5D is the preferred measure of health-related quality of life in adults (NICE, 2013). However, these studies did not explicitly report whether the UK tariff was used. Two studies (Haynes et al., 2013; Lloyd et al., 2011) used health-state vignettes, which is not a recommended method for capturing utilities, according to NICE (NICE, 2013). One study (Laing and Aristides, 2005) used general population values to generate a preference-based single index measure for health from SF-36 Health Survey scores collected in a clinical trial.

The utility estimates for responders were represented in the literature by a number of health states, including responders, mild disease, normal, borderline to mildly ill, and controlled symptoms. These estimates ranged between 0.839 (normal) and 0.671 (responders with adverse events). Similarly, the non-responder utilities were represented by a number of health states: non-responders, moderate disease, moderate to markedly ill, severely ill, and uncontrolled symptoms. The utility estimates associated with non-response ranged between 0.76 (uncontrolled symptoms) and 0.444 (severely ill).

Our review included only English-language studies; therefore, the review potentially could have missed relevant information reported in non-English languages. However, despite this limitation, the study provides a comprehensive review of input parameter values required for the development of a cost-effectiveness model evaluating treatments in adult patients with ADHD.

The review highlighted the data gap in cost and resource use estimates appropriate for inclusion in economic analyses evaluating treatments of adult patients with ADHD.

# References

American Psychiatric Association. Diagnostic and statistical manual of mental disorders, 5th ed. Washington DC: American Psychiatric Association; 2013.

Barkley RA, Fischer M, Smallish L, Fletcher K. The persistence of attention-deficit/hyperactivity disorder into young adulthood as a function of reporting source and definition of disorder. J Abnorm Psychol. 2002;111:279-89. [PubMed: 12003449]

Benkert D, Krause KH, Wasem J, Aidelsburger P. The economic aspects of medical treatment of Attention-Deficit/Hyperactivity Disorder (ADHD) in adults—a systematic review. J Ment Health Policy Econ. 2011;14:S3-4.

Brazier J1, Usherwood T, Harper R, Thomas K. Deriving a preference-based single index from the UK SF-36 Health Survey. J Clin Epidemiol. 1998 Nov;51(11):1115-28.

Doshi JA, Hodgkins P, Kahle J, Sikirica V, Cangelosi MJ, Setyawan J, et al. Economic impact of childhood and adult attention-deficit/hyperactivity disorder in the United States. J Am Acad Child Adolesc Psychiatry. 2012 Oct, 51(10):990-1002.

Dulcan M. Practice parameters for the assessment and treatment of children, adolescents, and adults with attention-deficit/hyperactivity disorder. J Am Acad Child Adolesc Psychiatry. 1997;36(suppl 10):85S-121S. [PubMed: 9334567]

Faraone SV, Biederman J, Mick E. The age-dependent decline of attention deficit hyperactivity disorder: a meta-analysis of follow-up studies. Psychol Med. 2006;36:159-65. [PubMed: 16420712]

Haynes V, Matza LS, Devine MK, Davies E, Kostelec J, Televantou F, et al. Health state utilities associated with adult ADHD. Poster presented at the 4th World Congress on ADHD; June 6-9, 2013. Milan, Italy.

Kessler RC, Adler LA, Barkley R, Biederman J, Conners CK, Faraone SV, et al. Patterns and predictors of attention-deficit/hyperactivity disorder persistence into adulthood: results from the national comorbidity survey replication. Biol Psychiatry. 2005;57:1442-51. [PMCID: PMC2847347] [PubMed: 15950019]

Kooij S, Bejerot S, Blackwell A. European consensus statement on diagnosis and treatment of adult ADHD: The European Network Adult ADHD. BMC Psychiatry. 2010;10:67.

Laing A, Aristides M. Attention deficit hyperactivity disorder (ADHD) in adults: SF-6D utilities from SF-36 scores in a randomised trial. Poster presented at the 8th Annual European Congress of the International Society for Pharmacoeconomics and Outcomes Research; November 6-8, 2005. Florence, Italy.

Lloyd A, Hodgkins P, Sasane R, Akehurst R, Sonuga-Barke EJ, Fitzgerald P, et al. Estimation of utilities in attention-deficit hyperactivity disorder for economic evaluations. Patient. 2011;4(4):247-57.

Marfatia S, Shroff K, Munshi S, Tiwari A. Quality of life assessments in adults with attention deficit/hyperactivity disorder—a systematic review. Value Health. 2011;14(7):A327-8.

Mitsi G, Cragin L, Payne KA, Yu R, Goh J. Web-surveys: real world evidence gathering and minimizing uncertainty in economic models. Value Health. 2010;13(3):A121.

Moher D, Liberati A, Tetzlaff J, Altman DG; PRISMA Group. Preferred Reporting Items for Systematic Reviews and Meta-Analyses: the PRISMA statement. PLoS Med. 2009;6(7):e1000097.

National Institute for Health and Clinical Excellence (NICE). CG72: attention deficit hyperactivity disorder (ADHD). November 2008. Available at: <http://www.nice.org.uk/nicemedia/live/12061/42059/42059.pdf>. Accessed 10 January 2012.

National Institute for Health and Care Excellence (NICE). Guide to the methods of technology appraisal 2013. Process and methods guides. 4 April 2013. Available at: <http://www.nice.org.uk/media/D45/1E/GuideToMethodsTechnologyAppraisal2013.pdf>. Accessed 1 December 2013.

National Institute for Health and Care Excellence (NICE). Single technology appraisal: specification for manufacturer/sponsor submission of evidence. June 2012. Available at: <http://www.nice.org.uk/media/D54/6E/SpecificationForManufacturerSponsorSubmissionOfEvidenceJune2012.doc>. Accessed 1 December 2013.

National Collaborating Center for Mental Health. Attention deficit hyperactivity disorder: the NICE guideline on diagnosis and management of ADHD in children, young people, and adults. National Practice Guideline Number 72. The British Psychological Society, Leicester, UK, and The Royal College of Psychiatrists, London, UK; 2009. Available at: <http://www.nice.org.uk/nicemedia/live/12061/42060/42060.pdf>. Accessed 28 February 2014.

Péntek M, Gulacsi L, Hever NV, Papp S, Baji P, Brodszky V, et al. EQ-5D utilities and productivity of adults with attention-deficit/hyperactivity disorder: review of the literature and a cross-sectional survey in Hungary. Value Health. 2012;15(7):A340.

Polanczyk G, de Lima MS, Horta BL, Biederman J, Rohde LA. The worldwide prevalence of ADHD: a systematic review and meta-regression analysis. Am J Psychiatry, 2007;164:942-8.

Schlander M, Philipsen A, Schwarz O The cost effectiveness of clinically proven treatment strategies for attention-deficit/hyperactivity disorder (ADHD) in adult patients. Value Health. 2011;14(7):A403.

1. The *Methods Guide* (NICE, 2013) states that “Evidence on cost-effectiveness may be obtained from new analyses performed according to the NICE reference case; however, a systematic review of published, relevant evidence on the cost effectiveness of the technology should also be conducted.” [↑](#footnote-ref-1)
2. High-quality research presented as conference abstracts prior to 2012 would be expected to have been published in peer-reviewed journals by the time of this review and such publications are expected to be identified from the database searches. [↑](#footnote-ref-2)
3. The main goal of the review was to identify studies that would be suitable for use as data sources for the economic model evaluating the cost-effectiveness of lisdexamfetamine for the treatment of adults with ADHD. For a study to be applicable for use in the economic analysis, it has to report resource use, cost, or utility estimates by clinical response or disease severity. [↑](#footnote-ref-3)
4. This was limited in the database searches by “Adults (19+ years)” and “Adolescents (13-18 years)”. [↑](#footnote-ref-4)
5. The objective of the review was to report only the economic evaluations of LDX. However, all relevant economic evaluations, irrespective of the intervention evaluated, were included in level 1 screening because these studies may have included relevant utility and/or cost data. At level 2 screening, only those economic evaluations that were primary sources of these data or those that reported the cost-effectiveness of LDX were included. [↑](#footnote-ref-5)
6. Two economic evaluations in adults with ADHD were identified. One study evaluated the cost-effectiveness of cognitive behavioural therapy added to standard medication versus standard medication alone (National Collaborating Center for Mental Health, 2009). One study evaluated the cost-effectiveness of the following treatment options: 1) a structured disorder tailored psychotherapy (dialectical behavioural therapy) plus medication (methylphenidate); 2) dialectical behavioural therapy and placebo; 3) psychiatric counselling without specific behavioural interventions (clinical management) plus medication; or 4) clinical management and placebo (Schlander et al., 2011). These studies were screened at level 2 screen for any relevant utility or cost estimates. [↑](#footnote-ref-6)
7. There were no studies identified reporting health care resource use and costs by level of clinical response or disease severity. A number of studies reported costs for adults with ADHD versus those without ADHD. Such studies were not included in the review because these would not be appropriate for use in the current economic analysis. The results reported in these studies, however, could be used to validate the resource use estimates derived via a survey of the UK clinical experts. [↑](#footnote-ref-7)
8. For the reference case, the measurement of changes in health-related quality of life should be reported directly from patients, and the utility of these changes should be based on public preferences using a choice-based method (the time trade-off method). The EuroQol 5 Dimensions (EQ-5D) is the preferred measure of health-related quality of life in adults (NICE, 2013). [↑](#footnote-ref-8)
