# Supplementary material for: A cost-effectiveness analysis of lisdexamfetamine dimesylate in the treatment of adults with attention-deficit/hyperactivity disorder in the UK
Source: Eur J Health Econ. 2017 Jan 16;19(1):21–35. doi: 10.1007/s10198-016-0864-4 (PMC5773633; doi:10.1007/s10198-016-0864-4)
Supplement: Supplementary file 3 — Supplementary material 3 (DOC 856 kb) [file 10198_2016_864_MOESM3_ESM.doc]

**Supplementary Material: Market Research Report: Adult ADHD Resource Study:**

October 2014

Article title: A cost-effectiveness analysis of lisdexamfetamine dimesylate in the treatment of adults with attention-deficit/hyperactivity disorder in the United Kingdom

Journal: The European Journal of Health Economics

Authors: Evelina A Zimovetz, Alain Joseph, Rajeev Ayyagari, Josephine A Mauskopf

Corresponding author: Evelina Zimovetz, RTI Health Solutions; ezimovetz@rti.org

**Respondent profile & project history**

| **Specialty** | | |
| --- | --- | --- |
|  | **Total** | |
|  | **N** | **%** |
| Psychiatry | 60 | **100%** |
| Other | 0 | **0%** |
| **Base** | **60** | |

| **Location** | | |
| --- | --- | --- |
|  | Total | |
|  | **N** | **%** |
| England | 50 | **83.33%** |
| Scotland | 10 | **16.67%** |
| **Base** | **60** | |

**Fieldwork dates:** 23^rd^ September – 17^th^ October

**Sample:** 60 psychiatrists currently treating adult patients with ADHD.

**Methodology:** All respondents were invited via email and were recruited from the Medix database of healthcare professionals. Respondents were also screened prior to entering the survey to ensure that they are treating Adult ADHD (18yrs +) patients.

| **Are you a psychiatrist?** | **N** | **%** |
| --- | --- | --- |
| Yes | 60.00 | 100.00 |
| No | 0.00 | 0.00 |
| Total | 60.00 | |

| **Where in the UK are you currently practicing?** | **N** | **%** |
| --- | --- | --- |
| England | 50.00 | 83.33 |
| Scotland | 10.00 | 16.67 |
| Wales | 0.00 | 0.00 |
| Total | 60.00 | |
| **Are you treating adult patients (18 years of age and above) with ADHD?** | **N** | **%** |
| Yes | 60.00 | 100.00 |
| No | 0.00 | 0.00 |
| Total | 60.00 | |

| **How many adults with ADHD (new and existing) do you see per month?** | **N** | **%** |
| --- | --- | --- |
| 5.00 | 11.00 | 18.33 |
| 6.00 | 4.00 | 6.67 |
| 7.00 | 1.00 | 1.67 |
| 8.00 | 2.00 | 3.33 |
| 10.00 | 17.00 | 28.33 |
| 12.00 | 2.00 | 3.33 |
| 14.00 | 2.00 | 3.33 |
| 15.00 | 5.00 | 8.33 |
| 18.00 | 1.00 | 1.67 |
| 20.00 | 6.00 | 10.00 |
| 25.00 | 2.00 | 3.33 |
| 30.00 | 3.00 | 5.00 |
| 40.00 | 1.00 | 1.67 |
| 50.00 | 3.00 | 5.00 |
|  | **Mean** | **Std-deviation** |
| (Total) Scotland + England | 14.45 | 11.16 |
| **Base** | **60.00** | |

| **1) What percentage of your adult patients who are receiving treatment for ADHD are on pharmacotherapy?** | **N** | **%** |
| --- | --- | --- |
| 8.00 | 1.00 | 1.67 |
| 20.00 | 1.00 | 1.67 |
| 25.00 | 2.00 | 3.33 |
| 33.00 | 1.00 | 1.67 |
| 35.00 | 2.00 | 3.33 |
| 40.00 | 3.00 | 5.00 |
| 50.00 | 1.00 | 1.67 |
| 57.00 | 1.00 | 1.67 |
| 60.00 | 1.00 | 1.67 |
| 65.00 | 1.00 | 1.67 |
| 70.00 | 2.00 | 3.33 |
| 75.00 | 2.00 | 3.33 |
| 80.00 | 8.00 | 13.33 |
| 85.00 | 5.00 | 8.33 |
| 90.00 | 11.00 | 18.33 |
| 95.00 | 3.00 | 5.00 |
| 99.00 | 1.00 | 1.67 |
| 100.00 | 14.00 | 23.33 |
|  | **Mean** | **Std-deviation** |
| (Total) Scotland + England | 77.70 | 0.25 |
| **Base** | **60.00** | |

| **2) What percentage of your adult patients who are diagnosed with ADHD are not receiving any form of treatment at all?** | **N** | **%** |
| --- | --- | --- |
| 0% | 23.00 | 38.33 |
| 3% | 2.00 | 3.33 |
| 5% | 13.00 | 21.67 |
| 7% | 2.00 | 3.33 |
| 10% | 10.00 | 16.67 |
| 14% | 1.00 | 1.67 |
| 20% | 3.00 | 5.00 |
| 25% | 2.00 | 3.33 |
| 30% | 1.00 | 1.67 |
| 50% | 2.00 | 3.33 |
| 67% | 1.00 | 1.67 |
|  | **Mean** | **Std-deviation** |
| (Total) Scotland + England | 8.00 | 13.00 |
| **Base** | **60.00** | |

| **3) When you are initiating pharmacotherapy for an average adult patient with ADHD, in what percentage of patients do you only prescribe one drug (versus a combination therapy of two drugs)?** | **N** | **%** |
| --- | --- | --- |
| 20% | 1.00 | 1.67 |
| 25% | 1.00 | 1.67 |
| 35% | 1.00 | 1.67 |
| 40% | 2.00 | 3.33 |
| 50% | 1.00 | 1.67 |
| 58% | 2.00 | 3.33 |
| 60% | 3.00 | 5.00 |
| 65% | 2.00 | 3.33 |
| 70% | 1.00 | 1.67 |
| 75% | 6.00 | 10.00 |
| 80% | 7.00 | 11.67 |
| 89% | 1.00 | 1.67 |
| 90% | 7.00 | 11.67 |
| 95% | 2.00 | 3.33 |
| 97% | 1.00 | 1.67 |
| 99% | 3.00 | 5.00 |
| 100% | 19.00 | 31.67 |
|  | **Mean** | **Std-deviation** |
| (Total) Scotland + England | 82.21 | 21.00 |
| **Base** | **60.00** | |

| **3b) On average, what percentage of patients who are receiving pharmacotherapy for the first time (first line) are indicated on which drug and what is the average dose used for these patients? Methylphenidate** | **N** | **%** |
| --- | --- | --- |
| 0.00 | 2 | 3.33 |
| 5.00 | 1 | 1.67 |
| 10.00 | 3 | 5.00 |
| 15.00 | 1 | 1.67 |
| 20.00 | 1 | 1.67 |
| 25.00 | 3 | 5.00 |
| 33.00 | 1 | 1.67 |
| 35.00 | 2 | 3.33 |
| 40.00 | 2 | 3.33 |
| 45.00 | 3 | 5.00 |
| 50.00 | 5 | 8.33 |
| 55.00 | 2 | 3.33 |
| 60.00 | 7 | 11.67 |
| 70.00 | 8 | 13.33 |
| 75.00 | 3 | 5.00 |
| 80.00 | 5 | 8.33 |
| 85.00 | 2 | 3.33 |
| 90.00 | 2 | 3.33 |
| 95.00 | 1 | 1.67 |
| 100.00 | 6 | 10.00 |
|  | **Mean** | **Std-deviation** |
|  | 57.88 | 27.96 |
| **Base** | **60.00** | |

| **3b) On average, what percentage of patients who are receiving pharmacotherapy for the first time (first line) are indicated on which drug and what is the average dose used for these patients? Methylphenidate** | **N** | **%** |
| --- | --- | --- |
| 0.00 | 4 | 6.67 |
| 10.00 | 4 | 6.67 |
| 15.00 | 2 | 3.33 |
| 18.00 | 2 | 3.33 |
| 20.00 | 4 | 6.67 |
| 30.00 | 9 | 15.00 |
| 36.00 | 4 | 6.67 |
| 40.00 | 6 | 10.00 |
| 45.00 | 2 | 3.33 |
| 50.00 | 6 | 10.00 |
| 54.00 | 7 | 11.67 |
| 56.00 | 1 | 1.67 |
| 60.00 | 5 | 8.33 |
| 72.00 | 3 | 5.00 |
| 100.00 | 1 | 1.67 |
|  | **Mean** | **Std-deviation** |
|  | 38.00 | 20.79 |
| **Base** | **60.00** | |

| **3b) On average, what percentage of patients who are receiving pharmacotherapy for the first time (first line) are indicated on which drug and what is the average dose used for these patients? Atomoxetine** | **N** | **%** |
| --- | --- | --- |
| 0.00 | 8 | 13.33 |
| 1.00 | 1 | 1.67 |
| 5.00 | 1 | 1.67 |
| 10.00 | 7 | 11.67 |
| 12.00 | 2 | 3.33 |
| 15.00 | 2 | 3.33 |
| 20.00 | 11 | 18.33 |
| 25.00 | 7 | 11.67 |
| 30.00 | 3 | 5.00 |
| 34.00 | 1 | 1.67 |
| 35.00 | 1 | 1.67 |
| 40.00 | 4 | 6.67 |
| 45.00 | 4 | 6.67 |
| 50.00 | 2 | 3.33 |
| 70.00 | 1 | 1.67 |
| 85.00 | 1 | 1.67 |
| 90.00 | 3 | 5.00 |
| 100.00 | 1 | 1.67 |
|  | **Mean** | **Std-deviation** |
| (Total) Scotland + England | 27.48 | 24.68 |
| **Base** | **60.00** | |
| **3b) On average, what percentage of patients who are receiving pharmacotherapy for the first time (first line) are indicated on which drug and what is the average dose used for these patients? Atomoxetine** | **N** | **%** |
| 0.00 | 9.00 | 15.00 |
| 15.00 | 1.00 | 1.67 |
| 30.00 | 2.00 | 3.33 |
| 40.00 | 14.00 | 23.33 |
| 50.00 | 1.00 | 1.67 |
| 60.00 | 5.00 | 8.33 |
| 65.00 | 1.00 | 1.67 |
| 72.00 | 1.00 | 1.67 |
| 80.00 | 22.00 | 36.67 |
| 100.00 | 4.00 | 6.67 |
|  | **Mean** | **Std-deviation** |
| (Total) Scotland + England | 54.70 | 30.70 |
| **Base** | **60.00** | |

| **3b) On average, what percentage of patients who are receiving pharmacotherapy for the first time (first line) are indicated on which drug and what is the average dose used for these patients? Lisdexamphetamine** | **N** | **%** |
| --- | --- | --- |
| 0.00 | 38.00 | 63.33 |
| 3.00 | 4.00 | 6.67 |
| 5.00 | 1.00 | 1.67 |
| 10.00 | 9.00 | 15.00 |
| 15.00 | 1.00 | 1.67 |
| 20.00 | 3.00 | 5.00 |
| 25.00 | 2.00 | 3.33 |
| 30.00 | 2.00 | 3.33 |
|  | **Mean** | **Std-deviation** |
| (Total) Scotland + England | 4.87 | 8.25 |
| **Base** | **60.00** | |

| **3b) On average, what percentage of patients who are receiving pharmacotherapy for the first time (first line) are indicated on which drug and what is the average dose used for these patients? Lisdexamphetamine** | **N** | **%** |
| --- | --- | --- |
| 0.00 | 38.00 | 63.33 |
| 30.00 | 6.00 | 10.00 |
| 40.00 | 3.00 | 5.00 |
| 50.00 | 6.00 | 10.00 |
| 51.00 | 1.00 | 1.67 |
| 60.00 | 2.00 | 3.33 |
| 70.00 | 4.00 | 6.67 |
|  | **Mean** | **Std-deviation** |
| (Total) Scotland + England | 17.52 | 24.77 |
| **Base** | **60.00** | |
| **3b) On average, what percentage of patients who are receiving pharmacotherapy for the first time (first line) are indicated on which drug and what is the average dose used for these patients? Dexamphetamine** | **N** | **%** |
| 0.00 | 27.00 | 45.00 |
| 2.00 | 2.00 | 3.33 |
| 5.00 | 6.00 | 10.00 |
| 9.00 | 1.00 | 1.67 |
| 10.00 | 13.00 | 21.67 |
| 15.00 | 4.00 | 6.67 |
| 20.00 | 2.00 | 3.33 |
| 25.00 | 2.00 | 3.33 |
| 30.00 | 1.00 | 1.67 |
| 33.00 | 1.00 | 1.67 |
| 35.00 | 1.00 | 1.67 |
|  | **Mean** | **Std-deviation** |
| (Total) Scotland + England | 24.77 | 8.97 |
| **Base** | **60.00** | |

| **3b) On average, what percentage of patients who are receiving pharmacotherapy for the first time (first line) are indicated on which drug and what is the average dose used for these patients? Dexamphetamine** | **N** | **%** |
| --- | --- | --- |
| 0.00 | 27.00 | 45.00 |
| 5.00 | 2.00 | 3.33 |
| 10.00 | 5.00 | 8.33 |
| 15.00 | 5.00 | 8.33 |
| 20.00 | 2.00 | 3.33 |
| 30.00 | 5.00 | 8.33 |
| 40.00 | 13.00 | 21.67 |
| 50.00 | 1.00 | 1.67 |
|  | **Mean** | **Std-deviation** |
| (Total) Scotland + England | 14.92 | 16.94 |
| **Base** | **60.00** | |

| **3b) On average, what percentage of patients who are receiving pharmacotherapy for the first time (first line) are indicated on which drug and what is the average dose used for these patients? Other** | **N** | **%** |
| --- | --- | --- |
| 0.00 | 52.00 | 86.67 |
| 5.00 | 4.00 | 6.67 |
| 15.00 | 2.00 | 3.33 |
| 20.00 | 1.00 | 1.67 |
| 95.00 | 1.00 | 1.67 |
|  | **Mean** | **Std-deviation** |
| (Total) Scotland + England | 2.75 | 12.70 |
| **Base** | **60.00** | |

| **3b) On average, what percentage of patients who are receiving pharmacotherapy for the first time (first line) are indicated on which drug and what is the average dose used for these patients? Other** | **N** | **%** |
| --- | --- | --- |
| 0.00 | 52.00 | 86.67 |
| 15.00 | 1.00 | 1.67 |
| 40.00 | 3.00 | 5.00 |
| 50.00 | 3.00 | 3.33 |
| 100.00 | 1.00 | 1.67 |
|  | **Mean** | **Std-deviation** |
| (Total) Scotland + England | 8.08 | 25.58 |
| **Base** | **60.00** | |

| **3c) On average, what percentage of patients who are 2nd line patients are you prescribing which drug and what is the average dose used for these patients? Methylphenidate** | **N** | **%** |
| --- | --- | --- |
| 0.00 | 4.00 | 6.67 |
| 5.00 | 1.00 | 1.67 |
| 10.00 | 4.00 | 6.67 |
| 15.00 | 4.00 | 6.67 |
| 20.00 | 9.00 | 15.00 |
| 25.00 | 3.00 | 5.00 |
| 30.00 | 6.00 | 10.00 |
| 33.00 | 3.00 | 5.00 |
| 35.00 | 1.00 | 1.67 |
| 40.00 | 10.00 | 16.67 |
| 45.00 | 1.00 | 1.67 |
| 50.00 | 4.00 | 6.67 |
| 60.00 | 3.00 | 5.00 |
| 80.00 | 2.00 | 3.33 |
| 90.00 | 1.00 | 1.67 |
| 100.00 | 4.00 | 6.67 |
|  | **Mean** | **Std-deviation** |
| (Total) Scotland + England | 35.82 | 25.86 |
| **Base** | **60.00** | |

| **3c) On average, what percentage of patients who are 2nd line patients are you prescribing which drug and what is the average dose for these patients? Methylphenidate** | **N** | **%** |
| --- | --- | --- |
| 0.00 | 5.00 | 8.33 |
| 10.00 | 4.00 | 6.67 |
| 20.00 | 4.00 | 6.67 |
| 30.00 | 10.00 | 16.67 |
| 36.00 | 1.00 | 1.67 |
| 40.00 | 4.00 | 6.67 |
| 45.00 | 1.00 | 1.67 |
| 50.00 | 2.00 | 3.33 |
| 54.00 | 11.00 | 18.33 |
| 56.00 | 1.00 | 1.67 |
| 60.00 | 10.00 | 16.67 |
| 72.00 | 3.00 | 5.00 |
| 80.00 | 1.00 | 1.67 |
| 90.00 | 1.00 | 1.67 |
| 100.00 | 2.00 | 3.33 |
|  | **Mean** | **Std-deviation** |
| (Total) Scotland + England | 43.28 | 24.21 |
| **Base** | **60.00** | |

| **3c) On average, what percentage of patients who are 2nd line patients are you prescribing which drug and what is the average dose used for these patients? Atomoxetine** | **N** | **%** |
| --- | --- | --- |
| 0.00 | 5.00 | 8.33 |
| 5.00 | 1.00 | 1.67 |
| 10.00 | 1.00 | 1.67 |
| 15.00 | 3.00 | 5.00 |
| 20.00 | 4.00 | 6.67 |
| 25.00 | 5.00 | 8.33 |
| 30.00 | 6.00 | 10.00 |
| 35.00 | 1.00 | 1.67 |
| 37.00 | 1.00 | 1.67 |
| 40.00 | 7.00 | 11.67 |
| 45.00 | 1.00 | 1.67 |
| 50.00 | 5.00 | 8.33 |
| 53.00 | 2.00 | 3.33 |
| 60.00 | 6.00 | 10.00 |
| 66.00 | 1.00 | 1.67 |
| 67.00 | 1.00 | 1.67 |
| 70.00 | 4.00 | 6.67 |
| 80.00 | 2.00 | 3.33 |
| 90.00 | 2.00 | 3.33 |
| 95.00 | 1.00 | 1.67 |
| 100.00 | 1.00 | 1.67 |
|  | **Mean** | **Std-deviation** |
| (Total) Scotland + England | 41.77 | 25.47 |
| **Base** | **60.00** | |

| **3c) On average, what percentage of patients who are 2nd line patients are you prescribing which drug and what is the average dose for these patients? Atomoxetine** | **N** | **%** |
| --- | --- | --- |
| 0.00 | 7.00 | 11.67 |
| 10.00 | 1.00 | 1.67 |
| 15.00 | 1.00 | 1.67 |
| 20.00 | 1.00 | 1.67 |
| 30.00 | 1.00 | 1.67 |
| 40.00 | 13.00 | 21.67 |
| 60.00 | 3.00 | 5.00 |
| 65.00 | 1.00 | 1.67 |
| 70.00 | 1.00 | 1.67 |
| 80.00 | 24.00 | 40.00 |
| 90.00 | 1.00 | 1.67 |
| 100.00 | 5.00 | 8.33 |
|  | **Mean** | **Std-deviation** |
| (Total) Scotland + England | 59.00 | 31.78 |
| **Base** | **60.00** | |

| **3c) On average, what percentage of patients who are 2nd line patients are you prescribing which drug and what is the average dose used for these patients? Lisdexamphetamine** | **N** | **%** |
| --- | --- | --- |
| 0.00 | 39 | 65.00 |
| 2.00 | 1.00 | 1.67 |
| 10.00 | 7.00 | 11.67 |
| 15.00 | 3.00 | 5.00 |
| 20.00 | 3.00 | 5.00 |
| 25.00 | 1.00 | 1.67 |
| 30.00 | 6.00 | 10.00 |
|  | **Mean** | **Std-deviation** |
| (Total) Scotland + England | 6.37 | 10.23 |
| **Base** | **60.00** | |

| **3c) On average, what percentage of patients who are 2nd line patients are you prescribing which drug and what is the average dose for these patients? Lisdexamphetamine** | **N** | **%** |
| --- | --- | --- |
| 0.00 | 39.00 | 65.00 |
| 5.00 | 1.00 | 1.67 |
| 20.00 | 1.00 | 1.67 |
| 30.00 | 4.00 | 6.67 |
| 40.00 | 2.00 | 3.33 |
| 50.00 | 7.00 | 11.67 |
| 60.00 | 1.00 | 1.67 |
| 70.00 | 5.00 | 8.33 |
|  | **Mean** | **Std-deviation** |
| (Total) Scotland + England | 16.42 | 24.93 |
| **Base** | **60.00** | |

| **3c) On average, what percentage of patients who are 2nd line patients are you prescribing which drug and what is the average dose used for these patients? Dexamphetamine** | **N** | **%** |
| --- | --- | --- |
| 0.00 | 31.00 | 51.67 |
| 1.00 | 1.00 | 1.67 |
| 5.00 | 2.00 | 3.33 |
| 10.00 | 6.00 | 10.00 |
| 14.00 | 1.00 | 1.67 |
| 15.00 | 3.00 | 5.00 |
| 20.00 | 8.00 | 13.33 |
| 25.00 | 1.00 | 1.67 |
| 35.00 | 1.00 | 1.67 |
| 50.00 | 3.00 | 5.00 |
| 75.00 | 1.00 | 1.67 |
| 80.00 | 1.00 | 1.67 |
| 85.00 | 1.00 | 1.67 |
|  | **Mean** | **Std-deviation** |
| (Total) Scotland + England | 12.33 | 20.25 |
| **Base** | **60.00** | |

| **3c) On average, what percentage of patients who are 2nd line patients are you prescribing which drug and what is the average dose for these patients? Dexamphetamine** | **N** | **%** |
| --- | --- | --- |
| 0.00 | 31.00 | 51.67 |
| 10.00 | 5.00 | 8.33 |
| 15.00 | 3.00 | 5.00 |
| 20.00 | 3.00 | 5.00 |
| 25.00 | 1.00 | 1.67 |
| 30.00 | 7.00 | 11.67 |
| 40.00 | 3.00 | 5.00 |
| 45.00 | 1.00 | 1.67 |
| 50.00 | 3.00 | 5.00 |
| 60.00 | 3.00 | 5.00 |
|  | **Mean** | **Std-deviation** |
| (Total) Scotland + England | 14.75 | 19.03 |
| **Base** | **60.00** | |

| **3c) On average, what percentage of patients who are 2nd line patients are you prescribing which drug and what is the average dose used for these patients? Other** | **N** | **%** |
| --- | --- | --- |
| 0.00 | 47.00 | 78.33 |
| 10.00 | 4.00 | 6.67 |
| 14.00 | 2.00 | 3.33 |
| 20.00 | 4.00 | 6.67 |
| 25.00 | 3.00 | 5.00 |
|  | **Mean** | **Std-deviation** |
| (Total) Scotland + England | 3.72 | 7.63 |
| **Base** | **60.00** | |

| **3c) On average, what percentage of patients who are 2nd line patients are you prescribing which drug and what is the average dose for these patients? Other** | **N** | **%** |
| --- | --- | --- |
| 0.00 | 47.00 | 78.33 |
| 8.00 | 2.00 | 3.33 |
| 10.00 | 2.00 | 3.33 |
| 15.00 | 2.00 | 3.33 |
| 18.00 | 1.00 | 1.67 |
| 40.00 | 1.00 | 1.67 |
| 50.00 | 3.00 | 5.00 |
|  | **Mean** | **Std-deviation** |
| (Total) Scotland + England | 5.23 | 12.59 |
| **Base** | **60.00** | |

| **4) In adult patients with ADHD in whom you are initiating pharmacotherapy with a combination therapy, what combinations are you using in which strength? Combination A** | **mg** | **mg** |
| --- | --- | --- |
| Methylphenidate & Atomoxetine 100% | 72.00 | 40.00 |
| Methylphenidate & Atomoxetine 100% | 60.00 | 80.00 |
| Methylphenidate & Atomoxetine 100% | 60.00 | 40.00 |
| Methylphenidate & Atomoxetine 100% | 40.00 | 60.00 |
| Methylphenidate & Atomoxetine 100% | 50.00 | 80.00 |
| Methylphenidate & Atomoxetine 100% | 40.00 | 50.00 |
| Methylphenidate & Atomoxetine 100% | 72.00 | 80.00 |
| Methylphenidate & Atomoxetine 100% | 24.00 | 24.00 |
| Methylphenidate & Atomoxetine 100% | 80.00 | 15.00 |
| Methylphenidate & Atomoxetine 100% | 54.00 | 60.00 |
| Methylphenidate & Atomoxetine 98% | 54.00 | 20.00 |
| Methylphenidate & Atomoxetine 80% | 10.00 | 60.00 |
| Methylphenidate & Atomoxetine 75% | 72.00 | 60.00 |
| Methylphenidate & Atomoxetine 75% | 72.00 | 60.00 |
| Methylphenidate & Atomoxetine 75% | 40.00 | 80.00 |
| Methylphenidate & Atomoxetine 60% | 40.00 | 30.00 |
| Methylphenidate & Atomoxetine 60% | 30.00 | 80.00 |
| Methylphenidate & Atomoxetine 60% | 15.00 | 60.00 |
| Methylphenidate & Atomoxetine 50% | 30.00 | 40.00 |
| Methylphenidate & Atomoxetine 50% | 30.00 | 60.00 |
| Methylphenidate & Atomoxetine 50% | 36.00 | 60.00 |
| Methylphenidate & Atomoxetine 50% | 36.00 | 40.00 |
| Methylphenidate & Atomoxetine 50% | 36.00 | 80.00 |
| Methylphenidate & Atomoxetine 50% | 30.00 | 40.00 |
| Methylphenidate & Atomoxetine 50% | 2.00 | 1.00 |
| Methylphenidate & Atomoxetine 40% | 40.00 | 75.00 |
| Methylphenidate & Atomoxetine 40% | 40.00 | 75.00 |
| Methylphenidate & Atomoxetine 40% | 10.00 | 20.00 |
| Methylphenidate & Atomoxetine 33% | 72.00 | 30.00 |
| Methylphenidate & Atomoxetine 33% | 72.00 | 30.00 |
| Methylphenidate & Atomoxetine 30% | 1.00 | 1.00 |
| Methylphenidate & Atomoxetine 20% | 1.00 | 1.00 |
| Methylphenidate & Atomoxetine 10% | 9.00 | 1.00 |
| Methylphenidate & Other 100% | 10.00 | 100.00 |
| Methylphenidate & Other 100% | 30.00 | 20.00 |
| Methylphenidate & Other 100% | 30.00 | 1.00 |
| Methylphenidate & Other 100% | 30.00 | 1.00 |
| Methylphenidate & Other 100% | 30.00 | 1.00 |
| Methylphenidate & Other 100% | 56.00 | 5.00 |
| Methylphenidate & Other 80% | 20.00 | 20.00 |
| Methylphenidate & Other 80% | 20.00 | 20.00 |
| Methylphenidate & Other 50% | 80.00 | 1.00 |
| Methylphenidate & Other 45% | 60.00 | 2.00 |
| Methylphenidate & Other 25% | 85.00 | 30.00 |
| Methylphenidate & Other 5% | 6.00 | 60.00 |
| Methylphenidate & Other 5% | 6.00 | 60.00 |
| Methylphenidate & Dexamphetamine 100% | 86.00 | 5.00 |
| Methylphenidate & Dexamphetamine 100% | 36.00 | 15.00 |
| Methylphenidate & Dexamphetamine 100% | 20.00 | 10.00 |
| Atomoxetine & Methylphenidate 100% | 60.00 | 10.00 |
| Atomoxetine & Methylphenidate 60% | 60.00 | 27.00 |
| Atomoxetine & Other 100% | 50.00 | 10.00 |
| Atomoxetine & Other 50% | 80.00 | 50.00 |
| Atomoxetine & Other 50% | 80.00 | 50.00 |
| Atomoxetine & Other 35% | 70.00 | 50.00 |
| Atomoxetine & Other 35% | 70.00 | 50.00 |
| Methylphenidate and Lisdexamphetamine 35% | 40.00 | 50.00 |
| Methylphenidate and Lisdexamphetamine 35% | 40.00 | 50.00 |
| Other and other 100% | 1.00 | 1.00 |
| N/A | 0.00 | 0.00 |
| **Base** | **60.00** | |

| **4) In adult patients with ADHD in whom you are initiating pharmacotherapy with a combination therapy, what combinations are you using in which strength? Combination B** | **mg** | **mg** |
| --- | --- | --- |
| Methylphenidate & Other 60% | 55.00 | 15.00 |
| Methylphenidate & Other 60% | 55.00 | 15.00 |
| Methylphenidate & Other 50% | 36.00 | 50.00 |
| Methylphenidate & Other 50% | 36.00 | 50.00 |
| Methylphenidate & Other 40% | 54.00 | 20.00 |
| Methylphenidate & Other 35% | 40.00 | 60.00 |
| Methylphenidate & Other 35% | 40.00 | 60.00 |
| Methylphenidate & Other 30% | 54.00 | 50.00 |
| Methylphenidate & Other 25% | 30.00 | 100.00 |
| Methylphenidate & Other 25% | 30.00 | 100.00 |
| Methylphenidate & Other 0% | 30.00 | 1.00 |
| Methylphenidate & Other 0% | 30.00 | 1.00 |
| Methylphenidate & Dexamphetamineine 45% | 12.00 | 8.00 |
| Methylphenidate & Dexamphetamineine 35% | 40.00 | 30.00 |
| Methylphenidate & Dexamphetamineine 35% | 40.00 | 30.00 |
| Methylphenidate & Dexamphetamine 30% | 20.00 | 10.00 |
| Methylphenidate & Dexamphetamine 20% | 38.00 | 20.00 |
| Methylphenidate & Dexamphetamine 2% | 40.00 | 10.00 |
| Atomoxetine & Methylphenidate 50% | 60.00 | 30.00 |
| Atomoxetine & Methylphenidate 40% | 1.00 | 1.00 |
| Atomoxetine & Methylphenidate 30% | 1.00 | 1.00 |
| Atomoxetine & Methylphenidate 10% | 80.00 | 40.00 |
| Atomoxetine & Methylphenidate 10% | 80.00 | 40.00 |
| Atomoxetine & Other 70% | 72.00 | 30.00 |
| Atomoxetine & Other 45% | 80.00 | 2.00 |
| Atomoxetine & Other 15% | 100.00 | 20.00 |
| Atomoxetine & Other 15% | 100.00 | 20.00 |
| Atomoxetine & Lisdaxamphetamine 20% | 60.00 | 30.00 |
| Atomoxetine & Lisdaxamphetamine 20% | 60.00 | 30.00 |
| Atomoxetine & Lisdaxamphetamine 1% | 20.00 | 25.00 |
| Atomoxetine & Dexamphetamine 20% | 80.00 | 20.00 |
| Atomoxetine & Dexamphetamine 25% | 40.00 | 20.00 |
| Lisdexamphetamine & Other 25% | 65.00 | 1.00 |
| Dexamphetamine & Atomoxetine 20% | 15.00 | 60.00 |
| Lisdexamphetamine & Atomoxetine 25% | 50.00 | 80.00 |
| Dexamphetamine & Methylphenidate 30% | 5.00 | 2.00 |
| Methylphenidate & Lisdexamphetamine 34% | 72.00 | 30.00 |
| Methylphenidate & Lisdexamphetamine 34% | 72.00 | 30.00 |
| Methylphenidate & Atomoxetine 0% | 1.00 | 1.00 |
| Other & Methylphenidate 19% | 36.00 | 30.00 |
| N/A 20 | 0.00 | 0.00 |
| **Base** | **60.00** | |

| **4) In adult patients with ADHD in whom you are initiating pharmacotherapy with a combination therapy, what combinations are you using in which strength? Combination C** | **mg** | **mg** |
| --- | --- | --- |
| Methylphenidate & Dexamphetamineine 33% | 72.00 | 30.00 |
| Methylphenidate & Dexamphetamineine 33% | 72.00 | 30.00 |
| Methylphenidate & Dexamphetamineine 20% | 20.00 | 30.00 |
| Methylphenidate & Dexamphetamineine 15% | 40.00 | 40.00 |
| Methylphenidate & Lisdexamphetamine 40% | 1.00 | 1.00 |
| Methylphenidate & Lisdexamphetamine 5% | 54.00 | 30.00 |
| Methylphenidate & Lisdexamphetamine 5% | 54.00 | 30.00 |
| Atomoxetine & Other 25% | 80.00 | 1.00 |
| Atomoxetine & Other 25% | 40.00 | 100.00 |
| Atomoxetine & Other 25% | 40.00 | 100.00 |
| Atomoxetine & Other 5% | 72.00 | 10.00 |
| Dexamphetamine & Atomoxetine 20% | 5.00 | 3.00 |
| Dexamphetamine & Atomoxetine 20% | 30.00 | 60.00 |
| Dexamphetamine & Other 85% | 60.00 | 75.00 |
| Dexamphetamine & Other 85% | 60.00 | 75.00 |
| Dexamphetamine & Other 30% | 40.00 | 50.00 |
| Dexamphetamine & Other 30% | 40.00 | 50.00 |
| Dexamphetamine & Other 1% | 15.00 | 12.00 |
| Dexamphetamine & Other 0% | 1.00 | 1.00 |
| Methylphenidate & Other 20% | 30.00 | 50.00 |
| Methylphenidate & Other 23% | 40.00 | 75.00 |
| Methylphenidate & Other 0% | 30.00 | 1.00 |
| Methylphenidate & Other 0% | 30.00 | 1.00 |
| Methylphenidate & Other 0% | 70.00 | 1.00 |
| Methylphenidate & Atomoxetine 5% | 20.00 | 100.00 |
| Methylphenidate & Atomoxetine 5% | 20.00 | 100.00 |
| Methylphenidate & Atomoxetine 0% | 1.00 | 1.00 |
| Lisdexamphetamine & Atomoxetine 30% | 70.00 | 40.00 |
| Lisdexamphetamine & Atomoxetine 30% | 70.00 | 40.00 |
| Lisdexamphetamine & Atomoxetine 10% | 30.00 | 60.00 |
| Lisdexamphetamine & other 10% | 30.00 | 2.00 |
| Dexamphetamine & Lisdexamphetamine 40% | 1.00 | 1.00 |
| Atomoxetine & Dexamphetamine 30% | 20.00 | 10.00 |
| Atomoxetine & Dexamphetamine 1% | 20.00 | 20.00 |
| Atomoxetine & Methylphenidate 45% | 6.00 | 3.00 |
| Lisdexamphetamine & Methylphenidate 25% | 50.00 | 18.00 |
| Lisdexamphetamine & Dexamphetamine 20% | 20.00 | 20.00 |
| Atomoxetine & other 5% | 72.00 | 10.00 |
| Other & Other 0% | 1.00 | 1.00 |
| Other & Other 0% | 1.00 | 1.00 |
| N/A 20 | 0.00 | 0.00 |
| **Base** | **60.00** | |

| **4a) When you are initiating pharmacotherapy with Methylphenidate please provide the initial dose.** | | |
| --- | --- | --- |
|  | **N** | **%** |
| N/A | 3.00 | 5.00 |
| 2.00 | 2.00 | 3.33 |
| 5.00 | 5.00 | 8.33 |
| 10.00 | 22.00 | 36.67 |
| 15.00 | 4.00 | 6.67 |
| 18.00 | 14.00 | 23.33 |
| 20.00 | 4.00 | 6.67 |
| 30.00 | 5.00 | 8.33 |
| 36.00 | 1.00 | 1.67 |
|  | **Mean** | **Std-deviation** |
| (Total) Scotland + England | 13.78 | 7.99 |
| **Base** | **60.00** | |

| **4a) When you are initiating pharmacotherapy with Methylphenidate please provide the doses used during a titration phase.** | | |
| --- | --- | --- |
|  | **N** | **%** |
| 0.00 | 3.00 | 5.00 |
| 5.00 | 3.00 | 5.00 |
| 10.00 | 10.00 | 16.67 |
| 15.00 | 4.00 | 6.67 |
| 18.00 | 1.00 | 1.67 |
| 20.00 | 15.00 | 25.00 |
| 25.00 | 1.00 | 1.67 |
| 27.00 | 4.00 | 6.67 |
| 30.00 | 3.00 | 5.00 |
| 36.00 | 9.00 | 15.00 |
| 40.00 | 4.00 | 6.67 |
| 54.00 | 1.00 | 1.67 |
| 60.00 | 2.00 | 3.33 |
|  | **Mean** | **Std-deviation** |
| (Total) Scotland + England | 22.90 | 13.81 |
| **Base** | **60.00** | |

| **4a) When you are initiating pharmacotherapy with Methylphenidate please provide the dose used during the titration phase.** | | |
| --- | --- | --- |
|  | **N** | **%** |
| 0.00 | 4.00 | 6.67 |
| 5.00 | 1.00 | 1.67 |
| 10.00 | 7.00 | 11.67 |
| 15.00 | 2.00 | 3.33 |
| 18.00 | 1.00 | 1.67 |
| 20.00 | 5.00 | 8.33 |
| 25.00 | 1.00 | 1.67 |
| 30.00 | 14.00 | 23.33 |
| 36.00 | 4.00 | 6.67 |
| 40.00 | 1.00 | 1.67 |
| 45.00 | 3.00 | 5.00 |
| 50.00 | 3.00 | 5.00 |
| 54.00 | 9.00 | 15.00 |
| 60.00 | 2.00 | 3.33 |
| 72.00 | 1.00 | 1.67 |
| 80.00 | 1.00 | 1.67 |
| 86.00 | 1.00 | 1.67 |
|  | **Mean** | **Std-deviation** |
| (Total) Scotland + England | 33.02 | 20.21 |
| **Base** | **60.00** | |

| **4a) When you are initiating pharmacotherapy with Methylphenidate please provide the dose used during the titration phase.** | | |
| --- | --- | --- |
|  | **N** | **%** |
| 0.00 | 13.00 | 21.67 |
| 10.00 | 6.00 | 10.00 |
| 15.00 | 2.00 | 3.33 |
| 18.00 | 1.00 | 1.67 |
| 20.00 | 1.00 | 1.67 |
| 25.00 | 3.00 | 5.00 |
| 30.00 | 2.00 | 3.33 |
| 40.00 | 12.00 | 20.00 |
| 45.00 | 3.00 | 5.00 |
| 54.00 | 2.00 | 3.33 |
| 60.00 | 4.00 | 6.67 |
| 72.00 | 7.00 | 11.67 |
| 75.00 | 1.00 | 1.67 |
| 80.00 | 2.00 | 3.33 |
| 120.00 | 1.00 | 1.67 |
|  | **Mean** | **Std-deviation** |
| (Total) Scotland + England | 34.75 | 28.41 |
| **Base** | **60.00** | |

| **4a) When you are initiating pharmacotherapy with Methylphenidate please provide the maintenance dose.** | | |
| --- | --- | --- |
|  | **N** | **%** |
| 0.00 | 3.00 | 5.00 |
| 10.00 | 1.00 | 1.67 |
| 15.00 | 2.00 | 3.33 |
| 20.00 | 4.00 | 6.67 |
| 25.00 | 1.00 | 1.67 |
| 30.00 | 5.00 | 8.33 |
| 36.00 | 3.00 | 5.00 |
| 40.00 | 8.00 | 13.33 |
| 45.00 | 4.00 | 6.67 |
| 50.00 | 2.00 | 3.33 |
| 54.00 | 7.00 | 11.67 |
| 56.00 | 1.00 | 1.67 |
| 60.00 | 12.00 | 20.00 |
| 72.00 | 1.00 | 1.67 |
| 75.00 | 1.00 | 1.67 |
| 80.00 | 1.00 | 1.67 |
| 85.00 | 1.00 | 1.67 |
| 86.00 | 1.00 | 1.67 |
| 100.00 | 1.00 | 1.67 |
| 108.00 | 1.00 | 1.67 |
|  | **Mean** | **Std-deviation** |
| (Total) Scotland + England | 46.05 | 22.58 |
| **Base** | **60.00** | |

| **4b) When you are initiating pharmacotherapy with Atomoxetine please provide the initial dose.** | | |
| --- | --- | --- |
|  | **N** | **%** |
| 0.00 | 11.00 | 18.33 |
| 5.00 | 1.00 | 1.67 |
| 10.00 | 12.00 | 20.00 |
| 15.00 | 1.00 | 1.67 |
| 18.00 | 3.00 | 5.00 |
| 20.00 | 11.00 | 18.33 |
| 30.00 | 3.00 | 5.00 |
| 40.00 | 18.00 | 30.00 |
|  | **Mean** | **Std-deviation** |
| (Total) Scotland + England | 20.40 | 15.04 |
| **Base** | **60.00** | |

| **4b) When you are initiating pharmacotherapy with Atomoxetine please provide the doses used during a titration phase.** | | |
| --- | --- | --- |
|  | **N** | **%** |
| NA | 11.00 | 18.33 |
| 2.00 | 1.00 | 1.67 |
| 10.00 | 1.00 | 1.67 |
| 15.00 | 1.00 | 1.67 |
| 18.00 | 2.00 | 3.33 |
| 20.00 | 15.00 | 25.00 |
| 25.00 | 1.00 | 1.67 |
| 36.00 | 1.00 | 1.67 |
| 40.00 | 9.00 | 15.00 |
| 50.00 | 5.00 | 8.33 |
| 60.00 | 8.00 | 13.33 |
| 80.00 | 4.00 | 6.67 |
| 84.00 | 1.00 | 1.67 |
|  | **Mean** | **Std-deviation** |
| (Total) Scotland + England | 31.97 | 24.68 |
| **Base** | **60.00** | |

| **4b) When you are initiating pharmacotherapy with Atomoxetine please provide the doses used during a titration phase.** | | |
| --- | --- | --- |
|  | **N** | **%** |
| NA | 15.00 | 25.00 |
| 15.00 | 2.00 | 3.33 |
| 20.00 | 4.00 | 6.67 |
| 25.00 | 1.00 | 1.67 |
| 30.00 | 5.00 | 8.33 |
| 40.00 | 8.00 | 13.33 |
| 54.00 | 1.00 | 1.67 |
| 60.00 | 11.00 | 18.33 |
| 80.00 | 10.00 | 16.67 |
| 100.00 | 1.00 | 1.67 |
| 120.00 | 2.00 | 3.33 |
|  | **Mean** | **Std-deviation** |
| (Total) Scotland + England | 40.98 | 33.07 |
| **Base** | **60.00** | |

| **4b) When you are initiating pharmacotherapy with Atomoxetine please provide the doses used during a titration phase.** | | |
| --- | --- | --- |
|  | **N** | **%** |
| NA | 20.00 | 33.33 |
| 10.00 | 2.00 | 3.33 |
| 15.00 | 1.00 | 1.67 |
| 20.00 | 3.00 | 5.00 |
| 40.00 | 7.00 | 11.67 |
| 60.00 | 4.00 | 6.67 |
| 70.00 | 3.00 | 5.00 |
| 72.00 | 1.00 | 1.67 |
| 80.00 | 11.00 | 18.33 |
| 100.00 | 6.00 | 10.00 |
| 120.00 | 2.00 | 3.33 |
|  | **Mean** | **Std-deviation** |
| (Total) Scotland + England | 43.62 | 39.52 |
| **Base** | **60.00** | |

| **4b) When you are initiating pharmacotherapy with Atomoxetine please provide the maintenance dose.** | | |
| --- | --- | --- |
|  | **N** | **%** |
| NA | 12.00 | 20.00 |
| 15.00 | 1.00 | 1.67 |
| 30.00 | 2.00 | 3.33 |
| 40.00 | 10.00 | 16.67 |
| 60.00 | 5.00 | 8.33 |
| 72.00 | 1.00 | 1.67 |
| 80.00 | 23.00 | 38.33 |
| 84.00 | 1.00 | 1.67 |
| 100.00 | 5.00 | 8.33 |
|  | **Mean** | **Std-deviation** |
| (Total) Scotland + England | 54.52 | 33.73 |
| **Base** | **60.00** | |

| **4c) When you are initiating pharmacotherapy with Lisdexamphetamine please provide the initial dose.** | | |
| --- | --- | --- |
|  | **N** | **%** |
| NA | 48.00 | 80.00 |
| 15.00 | 1.00 | 1.67 |
| 30.00 | 11.00 | 18.33 |
|  | **Mean** | **Std-deviation** |
| (Total) Scotland + England | 5.75 | 11.75 |
| **Base** | **60.00** | |

| **4c) When you are initiating pharmacotherapy with Lisdexamphetamine please provide the doses used during a titration phase.** | | |
| --- | --- | --- |
|  | **N** | **%** |
| NA | 48.00 | 80.00 |
| 10.00 | 1.00 | 1.67 |
| 30.00 | 1.00 | 1.67 |
| 40.00 | 5.00 | 8.33 |
| 50.00 | 5.00 | 8.33 |
|  | **Mean** | **Std-deviation** |
| (Total) Scotland + England | 8.17 | 17.22 |
| **Base** | **60.00** | |

| **4c) When you are initiating pharmacotherapy with Lisdexamphetamine please provide the doses used during a titration phase.** | | |
| --- | --- | --- |
|  | **N** | **%** |
| NA | 49.00 | 81.67 |
| 10.00 | 1.00 | 1.67 |
| 30.00 | 1.00 | 1.67 |
| 50.00 | 5.00 | 8.33 |
| 70.00 | 4.00 | 6.67 |
|  | **Mean** | **Std-deviation** |
| (Total) Scotland + England | 9.50 | 21.66 |
| **Base** | **60.00** | |

| **4c) When you are initiating pharmacotherapy with Lisdexamphetamine please provide the doses used during a titration phase.** | | |
| --- | --- | --- |
|  | **N** | **%** |
| NA | 51.00 | 85.00 |
| 10.00 | 1.00 | 1.67 |
| 50.00 | 1.00 | 1.67 |
| 60.00 | 4.00 | 6.67 |
| 70.00 | 3.00 | 5.00 |
|  | **Mean** | **Std-deviation** |
| (Total) Scotland + England | 8.50 | 21.54 |
| **Base** | **60.00** | |

| **4c) When you are initiating pharmacotherapy with Lisdexamphetamine please provide the maintenance dose.** | | |
| --- | --- | --- |
|  | **N** | **%** |
| NA | 48.00 | 80.00 |
| 30.00 | 2.00 | 3.33 |
| 40.00 | 1.00 | 1.67 |
| 50.00 | 3.00 | 5.00 |
| 60.00 | 3.00 | 5.00 |
| 70.00 | 3.00 | 5.00 |
|  | **Mean** | **Std-deviation** |
| (Total) Scotland + England | 10.67 | 22.39 |
| **Base** | **60.00** | |

| **4d) When you are initiating pharmacotherapy with Dexamphetamine please provide the initial dose.** | | |
| --- | --- | --- |
|  | **N** | **%** |
| NA | 38 | 63.33 |
| 1.00 | 1 | 1.67 |
| 2.00 | 1 | 1.67 |
| 2.50 | 1 | 1.67 |
| 5.00 | 4 | 6.67 |
| 10.00 | 13 | 21.67 |
| 15.00 | 2 | 3.33 |
|  | **Mean** | **Std-deviation** |
| (Total) Scotland + England | 3.09 | 4.67 |
| **Base** | **60.00** | |

| **4d) When you are initiating pharmacotherapy with Dexamphetamine please provide the doses used during a titration phase.** | | |
| --- | --- | --- |
|  | **N** | **%** |
| NA | 38.00 | 63.33 |
| 5.00 | 3.00 | 5.00 |
| 10.00 | 6.00 | 10.00 |
| 15.00 | 3.00 | 5.00 |
| 20.00 | 8.00 | 13.33 |
| 30.00 | 2.00 | 3.33 |
|  | **Mean** | **Std-deviation** |
| (Total) Scotland + England | 5.67 | 8.66 |
| **Base** | **60.00** | |

| **4d) When you are initiating pharmacotherapy with Dexamphetamine please provide the doses used during a titration phase.** | | |
| --- | --- | --- |
|  | **N** | **%** |
| NA | 39.00 | 65.00 |
| 5.00 | 1.00 | 1.67 |
| 7.50 | 1.00 | 1.67 |
| 10.00 | 5.00 | 8.33 |
| 15.00 | 3.00 | 5.00 |
| 20.00 | 2.00 | 3.33 |
| 30.00 | 6.00 | 10.00 |
| 40.00 | 1.00 | 1.67 |
| 45.00 | 2.00 | 3.33 |
|  | **Mean** | **Std-deviation** |
| (Total) Scotland + England | 7.63 | 12.77 |
| **Base** | **60.00** | |

| **4d) When you are initiating pharmacotherapy with Dexamphetamine please provide the dose used during a titration phase.** | | |
| --- | --- | --- |
|  | **%** | **Cum.** |
| NA | 40.00 | 66.67 |
| 5.00 | 1.00 | 1.67 |
| 10.00 | 4.00 | 6.67 |
| 12.00 | 1.00 | 1.67 |
| 15.00 | 1.00 | 1.67 |
| 20.00 | 2.00 | 3.33 |
| 30.00 | 2.00 | 3.33 |
| 40.00 | 6.00 | 10.00 |
| 60.00 | 2.00 | 3.33 |
| 80.00 | 1.00 | 1.67 |
|  | **Mean** | **Std-deviation** |
| (Total) Scotland + England | 10.20 | 18.57 |
| **Base** | **60.00** | |

| **4d) When you are initiating pharmacotherapy with Dexamphetamine please provide the maintenance dose.** | | |
| --- | --- | --- |
|  | **N** | **%** |
| NA | 38.00 | 63.33 |
| 5.00 | 1.00 | 1.67 |
| 10.00 | 1.00 | 1.67 |
| 15.00 | 2.00 | 3.33 |
| 25.00 | 1.00 | 1.67 |
| 30.00 | 4.00 | 6.67 |
| 40.00 | 7.00 | 11.67 |
| 45.00 | 1.00 | 1.67 |
| 60.00 | 5.00 | 8.33 |
|  | **Mean** | **Std-deviation** |
| (Total) Scotland + England | 13.58 | 20.57 |
| **Base** | **60.00** | |

| **4e) When you are initiating pharmacotherapy with 'Other' drug please provide the initial dose.** | | |
| --- | --- | --- |
|  | **N** | **%** |
| NA | 35.00 | 58.33 |
| 0.50 | 1.00 | 1.67 |
| 1.00 | 2.00 | 3.33 |
| 2.00 | 4.00 | 6.67 |
| 5.00 | 5.00 | 8.33 |
| 10.00 | 3.00 | 5.00 |
| 12.00 | 1.00 | 1.67 |
| 15.00 | 1.00 | 1.67 |
| 25.00 | 1.00 | 1.67 |
| 50.00 | 5.00 | 8.33 |
| 75.00 | 1.00 | 1.67 |
| 100.00 | 1.00 | 1.67 |
|  | **Mean** | **Std-deviation** |
| (Total) Scotland + England | 9.04 | 20.44 |
| **Base** | **60.00** | |

| **4e) When you are initiating pharmacotherapy with 'Other' drug please provide the doses used during a titration phase.** | | |
| --- | --- | --- |
|  | **N** | **%** |
| NA | 37.00 | 61.67 |
| 1.00 | 1.00 | 1.67 |
| 2.00 | 1.00 | 1.67 |
| 4.00 | 2.00 | 3.33 |
| 5.00 | 1.00 | 1.67 |
| 7.50 | 3.00 | 5.00 |
| 10.00 | 3.00 | 5.00 |
| 15.00 | 1.00 | 1.67 |
| 18.00 | 1.00 | 1.67 |
| 20.00 | 3.00 | 5.00 |
| 50.00 | 2.00 | 3.33 |
| 100.00 | 4.00 | 6.67 |
| 150.00 | 1.00 | 1.67 |
|  | **Mean** | **Std-deviation** |
| (Total) Scotland + England | 13.53 | 31.57 |
| **Base** | **60.00** | |

| **4e) When you are initiating pharmacotherapy with 'Other' drug please provide the doses used during a titration phase.** | | |
| --- | --- | --- |
|  | **N** | **%** |
| NA | 39.00 | 65.00 |
| 1.00 | 1.00 | 1.67 |
| 5.00 | 1.00 | 1.67 |
| 6.00 | 2.00 | 3.33 |
| 10.00 | 5.00 | 8.33 |
| 15.00 | 1.00 | 1.67 |
| 20.00 | 2.00 | 3.33 |
| 30.00 | 1.00 | 1.67 |
| 36.00 | 1.00 | 1.67 |
| 50.00 | 1.00 | 1.67 |
| 75.00 | 1.00 | 1.67 |
| 150.00 | 4.00 | 6.67 |
| 225.00 | 1.00 | 1.67 |
|  | **Mean** | **Std-deviation** |
| (Total) Scotland + England | 18.98 | 47.11 |
| **Base** | **60.00** | |

| **4e) When you are initiating pharmacotherapy with 'Other' drug please provide the doses used during a titration phase.** | | |
| --- | --- | --- |
|  | **N** | **%** |
| NA | 44 | 1.67 |
| 1.00 | 1 | 73.33 |
| 5.00 | 1 | 1.67 |
| 10.00 | 2 | 5.00 |
| 15.00 | 3 | 1.67 |
| 20.00 | 3 | 1.67 |
| 100.00 | 1 | 3.33 |
| 150.00 | 2 | 5.00 |
| 200.00 | 2 | 3.33 |
| 300.00 | 1 | 3.33 |
|  | **Mean** | **Std-deviation** |
| (Total) Scotland + England | 20.52 | 58.49 |
| **Base** | **60.00** | |

| **4e) When you are initiating pharmacotherapy with 'Other' drug please provide the maintenance dose.** | | |
| --- | --- | --- |
|  | **N** | **%** |
| NA | 36.00 | 1.67 |
| 1.00 | 1.00 | 6.67 |
| 2.00 | 1.00 | 60.00 |
| 6.00 | 2.00 | 6.67 |
| 15.00 | 4.00 | 5.00 |
| 20.00 | 3.00 | 3.33 |
| 30.00 | 2.00 | 1.67 |
| 36.00 | 1.00 | 6.67 |
| 50.00 | 2.00 | 3.33 |
| 100.00 | 4.00 | 3.33 |
| 150.00 | 4.00 | 1.67 |
|  | **Mean** | **Std-deviation** |
| (Total) Scotland + England | 22.18 | 43.33 |
| **Base** | **60.00** | |

| **5) When you are initiating pharmacotherapy (Methylphenidate) how often do you see a patient (during the titration phase) until the patient reaches his/her maintenance dose?** | **N** | **%** |
| --- | --- | --- |
| Once | 5.00 | 8.33 |
| Twice | 9.00 | 15.00 |
| Three times | 26.00 | 43.33 |
| Four times | 16.00 | 26.67 |
| Five times | 2.00 | 3.33 |
| Six times | 2.00 | 3.33 |
|  | **Mean** | **Std-deviation** |
| (Total) Scotland + England | 3.12 | 1.09 |
| **Base** | **60.00** | |

| **5) When you are initiating pharmacotherapy (Atomoxetine) how often do you see a patient (during the titration phase) until the patient reaches his/her maintenance dose?** | **N** | **%** |
| --- | --- | --- |
| Once | 7 | 11.67 |
| Twice | 13 | 21.67 |
| Three times | 16 | 26.67 |
| Four times | 19 | 31.67 |
| Six times | 2 | 3.33 |
| N/A | 3 | 5.00 |
|  | **Mean** | **Std-deviation** |
| (Total) Scotland + England | 2.96 | 1.18 |
| **Base** | **60.00** | |

| **5) When you are initiating pharmacotherapy (Lisdexamphetamine) how often do you see a patient (during the titration phase) until the patient reaches his/her maintenance dose?** | **N** | **%** |
| --- | --- | --- |
| Once | 4.00 | 6.67 |
| Twice | 5.00 | 8.33 |
| Three times | 11.00 | 18.33 |
| Four times | 9.00 | 15.00 |
| N/A | 31.00 | 51.67 |
|  | **Mean** | **Std-deviation** |
| (Total) Scotland + England | 2.86 | 1.03 |
| **Base** | **60.00** | |

| **5) When you are initiating pharmacotherapy (Other drug) how often do you see a patient (during the titration phase) until the patient reaches his/her maintenance dose?** | **N** | **%** |
| --- | --- | --- |
| Once | 5.00 | 8.33 |
| Twice | 4.00 | 6.67 |
| Three times | 14.00 | 23.33 |
| Four times | 10.00 | 16.67 |
| Five times | 2.00 | 3.33 |
| N/A | 25.00 | 41.67 |
|  | **Mean** | **Std-deviation** |
| (Total) Scotland + England | 3.00 | 1.11 |
| **Base** | **60.00** | |

| **6) How often do you see a typical (Methylphenidate) adult patient with ADHD per year during a maintenance phase?** | **N** | **%** |
| --- | --- | --- |
| Once | 10.00 | 16.67 |
| Twice | 23.00 | 38.33 |
| Three times | 9.00 | 15.00 |
| Four times | 14.00 | 23.33 |
| Five times | 1.00 | 1.67 |
| Six times | 1.00 | 1.67 |
| Seven times | 1.00 | 1.67 |
| Ten times | 1.00 | 1.67 |
|  | **Mean** | **Std-deviation** |
| (Total) Scotland + England | 2.78 | 1.61 |
| **Base** | **60.00** | |

| **6) How often do you see a typical (Atomoxetine) adult patient with ADHD per year during a maintenance phase?** | **N** | **%** |
| --- | --- | --- |
| Once | 11.00 | 18.33 |
| Twice | 21.00 | 35.00 |
| Three times | 11.00 | 18.33 |
| Four times | 10.00 | 16.67 |
| Five times | 1.00 | 1.67 |
| Six times | 2.00 | 3.33 |
| Ten times | 1.00 | 1.67 |
| N/A | 3.00 | 5.00 |
|  | **Mean** | **Std-deviation** |
| (Total) Scotland + England | 2.68 | 1.58 |
| **Base** | **60.00** | |

| **6) How often do you see a typical (Lisdexamphetamine) adult patient with ADHD per year during a maintenance phase?** | **N** | **%** |
| --- | --- | --- |
| Once | 3.00 | 5.00 |
| Twice | 15.00 | 25.00 |
| Three times | 5.00 | 8.33 |
| Four times | 7.00 | 11.67 |
| Ten times | 1.00 | 1.67 |
| N/A | 29.00 | 48.33 |
|  | **Mean** | **Std-deviation** |
| (Total) Scotland + England | 2.77 | 1.65 |
| **Base** | **60.00** | |

| **6) How often do you see a typical (Other) adult patient with ADHD per year during a maintenance phase?** | **N** | **%** |
| --- | --- | --- |
| Once | 5.00 | 8.33 |
| Twice | 9.00 | 13.33 |
| Three times | 9.00 | 15.00 |
| Four times | 10.00 | 16.67 |
| Six times | 1.00 | 1.67 |
| N/A | 26.00 | 43.33 |
|  | **Mean** | **Std-deviation** |
| (Total) Scotland + England | 2.82 | 1.19 |
| **Base** | **60.00** | |

| **7) When you are initiating pharmacotherapy in your practice, are there visits led by a nurse or someone else?** | **N** | **%** |
| --- | --- | --- |
| No | 37.00 | 61.67 |
| Yes | 23.00 | 38.33 |
| Total | **60.00** | |

| **8) Who would that be?** | **N** | **%** |
| --- | --- | --- |
| **Summary of coding** |  |  |
| Nurse | 15.00 | 38.46 |
| Community Psychiatric Nurse | 6.00 | 15.39 |
| Psychologist | 5.00 | 12.82 |
| Psychiatrist (other) | 4.00 | 10.26 |
| Doctor (other) | 4.00 | 10.26 |
| Community Nurse | 2.00 | 5.13 |
| Case Manager | 1.00 | 2.56 |
| OT | 1.00 | 2.56 |
| Non-medical prescriber | 1.00 | 2.56 |
| **Base** | **23.00** | |

| **9) After how many weeks would the prescribing of ADHD pharmacotherapy of an adult patient with ADHD be continued by a general practitioner (GP)?** | **N** | **%** |
| --- | --- | --- |
| 1 week | 1.00 | 1.67 |
| 2 weeks | 2.00 | 3.33 |
| 4 weeks | 3.00 | 5.00 |
| 5 weeks | 1.00 | 1.67 |
| 6weeks | 3.00 | 5.00 |
| 8 weeks | 7.00 | 11.67 |
| 12 weeks | 13.00 | 21.67 |
| 13 weeks | 2.00 | 3.33 |
| 16 weeks | 5.00 | 8.33 |
| 24 weeks | 4.00 | 6.67 |
| 26 weeks | 1.00 | 1.67 |
| 36 weeks | 1.00 | 1.67 |
| 40 weeks | 1.00 | 1.67 |
| 52 weeks | 3.00 | 5.00 |
| 52 + weeks | 3.00 | 5.00 |
| Never | 10.00 | 16.67 |
|  | **Mean** | **Std-deviation** |
| (Total) Scotland + England | **12.67** | **15.14** |
| **Base** | **60.00** | |

| **10) Who is making the decision whether a patient's pharmacotherapy (including prescribed strengths) is adequate for a patient? Is this the sole responsibility of a treating specialist or can a nurse or someone else also be involved in this?** | **N** | **%** |
| --- | --- | --- |
| **Summary of coding** |  |  |
| The treating specialist | 30.00 | 76.92 |
| Patient | 7.00 | 17.95 |
| specialist nurse | 7.00 | 17.95 |
| Family | 5.00 | 12.82 |
| Psychologist | 1.00 | 2.56 |
| GP | 4.00 | 10.26 |
| The team | 2.00 | 5.12 |
| **Total** | **60.00** | |

| **11) Are you satisfied with the currently available treatment options for the treatment of ADHD in adult patients?** | **N** | **%** |
| --- | --- | --- |
| Yes | 34.00 | 56.67 |
| No | 26.00 | 43.33 |
| Total | **60.00** | |

| **11b) Why do you feel unsatisfied with the currently available treatment options for the treatment of ADHD in adult patients?** | **N** | **%** |
| --- | --- | --- |
| **Summary of coding** |  |  |
| Side effects | 12.00 | 23.53 |
| More options required | 12.00 | 23.53 |
| Lack of efficacy | 10.00 | 19.61 |
| Abuse / addiction potential | 9.00 | 17.65 |
| More long acting | 2.00 | 3.92 |
| Diversion risk | 2.00 | 3.92 |
| The condition is underdiagnosed. Health services are not geared up to provide the resources to address the needs of this group of patients Also there is not adequate awareness of professionals in primary care and among psychiatric services of the condition . | 1.00 | 1.96 |
| Risk of misdiagnosis | 1.00 | 1.96 |
| Fiddly prescribing, GP reluctance to coninue | 1.00 | 1.96 |
| Too much monitoring required | 1.00 | 1.96 |
| **Total** | **26.00** | |

| **12) I your clinical practice have you been using the AISRS questionnaire/tool to evaluate your adult patients with ADHD?** | **N** | **%** |
| --- | --- | --- |
| No | 41.00 | 68.33 |
| Yes | 19.00 | 31.67 |
| Total | **60.00** | |

| **13) What is your opinion on the interchangeability of the ADHD-RS-IV and the AISRS questionnaires?** | **N** | **%** |
| --- | --- | --- |
| Prefer AISRS | 5.00 | 33.33 |
| Prefer ADHD-RS-IV | 1.00 | 6.67 |
| Both useful tools | 10.00 | 52.63 |
| No opinion | 3.00 | 15.79 |
| **Total** | **19.00** | |

| **Thank you for taking part in this survey. If you would like to make any further comments about the survey or its contents, please write them here.** |
| --- |
| Interesting survey. Important issue in my practice. |
| Monitoring the response and deciding suitable parameters is difficult |
| I use Browns Add and a computerised program to assess response; TOVA |
| Limited experience with issuing Dexamphetamine. I use PRN methylphenidate to get a baseline measure of need/symptom control / side effects. Prefer Strattera when there is abuse potential though patients don't as perception it is too slow to act. |
| I always prescribe sustained release methylphenidate (concerta, medikinet or equasym) and try to find drug which works best for patient and follow manufacturers titration guidance for each drug |
| We have been using the DIVA questionnaire + copies of school reports. |
| I use diva 2 questionnaIre. But it is more of a clinical decision. |

**Appendix A – Questionnaire**

| A. Are you a psychiatrist?  B. Where in the UK are you currently practicing?  C. Are you treating adult patients (18 years of age and above) with ADHD?  D. How many adults with ADHD (new and existing) do you see per month?  Question 1) What percentage of your adult patients who are receiving treatment for ADHD are on pharmacotherapy?  Question 2) What percentage of your adult patients who are diagnosed with ADHD are not receiving any form of treatment at all? |  |
| --- | --- |
| Question 3) When you are initiating pharmacotherapy for an average adult patient with ADHD, in what percentage of patients do you only prescribe one drug (versus a combination therapy of two drugs)?  Question 3b) On average, what percentage of patients who are receiving pharmacotherapy for the first time (first line) are indicated on which drug and what is the average dose used for these patients? Methylphenidate  Question 3b) On average, what percentage of patients who are receiving pharmacotherapy for the first time (first line) are indicated on which drug and what is the average dose used for these patients? Atomoxetine  Question 3b) On average, what percentage of patients who are receiving pharmacotherapy for the first time (first line) are indicated on which drug and what is the average dose used for these patients? Lisdexamphetamine  Question 3b) On average, what percentage of patients who are receiving pharmacotherapy for the first time (first line) are indicated on which drug and what is the average dose used for these patients? Dexamphetamine  Question 3b) On average, what percentage of patients who are receiving pharmacotherapy for the first time (first line) are indicated on which drug and what is the average dose used for these patients? Other  Question 3c) On average, what percentage of patients who are 2nd line patients are you prescribing which drug and what is the average dose used for these patients? Methylphenidate  Question 3c) On average, what percentage of patients who are 2nd line patients are you prescribing which drug and what is the average dose used for these patients? Atomoxetine  Question 1 3c) On average, what percentage of patients who are 2nd line patients are you prescribing which drug and what is the average dose used for these patients? Lisdexamphetamine  Question 3c) On average, what %age of patients who are 2nd line patients are you prescribing which drug and what is the average dose used for these patients? Dexamphetamine  Question 3c) On average, what %age of patients who are 2nd line patients are you prescribing which drug and what is the average dose used for these patients? Other  Question 4) In adult patients with ADHD in whom you are initiating pharmacotherapy with a combination therapy, what combinations are you using in which strength? Combination A  Question 4) In adult patients with ADHD in whom you are initiating pharmacotherapy with a combination therapy, what combinations are you using in which strength? Combination B  Question 4) In adult patients with ADHD in whom you are initiating pharmacotherapy with a combination therapy, what combinations are you using in which strength? Combination C  Question 4a) When you are initiating pharmacotherapy with Methylphenidate please provide the initial dose.  Question 4a) When you are initiating pharmacotherapy with Methylphenidate please provide the doses used during a titration phase.  Question 4a) When you are initiating pharmacotherapy with Methylphenidate please provide the maintenance dose.  Question 4b) When you are initiating pharmacotherapy with Atomoxetine please provide the initial dose.  Question 4b) When you are initiating pharmacotherapy with Atomoxetine please provide the doses used during a titration phase.  Question 4b) When you are initiating pharmacotherapy with Atomoxetine please provide the maintenance dose.  Question 4c) When you are initiating pharmacotherapy with Lisdexamphetamine please provide the initial dose.  Question 4c) When you are initiating pharmacotherapy with Lisdexamphetamine please provide the doses used during a titration phase.  Question 4c) When you are initiating pharmacotherapy with Lisdexamphetamine please provide the maintenance dose.  Question 4d) When you are initiating pharmacotherapy with Dexamphetamine please provide the initial dose.  Question 4d) When you are initiating pharmacotherapy with Dexamphetamine please provide the doses used during a titration phase.  Question 4d) When you are initiating pharmacotherapy with Dexamphetamine please provide the maintenance dose.  Question 4e) When you are initiating pharmacotherapy with 'Other' drug please provide the initial dose.  Question 4e) When you are initiating pharmacotherapy with 'Other' drug please provide the doses used during a titration phase.  Question 4e) When you are initiating pharmacotherapy with 'Other' drug please provide the maintenance dose.  Question 5) When you are initiating pharmacotherapy how often do you see a patient (during the titration phase) until the patient reaches his/her maintenance dose? Methylphenidate  Question 5) When you are initiating pharmacotherapy how often do you see a patient (during the titration phase) until the patient reaches his/her maintenance dose? Atomoxetine  Question 5) When you are initiating pharmacotherapy how often do you see a patient (during the titration phase) until the patient reaches his/her maintenance dose? Lisdexamphetine  Question 5) When you are initiating pharmacotherapy how often do you see a patient (during the titration phase) until the patient reaches his/her maintenance dose? Other  Question 6) How often do you see a typical adult patient with ADHD per year during a maintenance phase? Methylphenidate  Question 6) How often do you see a typical adult patient with ADHD per year during a maintenance phase? Atomoxetine  Question 6) How often do you see a typical patient with ADHD per year during a maintenance phase? Lisdexamphetine  Question 6) How often do you see a typical adult patient with ADHD per year during a maintenance phase? Other  Question 7) When you are initiating pharmacotherapy in your practice, are there visits led by a nurse or someone else?  Question 8) Who would that be?  Question 9) After how many weeks would the prescribing of ADHD pharmacotherapy of an adult patient with ADHD be continued by a general practitioner (GP)?  Question 10) Who is making the decision whether a patient's pharmacotherapy (including prescribed strengths) is adequate for a patient? Is this the sole responsibility of a treating specialist or can a nurse or someone else also be involved in this?  Question 11) Are you satisfied with the currently available treatment options for the treatment of ADHD in adult patients?  Question 11b) Why do you feel unsatisfied with the currently available treatment options for the treatment of ADHD in adult patients?  Question 12) I your clinical practice have you been using the AISRS questionnaire/tool to evaluate your adult patients with ADHD?  Question 12) I your clinical practice have you been using the AISRS questionnaire/tool to evaluate your adult patients with ADHD?  Question 13) What is your opinion on the inter-changeability of the ADHD-RS-IV and the AISRS questionnaires?  Thank you for taking part in this survey. If you would like to make any further comments about the survey or its contents, please write them here. |  |
|  |  |
|  |  |
